# Positive Thinking: Countercation Effects in Colloidal Syntheses of Gold Nanoparticles

**DOI:** 10.1021/acs.nanolett.5c04815

**Published:** 2025-10-10

**Authors:** Kristian Junker Andersen, Márton Varga, Aleksandra Smolska, Gregory Nordhal, Jonas H. Jensen, Rodrigo Moreno, Espen D. Bøjesen, Andy S. Anker, Jonathan Quinson

**Affiliations:** † Biological and Chemical Engineering Department, 1006 Aarhus University, 8200 Aarhus, Denmark; ‡ Interdisciplinary Nanoscience Center (iNANO) and Center for Sustainable Energy Materials (CENSEMAT), 1006 Aarhus University, 8000 Aarhus, Denmark; § Data Systems, and Robotics Section, 100335 IT University of Copenhagen, 2300 Copenhagen, Denmark; ∥ Department of Energy Conversion and Storage, Technical University of Denmark, Kgs. Lyngby 2800, Denmark; ⊥ Department of Chemistry, University of Oxford, Oxford OX1 3TA, United Kingdom; # CICA-Centro Interdisciplinar de Química e Bioloxía, Facultade de Ciencias, Universidade da Coruña, Campus de Elviña, 15008 A Coruña, Spain

**Keywords:** Gold, Nanoparticles, Cations, Citrate, Borohydride, Hydroxide, Colloids

## Abstract

Gold nanoparticles (Au NPs) are intensively studied and widely applicable to catalysis, sensing, medical applications, and many more. In particular, citrate- and borohydride- mediated colloidal syntheses of Au NPs are extremely popular. While it can be reasonably expected that countercations have a role to play, there is surprisingly almost no study on the effect of countercations in citrate- and borohydride-mediated colloidal syntheses of Au NPs. It is here shown that the countercation (Li^+^, Na^+^, K^+^) from citrate, borohydride, but also from hydroxide species, plays an overlooked role in the stabilization of gold colloidal dispersions. The stability, size, and degree of shape control over the NP decrease in the order Li^+^ > Na^+^ > K^+^, due to a stronger interaction between the smaller cations and metal surfaces. The findings are directly relevant for further fundamental studies, an improved control of the syntheses and scale-up.

## Graphical abstract

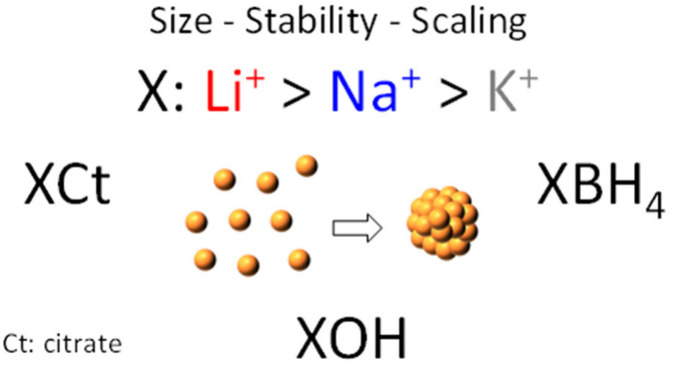

Gold (Au) nanoparticles (NPs) present a unique set of properties relevant for numerous applications including medical applications, sensing, water treatment, or catalysis, e.g., for chemical production and/or energy conversion.^1,2^ A vast literature covers the controlled synthesis of Au NPs.^1,3^ The most popular syntheses are probably those requiring citrate-based chemicals, reported as early as 1934 by Borowskaja.^4,5^ The method, revisited in 1951 by Turkevich,^6^ and now best known as the Turkevich–Frens method, is probably the most widely reported and studied approach to synthesize colloidal Au NPs.^3,5,7^ The second most popular synthesis is probably the so-called Brust–Schiffrin method where borohydrides are used as reducing agents.^8,9^ An account for the general protocols, the development, and the importance of those syntheses is proposed in the Supporting Information (SI), sections 1–3 (S1–S3).

Both reactions have been used as model systems to provide insights into Au NP formation.^7,10^ While it is experimentally simple to carry out those syntheses, a detailed understanding of the formation of the Au NPs via those iconic methodologies is surprisingly relatively poorly established, in terms of the chemical reactions occurring, at the levels of the formation pathway(s) and stabilization of the nanocrystals, and therefore when it comes to subsequent scaling strategies.^7,11^ This can be attributed to the multiple roles that the relatively few chemicals needed can play during the reaction. There is therefore still much to learn from these syntheses, as well as from emerging alternatives, to provide a comprehensive picture and full control over colloidal syntheses of Au NPs.

In both the worldwide used and studied citrate- and borohydride-mediated approaches, an almost exclusive focus has been given to the use of sodium citrate (NaCt) and sodium borohydride (NaBH_4_), respectively; see details in S1–S2. We could not identify any unified study where the effect of the countercation was investigated. We are only aware of one work from 2016, a Bachelor’s thesis from A. B. Closson, University of Maine, USA,^12^ that touches upon the formation pathway of Au NPs when lithium citrate (LiCt), NaCt, or potassium citrate (KCt) are used. The formation pathway studied from a seed mediated strategy is reported to differ slightly with the different cations, and an aggregation occurs for NaCt and KCt but not LiCt. The results suggest that the Au NPs are relatively more stable in the presence of Li^+^ ions. However, we could not find any follow up publications from this Bachelor’s thesis. Given the importance of citrate-based and borohydride-based syntheses in the field of Au NPs, and considering that the Turkevich–Frens and Brust–Schiffrin syntheses have been extensively refined over the last 70 and 30 years, respectively, the influence of the countercation is a surprisingly overlooked parameter.

The lack of investigation and/or interest to date on the effect(s) of cations in colloidal syntheses of Au NPs is also surprising because it can be expected that noncovalent interactions between alkali-cations and a gold surface decrease in the order Li^+^ > Na^+^ > K^+^,^13^ as supported by theoretical calculations,^13^ and as demonstrated experimentally for other metals,^14^ detailed in S4. Inspiration can also be drawn from the Hofmeister series or lyotropic series.^15^ It is expected that the degree of aggregation will increase in the order Li^+^ < Na^+^ < K^+^.

We hypothesize that using chemicals containing Li^+^, Na^+^, or K^+^ leads to significantly different outcomes, for instance, on the stability of the colloids. We established the effects of cations and various citrate-based syntheses, expanding this observation to various BH_4_-based syntheses and various alcohol-mediated syntheses of Au NPs performed in the presence of hydroxides, detailed in S3, as well as hybrid syntheses of those strategies. We here illustrate the benefits of using Li-based syntheses for improved control over the Au NP properties, colloidal stability, reproducibility, and/or scale up. The results open new opportunities for more controlled and scalable colloidal syntheses of nanomaterials.

## Syntheses

The overall procedures are detailed in SI. The citrate-mediated syntheses follow the general procedure of the Borowskaja–Turkevich–Frens method,^6,7,16^ but induced using a 365 nm light for reasons detailed in SI. The borohydride-based synthesis was inspired by previous work using NaBH_4_ or LiBH_4_.^9,17^ The so-called surfactant-free alcohol-mediated synthesis under alkaline conditions followed the general recipe detailed elsewhere and in SI.^18,19^

## Citrate-Based Syntheses

For Au NP syntheses, citrate is reported to play the role of reducing agent, stabilizer, and/or pH buffer.^7^ A parameter considered to control the size of the Au NPs is the (Na)­Ct/Au molar ratio, which controls the pH of the solution, where too low or too high values lead to larger and/or unstable Au NPs. A typical NaCt/Au molar ratio is around 0–20.^20^ The synthesis is typically performed in the range of 0.1–0.5 mM of HAuCl_4_ to ensure the formation of stable Au NPs.^7^ Details about the experiments and characterization performed are given in S5. A concentration of 0.5 mM HAuCl_4_ is here preferred for its relatively high value, directly relevant to improve the signal-to-noise ratio in various characterization methods and/or the scalability of the synthesis since higher gold concentrations lead to a lower amount of chemicals and lower volumes of solvents to produce the same mass of gold NPs.^18^ As opposed to classical approaches, and as detailed in SI, we do not use a thermally induced synthesis but prefer a UV-induced synthesis to allow a screening of a larger parameter space using smaller volumes (2–3 mL) and hence limit the waste generated.^21,22^

## Influence of XCt/Au Molar Ratio

We first investigate the influence of using XCt with X = Li, Na, K, with different XCt/Au molar ratios at a given concentration of HAuCl_4_ of 0.5 mM, with results reported in Figures S4 and S5. For XCt/Au molar ratios below ca. 20, there is not a strong influence of the XCt/Au ratio when KCt, NaCt, or LiCt are used and the NPs are around 10 nm. Increasing the XCt/Au molar ratio ultimately leads to larger NPs with poorly defined shapes when KCt is used, and even to nonstable colloids for the higher KCt/Au molar ratios around 30; see Figure 1. Using NaCt leads to stable colloids even at higher NaCt/Au molar ratios, but the NPs tend to be larger as the NaCt/Au molar ratio increases. Using LiCt does not lead to any significant changes, and small spherical NPs around 8 nm are obtained in all cases. Those trends were confirmed with temperature-induced syntheses detailed in S7. Although the formation pathway of the Au NPs by citrate-mediated syntheses differs slightly depending on how the synthesis is induced (thermally or UV–vis),^23,24^ we did observe in both cases an effect of the cation. Those results show that it is easier to obtain smaller NPs with a well-defined shape across a wider range of XCt/Au molar ratios in the relative order of LiCt > NaCt > KCt.

**Figure 1:**
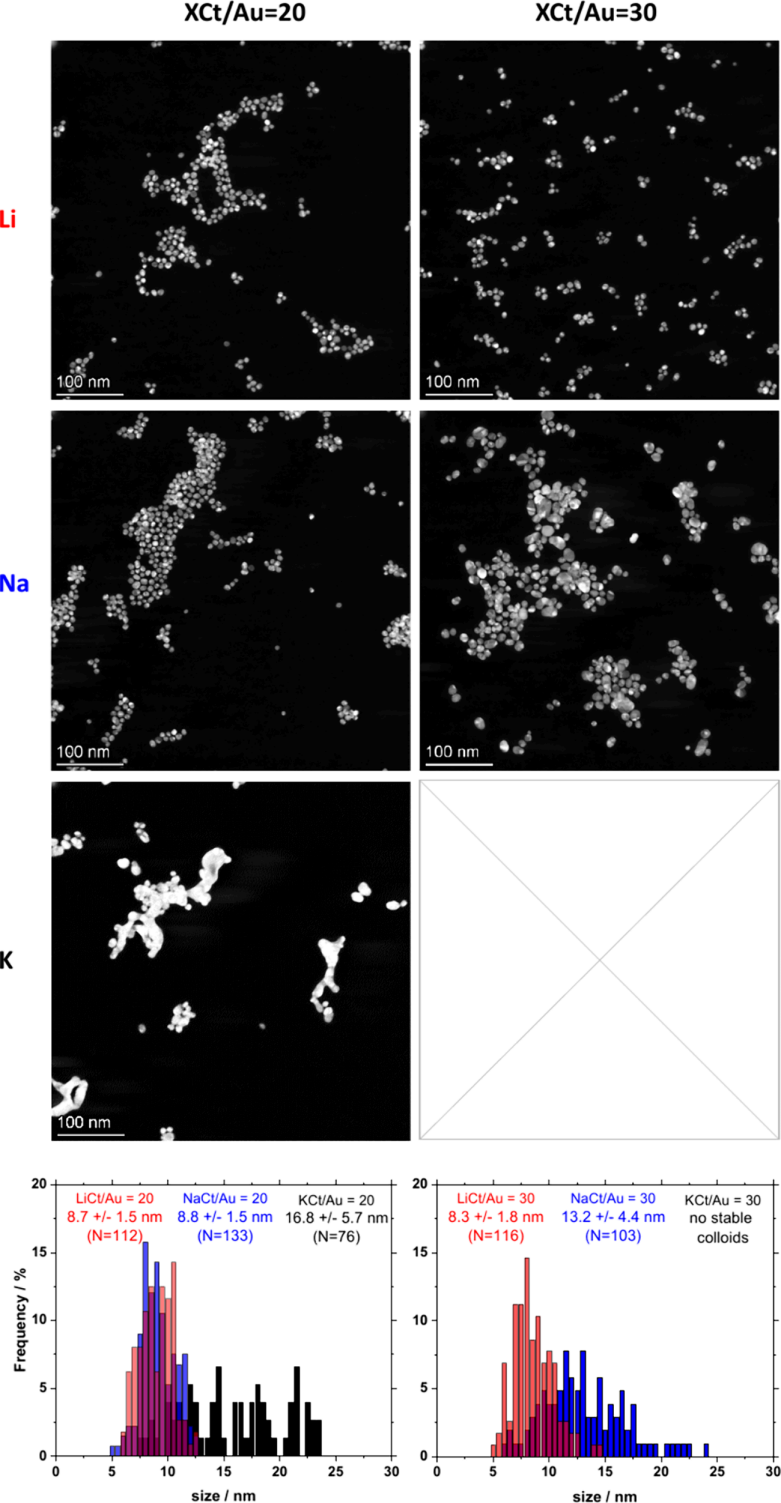
STEM micrographs and related size distribution of Au NPs obtained using 0.5 mM HAuCl_4_ and an XCt/Au molar ratio of 20 or 30, as indicated, and where X = Li, Na, K, as indicated. The corresponding UV–vis data are given in Figure S4.

## XCt/Au Molar Ratio and NP Stability

A direct effect of the simple change of the countercations is the relative stability of the Au NPs that decreases in the order Li > Na > K at all XCt/Au molar ratios, although the effect is more pronounced at higher ratios, as illustrated in Figure 2 for stability tests by centrifugation. This can be attributed to the larger size of the Au NPs so obtained, which are less stable and this correlates well with various stability metrics retrieved from UV–vis detailed in SI.

**Figure 2:**
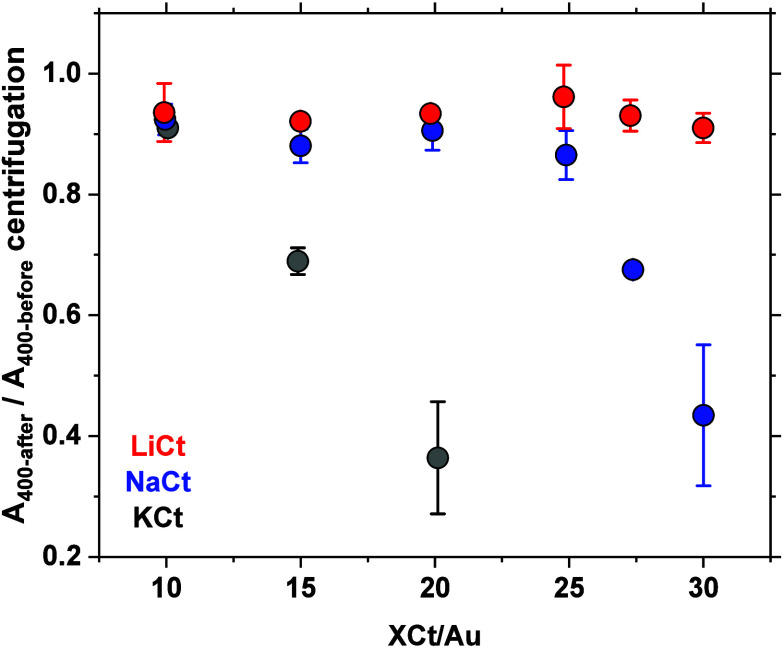
Relative stability of colloidal Au NPs obtained using different XCt/Au molar ratios and different countercations, as indicated. The data points that appear without error bars actually have error bars smaller than the size of the data point itself.

## Role of the Countercation

To understand the role of the countercation, we performed a range of time-resolved UV–vis measurements. Following the classical nucleation theory, smaller NPs are expected if a fast nucleation occurs, followed by a slow growth.^25^ The redox-potentials of LiCt, NaCt, and KCt are expected to be close. Therefore, the main effect of the countercation is expected to be more on the growth and/or stabilization phases than the nucleation.

Time-resolved UV–vis data for Au NPs obtained using different counter-cations are summarized in Figure 3, along with complementary data in Figures S6 and S7. (*A*_spr_)^1/3^ is proportional to *R*(*N*)^1/3^, where *R* is the radius of the NPs and *N* is the number of NPs.^26^ (*A*_spr_)^1/3^ was previously used to follow the formation of citrate-mediated syntheses of Au NPs and can be divided into three phases (I–III) indicated in Figure 3. In a first phase I, (*A*_spr_)^1/3^ increases in a superlinear fashion, which corresponds to growth via aggregation. In phase II, (*A*_spr_)^1/3^ increases linearly, which corresponds to a surface growth reaction. In phase III, (*A*_spr_)^1/3^ exponentially increases and levels off to its final value, which corresponds to an autocatalytic growth stage and the end of the reaction.

**Figure 3:**
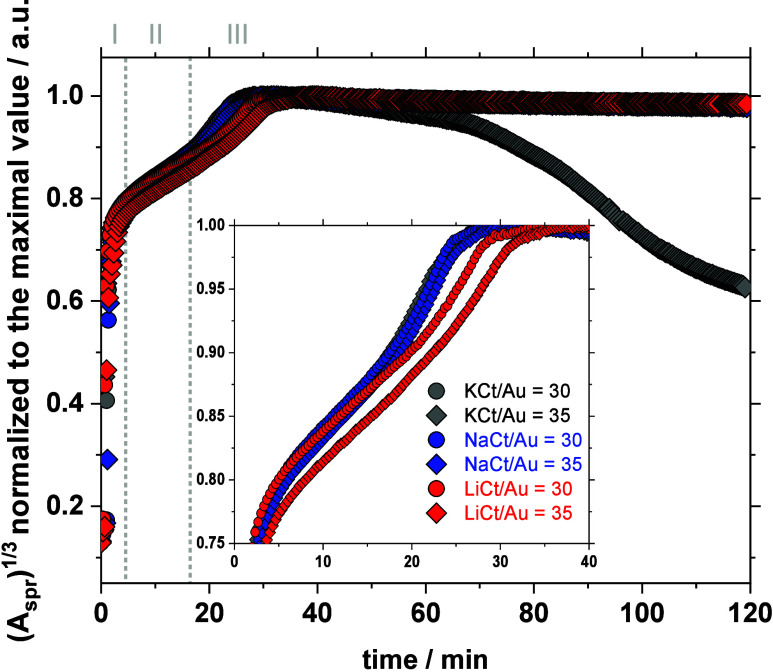
Time resolved metrics retrieved from UV–vis: (*A*_spr_)^1/3^ as a function of time. The inset is a more detailed focus on the initial stages of the reaction. The three phases (I–III) detailed in the text are indicated.

Those three phases are observed regardless of the cation present. Thus, the cations do not significantly influence the initial formation of the Au NPs (I) but rather their stabilization (in phases II and/or III). The relative stability that is experimentally observed follows the decreasing order of stability Li^+^ > Na^+^ > K^+^, which matches the decrease in cation-Au noncovalent interactions with Li^+^ > Na^+^ > K^+^.^13^ This is especially clear with a KCt/Au molar ratio of 30 where the NPs are not stable, which accounts for the decrease in the (*A*_spr_)^1/3^ value over time in phase III.

A further argument to validate the role of the cations is that the formation of the Au NPs tends to be slower for Li-mediated syntheses in phase II, with no significant effect in phase I. LiCt does not favor a faster formation of the NPs (I), which, considering the classical nucleation theory, could account for the formation of smaller NPs. The formation of smaller NPs using LiCt is then to be related to growth phenomena (II and/or III). The relatively slower phase II related to a surface growth can be attributed to stronger noncovalent Li–Au interactions as the NPs are forming. Those interactions slow further NP growth while increasing the electrostatic stabilization of the Au NPs, resulting overall in smaller and more stable NPs. The slower formation of more stable (less aggregated) NPs using LiCt was also suggested in a previous BSc thesis focusing on seed-mediated studies.^12^

In good agreement with the results presented in Figure 2, the NPs prepared using LiCt tend to be more stable over time; see Figure S8.

## Increasing HAuCl_4_ Concentration

A direct consequence of those findings is the possibility to develop syntheses of Au NPs at higher precursor concentrations and yet obtain relatively stable colloids. The results obtained for various precursor concentrations and an XCt/Au molar ratio of 30 are reported in Figure S9. The maximum HAuCl_4_ precursor concentration for which stable colloids were obtained was 5 mM using LiCt, a concentration range for which using NaCt or KCt did not lead to stable colloids.

This is a significant achievement to develop more efficient syntheses of Au NPs at a concentration of precursor higher than the usual maxima around 0.5–1.0 mM.^3,7,27^ However, the stability over time of the as-prepared colloidal Au NPs is not optimal. Nevertheless, it is worth pointing out that dilution of the as-prepared Au NPs at 5mM to 3 mM Au equivalent results in them being stable for at least a month; see Figure S10. A benefit of being able to perform the syntheses at higher concentrations of gold precursor is to produce a given mass of NPs using less chemicals, less energy, with a lower footprint and in a way that is easier to process (because lower volumes are needed for the same output).

## Other Syntheses

Similar trends leading to a decreasing degree of control over the synthesis in the order Li > Na > K were obtained using various syntheses requiring various chemicals such as XCt, XBH_4_, and XOH in water (H_2_O) and/or ethanol. An overview of those syntheses is provided in the related sections, where the results are discussed and detailed in S6–S14, and is provided in Table 1. In all cases, the syntheses lead to smaller and/or more stable NPs and for a longer time across a wider experimental window when Li^+^ cations are present.

**Table 1:** Overview of Syntheses Detailed in SI where the Effect of the Countercation Is Observed for Colloidal Au NPs^a^

chemicals	section	relative benefits^a^
XCt + H_2_O (UV-induced)	SI-6	Li > Na > K
XCt + H_2_O (T-induced)	SI-7	Li > Na > K
XCt + H_2_O + Ethanol (UV-induced)	SI-8	Li > Na > K
XBH_4_ + H_2_O	SI-9	Li > Na/K
XBH_4_ + H_2_O + Ethanol	SI-10	Li > Na/K
XOH + H_2_O + Ethanol	SI-11 + 14	Li > Na > K
XOH + H_2_O + Ethanol Lower purity chemicals	SI-12	Li > Na > K
XOH + XCt + H_2_O + Ethanol	SI-13 + 14	Li > Na > K

a Considering size control toward smaller sizes and/or the width of the experimental window for which the syntheses lead to stable colloids and/or increased stability over time.

b X = Li, Na, K. RT stands for ‘room temperature’ and T for ‘temperature’.

The stabilization role of the cation is also supported in S14 where adding LiCt, NaCt of KCt to preformed Au NPs leads to unstable Au NPs when KCt is used. Another example is an account of attempts to push the concentration of gold precursors toward higher values, detailed in S15. In the case of surfactant-free ethanol-mediated syntheses, the use of LiOH leads to colloids being stable for concentrations up to 3–4 mM HAuCl_4_, whereas only 2 mM HAuCl_4_ could be successfully used when NaOH was preferred. The NP size can be controlled toward larger sizes using LiOH as the base when the precursor concentration increases, as reported in Figure S37.

## Relevance for Further Studies

A direct consequence of the findings, in particular regarding performing the syntheses at higher concentrations of gold, is the opportunity to perform various studies with a higher signal-to-noise-ratio. To date most studies, in particular time-resolved studies, on Au NPs focus on using small-angle X-ray scattering (SAXS).^7,26^ However, SAXS provides information only about the size and morphology of the Au NPs. In contrast, X-ray total scattering (TS) with pair distribution function analysis (PDF) is a powerful tool to provide new insights into NP formation from local to macroscopic order.^28–30^ However, such measurements require relatively high concentrations of metallic NPs.^29,31^

In line with the results presented above, only Li-mediated syntheses in the case of XCt-mediated syntheses could lead to colloidal dispersions stable enough at higher concentrations of Au NPs (e.g., 3 mM) as detailed in S15. For those concentrations, using NaCt did not lead to colloids stable enough for meaningful further measurements. While 2–3 mM Au concentration is very low for a standard quality TS measurement, we managed to measure PDF data with a reasonable signal-to-noise ratio, at the DanMAX beamline of the MAX IV Synchrotron, Lund, Sweden, detailed in Figure 4.

**Figure 4:**
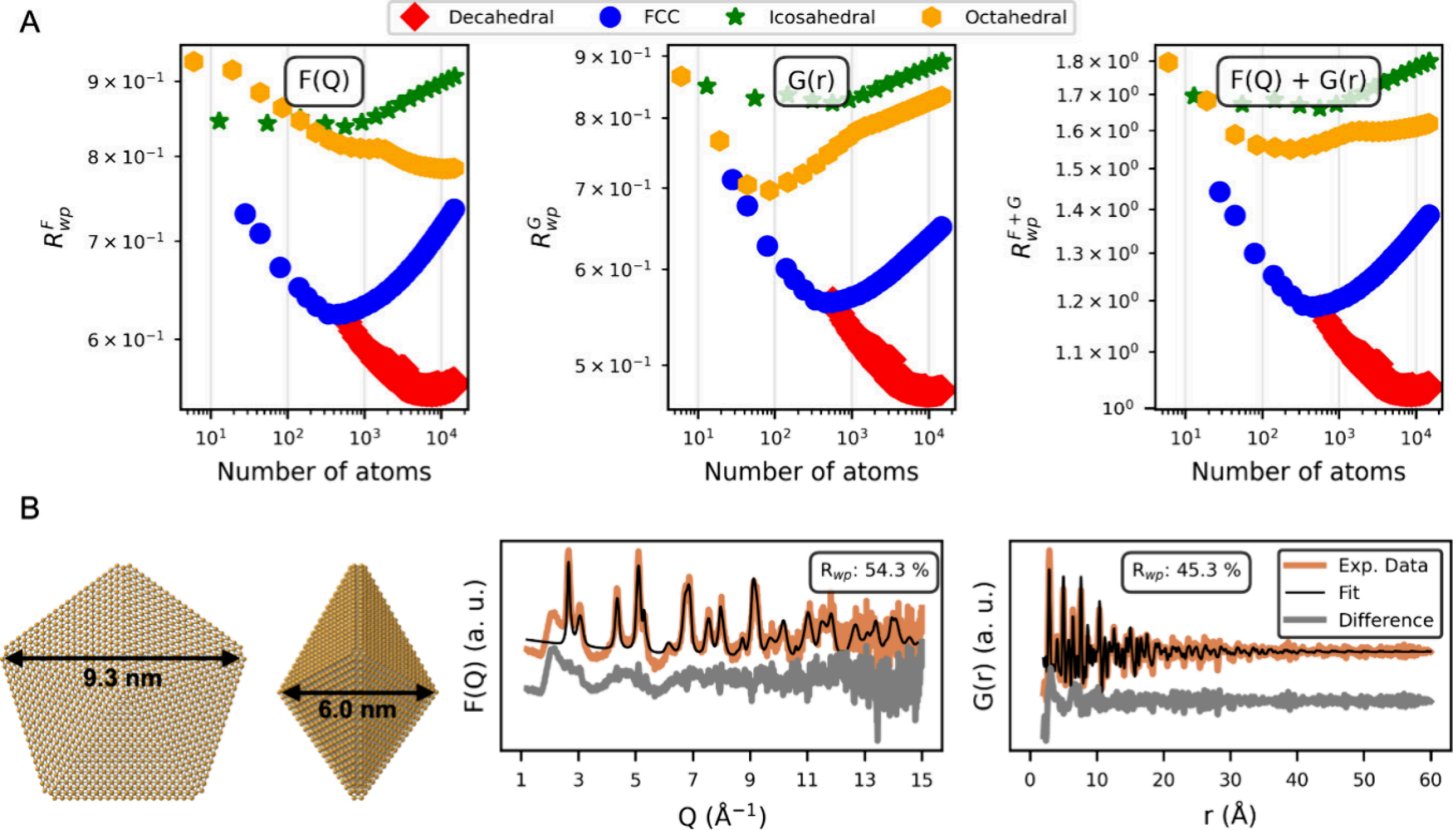
A cluster-mining approach for modeling the *F*(Q) (X-ray total scattering) and *G*(r) (PDF) data for samples obtained using 3 mM HAuCl_4_ and LiCt/Au molar ratio of 20. (A) Comparison of the best-fit results for four structural motifs, octahedral, icosahedral, decahedral, and FCC—across various cluster sizes. The *R*_wp_ metric is plotted against the total number of atoms in each model. Notably, decahedral structures (red diamonds) provide the lowest *R*_wp_ values whether fitted to the *F*(*Q*) or *G*(*r*) data or a combination, suggesting that they best describe the experimentally measured scattering data. (B) Experimental *F*(*Q*) and *G*(*r*) data (orange) overlaid with the fit of the best-fitting decahedral structure (black). The bottom panels display the difference curves (gray). Both the scaling of the *F*(*Q*) and *G*(*r*) data and the atomic displacement parameter value were fitted.

Although bulk Au typically adopts an FCC lattice, nanosized Au particles can form geometries such as icosahedral, octahedral, or decahedral morphologies,^32–34^ which differ in how atomic layers stack and how surfaces are truncated or twinned. Therefore, we had to perform an extensive cluster-mining search,^35^ identical to the search performed and detailed elsewhere,^36^ covering 1965 decahedral, icosahedral, octahedral, and FCC motifs, to identify any structures that might better describe the experimental data. Our analysis reported in Figure 4A confirms that decahedral structures outperform other structural families and that the target decahedral model ranks among the best in our cluster library for both the *F*(Q) or *G*(*r*) data or a combination hereof. The associated combined fit of the decahedral structure (9.3 nm × 9.2 nm × 6.0 nm) to both the *F*(*Q*) and *G*(*r*) data is shown in Figure 4B. The Au NPs are therefore best described by a decahedral structure with a size of ca. 10 nm.

Although the inherently low sample concentrations result in noisy data and complicate accurate background subtraction, the fitting indicates good agreement with the decahedral structure. There is a presence of a broader signal in the data that is not modeled, attributed to the presence of a secondary, smaller phase (putatively related at this stage to residual Au NP precursors and/or smaller minority clusters). The decahedra structure and presence of smaller clusters are supported by electron microscopy, detailed in S16. Nevertheless, and to the best of our knowledge, these X-ray total scattering data are the first results of the kind for colloidal Au NPs obtained in aqueous media for citrate-based synthesis. The proof-of-concept reported here opens new opportunities for time-resolved studies, e.g., by X-ray TS performed at synchrotron to shine new lights on Au NP formation,^29^ although such studies are beyond the scope of this first report.

Overall, the results highlight the largely unexplored effects of cations in the colloidal syntheses of Au NPs. As pointed out in more detail in S17, the findings reported here suggest that mixtures of cations from different chemicals and/or from the gold precursors might be overlooked knobs to tune Au NP syntheses and properties.

## Conclusion

In conclusion, it is here established that for various citrate-, borohydride-, and hydroxide-mediated colloidal syntheses of Au NPs, the countercation influences the outcome of the synthesis. For various synthetic protocols using chemicals such as XCt, XBH_4_, and/or XOH, with X = Li, Na, K, the size control over the Au NP size decreases in the order Li > Na > K, while the NPs tend to increase in the order Li < Na/K. The stability of the colloids decreases with the order Li > Na > K. This trend can be attributed to better stabilization of the Au NPs with the smallest cation Li^+^ that interacts more strongly via noncovalent interactions with metal surfaces. The results support the broad yet overlooked relevance of the nature of the cation as a simple knob to better control colloidal NP syntheses.

A direct consequence is that Li-mediated syntheses can be performed successfully at higher concentrations of the precursor. This finding opens opportunities for fundamental studies with a better signal-to-noise ratio for various measurements. The finding is also relevant to developing strategies to perform syntheses of colloidal Au NPs in aqueous media at higher concentrations than the typical 0.1–0.5 mM of HAuCl_4_ reported to date.

If Li-based chemicals help ensure better size control, stability, and/or reproducibility, the higher price of Li-based chemicals compared to Na-based chemicals must be taken into consideration. In addition, to follow the principles of *green* and *sustainable* chemistry, it must be kept in mind that Li-based chemicals tend to be more toxic than Na-based chemicals.

Nevertheless, given the wide range of reports and still ongoing research on citrate-, borohydride-, and hydroxide-mediated syntheses of NPs, it is anticipated that the attention drawn here to cation effects will lead to original research for a deeper understanding of NP formation and stabilization for Au and other (nano)­materials.

Finally, beyond synthesis and processing of the nanomaterials, the effects related to countercations are expected to have a significant impact in various fields of applications of the NPs such as catalysis, sensing, or biomedical applications.

## Supplementary Material



## Notes

Additional consideration on the literature, detailed Materials and Methods section, additional characterization methods and results, including UV–vis, STEM, and 4D-STEM data (PDF)
