# Supplementary material for: Positive Thinking: Countercation Effects in Colloidal Syntheses of Gold Nanoparticles
Source: Nano Lett. 2025 Oct 10;25(42):15436–42. doi: 10.1021/acs.nanolett.5c04815 (PMC12550839; doi:10.1021/acs.nanolett.5c04815)
Supplement: Supplementary file 1 [file nl5c04815_si_001.pdf]

Supporting information

for

Positive thinking: counter-cations effects in colloidal syntheses of gold nanoparticles

Kristian Junker Andersen,[a] Márton Varga,[a] Aleksandra Smolska,[a] Gregory Nordhal,[b] Jonas H. Jensen,[c] Rodrigo Moreno,[c] Espen D. Bøjesen,[b] Andy S. Anker,[d,e] Jonathan Quinson[f,a]*

a) Biological and Chemical Engineering Department, Aarhus University, 8200 Aarhus, Denmark
b) Interdisciplinary Nanoscience Center (iNANO) and Center for Sustainable Energy Materials (CENSEMAT); Aarhus University, 8000 Aarhus, Denmark
c) Data Systems, and Robotics Section, IT University of Copenhagen, 2300 Copenhagen, Denmark
d) Department of Energy Conversion and Storage, Technical University of Denmark, Kgs. Lyngby 2800, Denmark
e) Department of Chemistry, University of Oxford, Oxford OX1 3TA, United Kingdom
f) CICA-Centro Interdisciplinar de Química e Bioloxía, Facultade de Ciencias, Universidade da Coruña, Campus de Elviña, 15008 A Coruña, Spain

* Corresponding author: j.quinson@udc.es

Table of contents

1. Overview of literature on citrate-based syntheses of gold nanoparticles 3
2. Overview of literature on borohydride-based syntheses of gold nanoparticles 5
3. Overview of literature on mono-alcohol-based syntheses of gold nanoparticles in alkaline aqueous systems 7
4. Overview of literature on cations effect(s) in the syntheses of gold nanoparticles 8
5. Materials and methods 9
   5.1. Chemicals 9
   5.2. Synthesis 10
      5.2.1. *Green* and *Sustainable* practices 10
      5.2.2. General considerations 10
      5.2.3. Borowskaja-Turkevich-Frens synthesis of Au NPs 11
      5.2.4. Adapted Borowskaja-Turkevich-Frens synthesis of Au NPs with ethanol 11
      5.2.5. Surfactant-free BH_4-mediated synthesis of Au NPs 12
      5.2.6. Adapted surfactant-free BH_4-mediated synthesis of Au NPs with ethanol 12
      5.2.7. Surfactant-free synthesis of Au NPs in alkaline water-ethanol mixtures 12
      5.2.8. Surfactant-free synthesis of Au NPs in alkaline water-ethanol mixtures with citrate additives 13
      5.2.9. Surfactant-free synthesis of Au NPs towards higher $HAuCl_4$ concentrations 13
      5.2.10. Comment on reproducibility 14
   5.3. Characterization 15
      5.3.1. General consideration 15
      5.3.2. UV-vis 15
      5.3.3. Scanning Transmission Electron Microscope (STEM) 16
      5.3.4. X-ray scattering with pair distribution function analysis 17
      5.3.5. Centrifugation 17
6. UV-induced Borowskaja-Turkevich-Frens synthesis of Au NPs 18
7. Temperature-induced Borowskaja-Turkevich-Frens synthesis of Au NPs 26
8. Adapted UV-induced Borowskaja-Turkevich-Frens synthesis of Au NPs with ethanol 30
9. Surfactant-free BH_4-mediated synthesis of Au NPs 33
10. Adapted surfactant-free BH_4-mediated synthesis of Au NPs with added ethanol 39
11. Surfactant-free synthesis of Au NPs in alkaline water-ethanol mixtures 41
12. Surfactant-free synthesis of Au NPs in alkaline water-ethanol mixtures with lower grade chemicals 44
13. Surfactant-free synthesis of Au NPs in alkaline water-ethanol mixtures with citrate additives 48
14. Surfactant-free synthesis of Au NPs in alkaline water-ethanol mixtures with various Li-, Na-, K- based chemicals 56
15. Surfactant-free synthesis of Au NPs towards higher $HAuCl_4$ concentrations 59
16. Electron microscopy 62
17. Other possible cation effect(s) 65

References 66

1. Overview of literature on citrate-based syntheses of gold nanoparticles

Gold (Au) nanoparticles (NPs) obtained by citrate-mediated syntheses (often referred to as *Turkevich-Frens* method,[1] but that should maybe more accurately be referred to as *Borowskaja-Turkevich-Frens* method, as debated elsewhere[2,3]) are relatively simply obtained by mixing a gold precursor, such as $HAuCl_4$, and sodium citrate (NaCt) in water, to lead under various conditions to size controlled NPs, typically in the range 10-100 nm. A detailed discussion on the formation mechanism and stabilization can be found elsewhere.[1] The synthesis is typically induced at relatively high temperature, e.g. close to the boiling point of water,[1,4] but can also be induced at room temperature by lights of different wavelengths, where lower wavelengths favor a faster formation.[5,6] In most cases, size control is achieved by controlling the NaCt/Au molar ratio, where too high or too low ratios are reported to lead to larger NPs.[1,7] The NaCt/Au ration controls the pH which is a key factor to control for the synthesis due to the complex chemical equilibria between the different forms of the Au complex used and the citrate species.[1,8] The NPs are relatively stable over time.[9]

Over the years, an increasing control and refinement of the synthesis have been achieved to improve NP monodispersity, stability and/or the scalability of the synthesis.[10-15] The importance of various parameters that influence the outcome of the synthesis has been studied and a succinct account of some important parameters are reported in **Table S1**. Examples of experimental parameters are the order of addition of the chemicals (*direct method*: adding the citrate last; *inverse method*: adding the gold last),[12,16] the ageing of the chemical (such as the gold precursor stock solution)[17] or various additives to improve the size and size distribution, such as citric acid,[13] NaOH[18] tannic acid,[19] or EDTA.[13] Recently, flow systems were used to optimize the size of the NPs towards sizes less than 5 nm.[14] These reports helped to clarify the roles of the different chemicals. It has been established that citrate plays the multiple roles of reducing agent, stabilizing agent, and pH buffer.[1]

Numerous reports focus on using precursor concentration in the range 0.1-1.0 mM.[4,20] For instance 2.5 mM is considered a *high* concentration.[18,21] This relatively low maximum concentration is required to ensure the formation of stable NPs.[11] However, this pre-requisite severely limits the use of citrate-based materials at large scale, although the resulting NPs are intensively used for academic proof-of-concept, research and development and various applications. Experiments successfully performed at higher concentrations would be beneficial for those area of use and/or to optimize signal-to-noise ratio in a range of experimental setups.

Table S1. Condensed overview of selected studies on Borowskaja-Turkevich-Frens inspired citrate-based syntheses of Au NPs.

Ref.	Date	Highlight
2	1934	Seminal work - Feasibility
10	1951	
11	2006	Improved control over the NP size and room temperature synthesis (NaCt)
12	2011	Influence of the order of addition of the chemicals (NaCt)
13	2014	Importance of pH control (NaCt)
22	2016	BSc thesis touching upon differences using LiCt, NaCt, KCt
14	2018	Flow synthesis to achieve smaller size NPs (NaCt)
**This work**	2025	Unified study on the influence of cations comparing LiCt, NaCt, KCt<br>Stabilization decreases: $Li^+ > Na^+ > K^+$

In the majority, if not all reports to date, NaCt is preferred, see **Table S1** but also **Figure S1**. Using *Google* search engine or *Web of Science* (WOS) to investigate the potential literature using as keywords ‘lithium citrate’ or ‘potassium citrate’ and ‘gold nanoparticles’ or ‘gold nanomaterials’ does not return any relevant manuscript (as of Spring 2025). For instance, ‘lithium citrate’ and ‘gold nanoparticles’ return only 11 citations in WOS. The ‘lithium’ refers mainly to alternative lithium based chemicals or concepts such as lithium batteries. ‘Potassium citrate’ and ‘gold nanoparticles’ returns only 33 citations in WOS. An analysis shows that the NPs in those publications were prepared with sodium citrate and potassium-based chemicals (e.g. KCl, KOH, $KAuCl_4$, etc.) were used with the resulting NPs. As a comparison, ‘sodium citrate’ and ‘gold nanoparticles’ returns 897 publications. As a further comparison the search ‘gold nanoparticles citrate’ returns more than 4 100 publications.

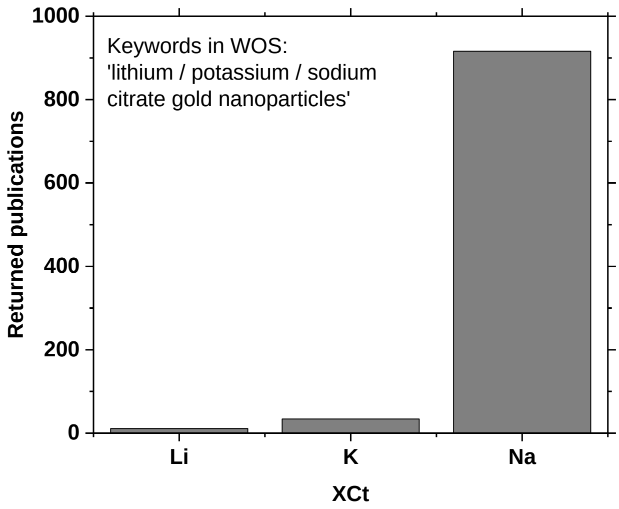

Figure S1. Number of publications returned for a search on Web of Science (WOS) for keywords ‘lithium citrate gold nanoparticles’, ‘potassium citrate gold nanoparticles’ or ‘sodium citrate gold nanoparticles’, as indicated (last updated 21/05/2025).

One work actually refers to the synthesis of Ag NPs using KCt.[23] Others relate to the use of KCt for gold-based materials.[24] We could identify one work comparing the effect of LiCt vs. KCt but for pre-formed Ag NPs obtained using $NaBH_4$.[25] There is therefore no work to the best of our knowledge that contemplates the use, therefore the differences, and thus the possible benefits of LiCt, NaCt or KCt for the synthesis of the Au NPs.

The only relevant literature returned towards a comparison of the effects of LiCt, NaCt of KCt in a same study is a BSc thesis.[22], that, to the best of our knowledge, did not lead to follow up publications. In this work, an effect of the counter ions was investigated but the focus was on seed-mediated growth. The conclusions brought forward aligns with the driving hypothesis and results of the present work. It was concluded in that thesis that LiCt prevents the *agglomeration* of the NPs during the NP growth.

2. Overview of literature on borohydride-based syntheses of gold nanoparticles

In the so-called Brust-Shiffrin approach,[26] the synthesis is performed at room temperature in a few minutes, provided freshly prepared stock solutions of XBH_4 are used. The borohydride is a strong reducing agent. The formation mechanism and stabilization is detailed elsewehere.[27] An account of some literature around this method is proposed in **Table S2**. Typically organic solvents and thiols are used to stabilize the NPs but the borohydride synthesis can also be performed in water without the need for other chemicals, although the surfactant-free and thiol-free colloidal dispersions are reported to suffer from relatively poor stability over time.[28]

Table S2. Condensed overview of selected studies on Brust-Schiffrin-inspired borohydride based syntheses of Au NPs.

Ref.	Date	Highlight
29	1994	Seminal work - Feasibility
30	2005	Example of use of $LiBH_4$
31	2010	Example of use of KBH_4
28	2014	*Stabilizer-free* version of the synthesis using $NaBH_4$
32	2014	*Stabilizer-free* version of the synthesis using $LiBH_4$
**This work**	2025	*Stabilizer-free* version of the synthesis<br>Unified study of the<br>influence of cations comparing $LiBH_4$, $NaBH_4$, KBH_4<br>Stabilization decreases: $Li^+ > Na^+ > K^+$

In the majority of reports to date, sodium borohydride ($NaBH_4$) is preferred, see **Figure S2**. Using *Google* search engine or *WOS* to investigate the potential literature using as keywords 'lithium borohydride' or 'potassium borohydride' and 'gold nanoparticles' or 'gold nanomaterials', does not return any relevant manuscript (as of Spring 2025). For instance, 'lithium borohydride' and 'gold nanoparticles' returns only 11 citations in WOS.[30,32] 'Potassium borohydride' and 'gold nanoparticles' returns only 37 citations in WOS. As a comparison, 'sodium borohydride' and 'gold nanoparticles' returns 1366 publications. In most case the lithium and potassium keywords refer to other chemicals than the borohydrides although some reports do refer to the use of $LiBH_4$,[30,32] or KBH_4,[31] as reducing agents.

It seems that $LiBH_4$, $NaBH_4$ or KBH_4 were only considered independently in previous reports: there is no unified study and therefore no direct comparison of the effect(s) of one reducing agent compared to another.

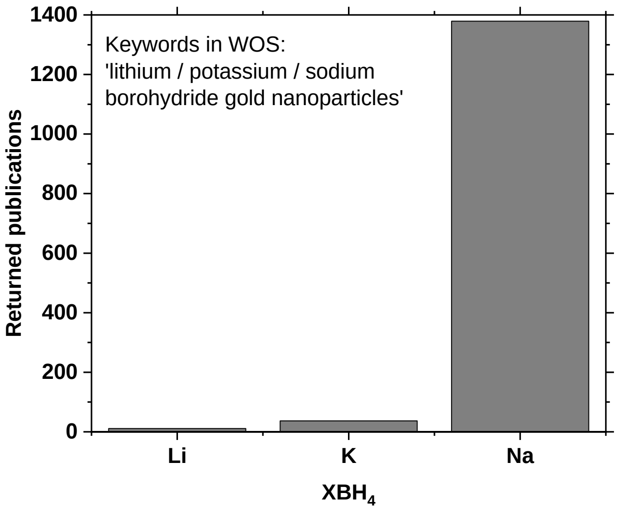

Figure S2. Number of publications returned for a search on Web of Science (WOS) for keywords ‘lithium borohydride gold nanoparticles’, ‘potassium borohydride gold nanoparticles’ or ‘sodium borohydride gold nanoparticles’, as indicated (last updated 21/05/2025).

3. Overview of literature on mono-alcohol-based syntheses of gold nanoparticles in alkaline aqueous systems

A range of Au NP syntheses can be performed in aqueous media using polyols, such as glycerol[33] or ethylene glycol.[34] Under alkaline conditions, alkoxides formed from the alcohol contribute to the reduction of the precursor at room temperature.[33] Interestingly, the reaction does not require extra stabilizing agents or surfactants.[35,36] The stabilization has been attributed to the high viscosity of the polyols. However, recent results using mono-alcohols such as ethanol or methanol show that so-called *surfactant-free* colloidal Au NPs[37] stable for years can easily be obtained at room temperature.[36,38,39] See an account and definition of *surfactant-free* colloidal NPs in previous literature.[37,40] An account of surfactant-free syntheses of Au NPs in alkaline water-ethanol mixtures is proposed in **Table S3**.

Table S3. Overview of selected studies on surfactant-free mono-alcohol mediated syntheses of Au NPs in aqueous alkaline media.

Ref.	Date	Highlight
41	2013	Probably first report. Poor control over NP size.
38	2023	Controlled ethanol- and methanol- mediated syntheses
42	2023	Importance of storage of stock solutions
43	2023	Influence of water and alcohol grade
44	2025	Importance of controlled light environment
45	2025	Effect of inducing the synthesis in different ways
36	2025	No benefits of using additives (including NaCt)
**This work**	2025	Hybrid syntheses with citrate-based and BH_4-based chemicals<br>Stabilization decreases: $Li^+ > Na^+ > K^+$

The synthesis is best performed with LiOH or NaOH while KOH lead to less reproducible results.[38,44] The addition of surfactants / additives, including NaCt, to the otherwise surfactant-free approach does not lead to any benefits.[36,38] Below we explore hybrid syntheses between this otherwise surfactant-free approach to illustrate the benefits of Li^+ cations over Na^+ and K^+ in the formation and stabilization of Au NPs.

4. Overview of literature on cations effect(s) in the syntheses of gold nanoparticles

A range of work in the literature points towards a possible effects of cations for various NP formation and stabilization,[46,47] including Ag.[25] We demonstrated ourselves the importance of alkali cations in Ir NP formation,[48] and Pt NP formation and stabilization.[47] There is therefore awareness in the literature on the possible importance of counter-cations for NP stabilization.[46,49] An effect of the cation is expected due for instance to various metal-alkali interactions,[50] impacting for instance catalysis.[51] However while anions (e.g. halides) have been widely considered to interact with Au surfaces[52] and Au complexes,[51] and possibly shape Au NPs,[53] there is overall far less consideration for the counter ion (cation) in the stabilization and formation of Au NPs.[54]

Examples of previous work pointing towards possible, and therefore expected, effects of cations on Au NP properties are comparison of the use of Cs^+ and Li^+ with DNA protected Au NPs, where Li^+ is more effective in reducing the charge repulsion among DNA.[55] Other work stresses the role of cations in the halide-induced Au NP aggregation and fusion and the ion-exchange processes on Au NP surfaces due to the cooperative and competitive cation and anion binding to Au NPs.[56] The study was performed with model electrolytes including 1,3-bis(3′-butylimidazolium)benzene dihalide salts ((BBIB)X_2), where X = Cl^-, Br^-, I^-), 1-allyl-3-methylimidazolium halide salts ((AM)X, where X = Cl^-, Br^-, I^-) and inorganic salts including alkali metal electrolytes (AME) such as KX (X = Cl^-, Br^-, I^-), Na_2S, and $AgNO_3$. Alkali cations from LiOH, NaOH or KOH also play a role to explain the different results obtained for the cathodic corrosion of Au electrodes.[57]

Given the wide use of NaCt and $NaBH_4$ for the syntheses of Au NPs, the scarcity of reports on the opportunities possibly stemming from the alternative use of LiCt, KCt, $LiBH_4$ and KBH_4 is rather surprising, see **sections 1 and 2**. The possible factors that might account for the preferred use of Na-based chemicals are (i) the more expensive price of Li-based chemicals, (ii) the more complicated process to obtain commercially available Li- and K-based chemicals (e.g. it took several months to deliver KBH_4). Furthermore, (iii) it is worth stressing that the use of Li-based chemicals comes with extra complications (e.g. higher risk for flames when preparing stock solutions of $LiBH_4$, i.e. more hazard).

5. Materials and methods

5.1. Chemicals

Extra care was taken not to use metallic spatula but to prefer disposable plastic spatula to handle all the solid chemicals. All chemicals were used as received: high purity water (H_2O, mQ, Milli-Q, Millipore, resistivity >18.2 MΩ·cm); de-ionised water (DI, 0.5-1.0 µS/cm, 1-2 MΩ•cm); ethanol (absolute, ≥99.8%, AnalaR NORMAPUR® ACS, Reag. Ph. Eur. analyse reagens, VWR); ethanol ($E_{70\%}$, Ethanol 70% (v/v), TechniSolv®, VWR), chloroauric acid trihydrate ($HAuCl_4{\cdot}3H_2O$, ACS Grade, MP Biomedical or 99.9%, Sigma Aldrich, or 99%, BLD Pharmatech); lithium citrate tribasic tetrahydrate (LiCt, ≥99.5%, BioUltra, Sigma Aldrich); sodium citrate tribasic dihydrate (NaCt, ≥99.5%, BioUltra, Sigma Aldrich); potassium citrate tribasic monohydrate (KCt, ≥99.0%, purum p.a., Sigma Aldrich); lithium borohydride ($LiBH_4$, 95%, Thermo Scientific Chemicals); sodium borohydride ($NaBH_4$, 99%, Sigma Aldrich); potassium borohydride (KBH_4, 99.9%, Sigma Aldrich); LiOH (≥98.0%, ACS reagent, Sigma Aldrich); NaOH (≥98%, reagent grade, Sigma Aldrich); KOH (≥85%, ACS reagent, Sigma Aldrich, pellets); HCl (puriss. ACS reagent, reag. ISO, Reag. Ph. Eur. fuming, ≥ 37%, Sigma Aldrich); HNO3 (puriss ≥ 65%, Sigma Aldrich).

Note. Sodium dodecylsulfate (NaDS) is a common additive in various syntheses of NPs including Au NPs.[36] Although lithium dodecylsulfate (LiDS) is commercially available, we could not find any supplier for potassium dodecylsulfate (KDS). Our attempts to compare LiDS (≥98.5%, Sigma Aldrich) and NaDS (ReagentPlus®, ≥98.5%, Sigma-Aldrich) when developing hybrid syntheses reported previously[36] did not show any clear advantage of LiDS over NaDS, in agreement with the relatively minimal effect of NaDS in the first place on the synthesis.[36] Those chemicals were however used for data presented in **section 14**.

5.2. Synthesis

5.2.1. *Green* and *Sustainable* practices

In the following, the preference to use relatively simple syntheses, performed at low temperatures, in low volumes, using disposable cuvettes as reactors, is to comply with the principles of *Green* and *Sustainable* practices in the laboratory,[58] and to allow a higher throughput. Simpler syntheses requiring fewer chemicals have the potential to lead to overall greener processes, lower temperatures lead to low energy processes, lower volumes minimize waste and/or allow investigating higher concentrations of chemicals without wasting too much resources (as opposed to strategies requiring larger volumes), lower volumes also minimize the footprint of the experimental setup. Using disposable cuvettes can be a drawback because it generates plastic waste, but it alleviates from the cleaning of glassware with corrosive and harmful *aqua regia.* In the present work, only a limited amount of *aqua regia* was used to clean the small size magnets. Note that the use of plastic-based containers can limit the temperature range used. Finally, the kinetic studies proposed will be more challenging to perform if the syntheses were performed at elevated temperatures (aliquots from a larger volume of solution would need to be taken overtime whereas the present approach allows continuous monitoring). For all these reasons, a preference was given here to UV-induced and/or room temperature induced syntheses to best illustrate the effect of the counter-cations. Nevertheless, experiments were also performed at higher temperature in case of citrate-mediated syntheses as detailed in section **S7**.

5.2.2. General considerations

All solutions (stock and syntheses) were prepared using mQ water, unless otherwise specified. The concentrations of the stock solutions are detailed below and the concentration was typically 50 mM for $HAuCl_4$. The syntheses were performed in disposable 1 cm wide square UV-vis cuvettes made of polystyrene for a total volume of 2 mL, unless otherwise specified. This low volume was preferred for two reasons. First, a small volume minimizes waste for the large screening of experimental conditions performed here. Second, it enables us to work at lower cost at the relatively high concentration of 0.5 mM of $HAuCl_4$, which ultimately is relevant to scale up the synthesis.[38,44]

Magnetic stirrers were used to mix the solution during reaction, unless otherwise specified. Magnets used were cleaned with *aqua regia* (4:1, v:v, HCl:HNO_3). ***Beware that aqua regia is extremely corrosive and needs to be disposed of carefully following the regulations enforced at the working place.*** Once a magnet was placed in the cuvette, the cuvette and magnets were then de-dusted with a gentle flow of compressed air. The required chemicals (as detailed in the next sections) were then added, stirring performed (typically at 500 rpm). The samples were capped right after adding the $HAuCl_4$, unless otherwise specified. In our experience, capping or not capping the cuvette used as reactor does not change the outcome of the synthesis for ambient light induced synthesis but capping the cuvette used as reactor minimizes risks of spillover. $HAuCl_4$ was typically added last, unless otherwise specified.

The reaction was left to proceed under controlled light environment (e.g. in a photo-box, Puluz LED portable Photo Studio, PU5060EU, 60 cm x 60 cm x 60 cm, 60 W, unless otherwise specified) for 2 hours. The samples were then left to stand overnight at ambient light and temperature. The magnets were then removed the day after synthesis before further use and/or characterization. Unless otherwise specified, the samples were further kept at room

temperature in a drawer, alternatively in a fridge in some specific cases, as indicated. See more details on the general set up in [44] and [43].

Note. The outcome of nanomaterial syntheses can be challenging to reproduce, for instance due to small variations of the concentrations of stock solutions, e.g. of $HAuCl_4$ or bases.[45] In all cases below and as much as possible for each section, the comparison Li^+ vs. Na^+ vs. K^+ was made with the same stock solutions of gold precursor, bases and additives to study as much as possible the effect of the cation only. It is also worth pointing out that the consistent effects reported in the present report were observed and documented by different experimentalists of different levels of expertise.

5.2.3. Borowskaja-Turkevich-Frens synthesis of Au NPs

The synthesis followed the general procedures from the literature.[1] Stock solutions of LiCt, NaCt, KCt between 25 and 750 mM were used (prepared in mQ water). These stock solutions were used and diluted with mQ water to obtain the desired concentrations for the experiments. Stirring was then started and $HAuCl_4$ from a stock solution at 50 mM was then added last under stirring to initiate the synthesis. The amount of citrate and $HAuCl_4$ were adjusted by tuning the volume of stock solution used in order to obtain the desired final amount of citrate and gold, as indicated.

UV-vis induced synthesis. To minimize the volume of solution used and allow a higher throughput, the synthesis was induced by a 365 nm light (OFK-8000 OptimaxTM Multi-LiteTM inspection kit (Spectroline), see [44] for details) where typically 3 to 9 samples at a time were placed under the same UV-lamp in a Becher to avoid the tilting and spillover of the samples. The $HAuCl_4$ was then added last under stirring and under controlled UV light. The open samples (not capped) were irradiated from the top for 2 hours before the light was turned off and the samples capped. The Becher containing the samples and the part of the lamp shining light on the sample was covered in aluminum during the experiments. The part of the lamp shining light was situated ca. 4 cm from the top of the UV-cuvettes.

Heat induced synthesis. Heat induced experiments were also performed to confirm the trends observed. Reactions were carried out in glassware following the procedure above. The glassware was chose to have a ''glass test tube shape'', i.e. relatively high walls to minimize evaporation of the small total volume of water used that was 2 mL. In such glassware, mQ was added, followed by XCt. To minimize contamination and water evaporation, a plastic cap was loosely fitted onto the test tube. The test tube was placed in a hot mQ water bath. As the water bath reached a stable temperature of 90 ± 2 °C, $HAuCl_4$ was added. A subsequent color change—typically black, purple, or blue—was observed, indicating the progression of the reaction. The reaction was considered complete once a ruby red coloration was observed, which generally occurred within approximately 6 to 10 minutes, depending on reagent concentration. Higher concentrations (see results presented) resulted in significantly reduced reaction times, with red coloration observed within 4 minutes to as little as 1 minute and 30 seconds. Following the completion of the reaction and cooling to ambient temperature, the solution was transferred into a cuvette. UV-Visible spectroscopy measurements were conducted the following day.

5.2.4. Adapted Borowskaja-Turkevich-Frens synthesis of Au NPs with ethanol

To illustrate further the influence of cations, a modified recipe to the protocol detailed in **section 5.2.3.** was used where various amounts of ethanol were added to the reaction mixture (before adding $HAuCl_4$ still last). The amount of ethanol used and indicated is expressed before taking into account volume contraction.[38]

Adding ethanol (using ethanol in various amounts but keeping the overall volume and concentrations of the other chemicals the same) provides a surplus of reducing agent that typically leads to larger and less stable Au NP colloids.

5.2.5. Surfactant-free BH_4-mediated synthesis of Au NPs

To minimize the use of chemicals, a surfactant-free and thiol-free syntheses of Au NPs in water reported by Astruc and co-workers was used.[59] The stock solutions consisted of $HAuCl_4$ at 50 mM and $LiBH_4$, $NaBH_4$, KBH_4 at 100 mM. The stock solution of $LiBH_4$, $NaBH_4$, KBH_4 were prepared by adding the desired mass of solid powder in successive small amounts of $LiBH_4$, $NaBH_4$, KBH_4 to a glass container already containing the desired amount of water. **Note that $LiBH_4$, $NaBH_4$, KBH_4 can react upon contact to water and lead to flames.** Adding the $LiBH_4$, $NaBH_4$, KBH_4 to water (rather than adding water to a powder of $LiBH_4$, $NaBH_4$, KBH_4) minimized the risk of flames. We did not observed any flame is the borohydride powder was added progressively to water. The probability to observe such flames decreases with $LiBH_4$ > $NaBH_4$ > KBH_4. **We recommend the use of rather large glass container to potentially contain the flames.**

The synthesis of the Au NPs was initiated by adding the desired amount of freshly prepared borohydride solution (less than 15 minutes old) to a mixture of water and $HAuCl_4$ under stirring at room temperature. The volume of borohydride solution was chosen to control the final molar ratio of XBH_4/Au as indicated while the final concentration of $HAuCl_4$ was 0.5 mM (from a stock solution at 50 mM). For those experiments, the final volume was 3 mL. The reaction is almost instantaneous and is probably not sensitive to lights but was nevertheless performed in a controlled light environment (photo-box).

5.2.6. Adapted surfactant-free BH_4-mediated synthesis of Au NPs with ethanol

To illustrate further the influence of cations, a modified recipe to the protocol detailed in **section 5.2.5.** was used where various amounts of ethanol were added to the reaction mixture (before adding the borohydride solution still last). The amount of ethanol used and indicated is expressed before taking into account volume contraction.[38]

Adding ethanol (using ethanol in various amounts but keeping the overall volume and concentrations of the other chemicals the same) provides a surplus of reducing agent that typically leads to larger and less stable Au NP colloids.

5.2.7. Surfactant-free synthesis of Au NPs in alkaline water-ethanol mixtures

Stable *surfactant-free*[37] Au NPs can be obtained at room temperature by adding $HAuCl_4$ to an alkaline mixture of water-ethanol.[38] It was previously noticed that more stable Au NPs tend to be obtained when LiOH is used compared to when NaOH is used and even less stable are Au NPs obtained using KOH.[38,44] The synthesis is sensitive to the purity of the water used, as most Au nanomaterials syntheses are.[60,61] The use of lower grade water (DI) leads to larger and less stable Au NPs as the content of lower grade water increases in mQ water.[43,62]

The syntheses were performed in controlled light environment (photo-box) and the $HAuCl_4$ (from a stock solution at 50 mM) added last under stirring to a solution of the mQ, the base and ethanol. The chemicals were added in the order mQ, base (from stock solutions at 50 mM kept in plastic containers), and ethanol. The final concentration was typically 0.5 mM $HAuCl_4$

and 2 mM of base. Different amounts of water and base (LiOH, NaOH, KOH) from stock solutions at 50 mM were used, as indicated.

High purity chemicals. The synthesis proceeds well with absolute ethanol and mQ water. Such chemicals were preferred.[43,44,62]

Lower purity chemicals. We showed that using lower grade ethanol was suitable to obtain Au NPs.[43] We showed that using deionized water in small amount could lead to size control over the surfactant-free Au NPs and the NP size increases as the amount of DI water increases when NaOH is used.[62] As the amount of DI water increases, the NPs are also less stable. We here investigated the effect of using different bases, i.e. LiOH and KOH. To assess the potential benefits of Li^+, Na^+ or K^+ on this synthesis, different reaction mixtures with a final ethanol content of 20 v.% (before volume contraction) but with different amounts of DI water, as indicated, were used. The chemicals were mixed in the order mQ, DI water, ethanol and $HAuCl_4$.

5.2.8. Surfactant-free synthesis of Au NPs in alkaline water-ethanol mixtures with citrate additives

Adding *stabilizers* / *polymers* / *capping agents* / *ligands* / *protective agents* to the otherwise surfactant-free synthesis of Au NPs detailed in **section 5.2.7.** (i.e. a mixture of 0.5 mM $HAuCl_4$ and 2 mM NaOH in 20 v.% ethanol and 80 v.% mQ) does not lead to any noticeable benefits on the size control or stability of the colloids.[36] On the contrary, the use of NaCt actually leads to larger Au NPs probably due to an excess of reducing agent.[36]

To evaluate the relative effect of LiCt, NaCt, KCt, experiments similar to those performed as described in **section 5.2.7.** were carried out. The only difference is that LiCt, NaCt or KCt was used so that the final XCt/Au molar ratio was changed, as indicated, using different volumes of stock solutions of XCt at 50 mM. $HAuCl_4$ was still added last under stirring. The final concentration of $HAuCl_4$ was 0.5 mM or 0.2 mM (from a stock solution at 50 mM) and the final concentration of base was 2 mM or 0.8 mM (Base/Au molar ratio of 4), in 20 v.% ethanol and 80 v.% mQ.

The samples obtained with NaCt are discussed elsewhere.[36]

5.2.9. Surfactant-free synthesis of Au NPs towards higher $HAuCl_4$ concentrations

Those experiments were performed at the MAX IV synchrotron facilities, Lund, Sweden, during a synchrotron beamtime (ID 20240084) at the DanMAX beamline. The syntheses were performed without stirring in 15 mL centrifuge tubes (polypropylene) with the same chemicals as described above, except for the ethanol (anhydrous, 99.9%, KiiltoClean) and NaOH (97%, Sigma Aldrich). The total volume was 3 or 5 mL, as indicated. The stock solutions of XOH and XCt were at 50 mM, the stock solution of $HAuCl_4$ was at 20 mM. In this case, only X = Li and X = Na were considered given the challenges to use X = K detailed along the present manuscript. The chemicals were mixed to obtain the desired ratio and $HAuCl_4$ concentrations, as indicated. No stirring was performed but the sample manually shook after adding the $HAuCl_4$ and closing the centrifuge tube with the dedicated cap. The syntheses were performed in a photo-box (Puluz LED portable Photo Studio, PU5040EU, 40 cm x 40 cm x 40 cm, 30 W) for 2 hours. The samples were left on the bench at ambient light and temperature afterwards.

5.2.10. Comment on reproducibility

Challenges in reproducibility are inherent to the complexity of nanomaterial science and the synthesis of Au NPs is not an exception.[61,63] The trends observed and discussed were consistent across several measurements and using different stock solutions of $HAuCl_4$, LiCt, NaCt or KCt, or $LiBH_4$, $NaBH_4$, KBH_4, LiOH, NaOH or KOH prepared on different days. The same batch of stock of chemicals were used to prepare those stock solutions, newly acquired and only used for this project and previous publications.[36,44,45,62] The solutions are therefore expected to be clean from any impurities other than those inherent to the preparation of the chemicals themselves (as documented by the supplier).[63]

5.3. Characterization

5.3.1. General consideration

It is common to propose multiple characterization technique of NPs.[64] However, a sustainable conduct of research should avoid wasting time and ressources.[58] Due to the large number of samples considered, a relative high throughput method was preferred. UV-vis is a very informative and well-established characterization technique for Au NPs. UV-vis is compatible with the relatively low volumes of solutions preferred here (see **sections 5.2.1** and **5.2.2.**). Only selected samples were characterized by more advanced techniques such as scanning transmission electron microscopy (STEM) and X-ray total scattering (TS, F(Q)) with pair distribution function (PDF, G(r)) analysis. Interested readers will find elsewhere a vast literature on further characterization of Au NPs obtained by citrate-based and borohydride-based as well as mono-alcohol mediated syntheses, including methods such as SAXS,[65] XRD,[38] or XPS.[38] However, the focus of this article is not *per se* on the structural characterization nor surface functionalization of the Au NPs, largely documented elsewhere, but rather on the relative influence of cations Li^+, Na^+ and K^+ on syntheses of Au NPs that are considered well-established.

5.3.2. UV-vis

Metrics. UV-vis was used as a well-established method to retrieve information on the Au NPs due to their plasmonic properties.[15] Information on the size can be retrieved from the wavelength at the surface plasmon resonance (spr), λ_{spr}, or the ratio of the absorbance values recorded at the spr and at 450 nm, A_{spr}/A_{450}. The Au NPs tend to be smaller as the λ_{spr} or A_{spr}/A_{450} values decrease.[66] Higher λ_{spr} values can also indicate non-spherical NPs. Information on the stability of the NPs is given by the ratio of the absorbance recorded at 650 nm and at the spr, A_{650}/A_{spr}, or the ratio of the absorbance recorded at 380 and 800 nm, A_{380}/A_{800}. The colloidal dispersions tend to be more stable as the A_{650}/A_{spr} ratio decreases[67] or as the A_{380}/A_{800} ratio increases.[68] Finally, the relative intensity at 400 nm gives an indication on the relative yield.[69] In our experience over now more than 1000 samples studied, there is, as expected from theory, a good correlation between those metrics and the size and/or stability of the Au NPs.[38,44,62]

A summary of the various metrics is proposed in **Figure S3**. Note that the trends reported are theoretically valid only for a specific size range of Au NPs since the UV-vis spectra of gold NPs result from a complex interplay between the Au NP size, shape, concentrations, nature of the surrounding media and/or interaction between Au NPs and non-reacted precursor.[70] *Stricto sensu*, the use of different cations could lead to different UV-vis spectra for a same NPs size, in the same way that different solvent compositions could lead to different Au NP spectra for NPs with a same size. In our experience with now more than 1000 samples,[38,44,45,62,71] the λ_{spr} value is a good indicator of the NP size and provided the λ_{spr} is around 520 nm the A_{spr}/A_{450} remains also a suitable indicator of the NP size. There is also a good correlation between the A_{380}/A_{800} value and the stability of the colloidal dispersions. This is also illustrated below in **section 9**, e.g. **Figure S19**.

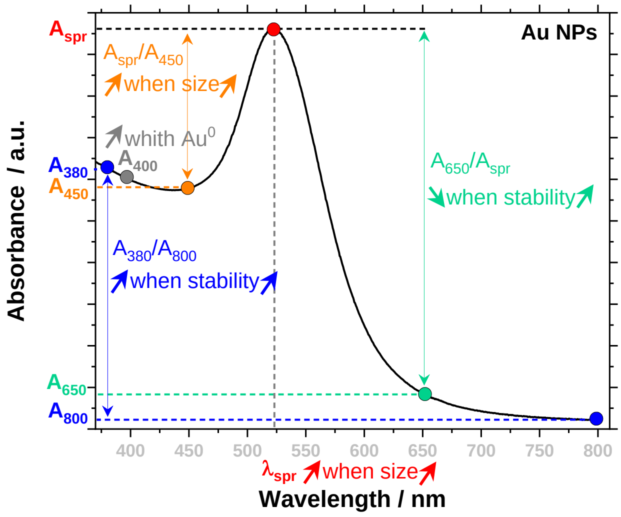

Figure S3. Illustrative UV-vis spectra of Au NPs with various relevant metrics.

General. The UV-vis measurements were performed the day after the synthesis, unless otherwise specified. A Thermo Scientific Genesys 10 s UV–vis spectrophotometer was used with measurements in the range 290–800 nm on the as-prepared colloidal dispersions in the UV–vis cuvettes used as container for the synthesis for syntheses performed with 0.2 or 0.5 mM of $HAuCl_4$, or diluted to an equivalent of 0.5 mM of Au (0.5 mM_{Au}) when higher precursor concentrations were used for the synthesis. A blank with the same composition of the samples (excluding, base, citrate or borohydride because those chemicals do not absorb in the range considered, but using the same amount of ethanol as in the sample) was used (without $HAuCl_4$ added). The error bars in the following correspond to statistical errors retrieved from at least three different syntheses for UV-vis.

Kinetics. The formation of Au NPs was examined for the synthesis by *in situ* time resolved UV-vis spectroscopy using a Go Direct SpectroVis Plus Spectrophotometer.[44,71] The baseline measurement was taken as the water and citrate solution before addition of $HAuCl_4$, while under the UV-lamp.

5.3.3. Scanning Transmission Electron Microscope (STEM)

STEM micrographs were acquired on an FEI Talos F200X operated at 200 kV and equipped with high-angle annular dark-field (HAADF) detector and a bright field detector. The colloidal dispersions prepared by the above-described syntheses were directly dropped on copper TEM girds. After solvent evaporation, the measurements were performed and size distributions were retrieved with the software ImageJ from typically at least a 100 NPs, as indicated. The error bars over diameter and sizes in the following correspond to statistical errors retrieved from typically one STEM grid for size estimation of a given experimental condition (whereas UV –vis data are average over three replicates of the same experimental condition).

More advanced characterization of the NP morphology and size distribution were examined by STEM in section **S16** below. The FEI Talos FX200 operating at an acceleration voltage of 200 kV in scanning nanobeam mode. The instrument is equipped with a Quantum Detectors MerlinEM-4R direct electron detector, which was used for the acquisition of four-dimensional STEM (4D-STEM) data. Conventional STEM data was recorded with a 70 µm condenser C2 aperture (i.e. 10.5 mrad convergence angle) at a 77 mm camera length, with the bright-field

(BF) and annular dark-field (ADF) detectors for morphology inspection, and HAADF detector for statistics. 4D-STEM data was recorded with a 20 µm condenser C2 aperture (i.e. 3mrad convergence angle) at a 98 mm camera length.

5.3.4. X-ray scattering with pair distribution function analysis

Total scattering (TS) experiments were performed at the DanMAX beamline at the MAX IV Laboratory using a Si (1 1 1) monochromator beam with an energy of 35.00 keV (λ = 0.354 Å). The incident beam was focused to approximately 0.814 mm (H) × 0.722 mm (V) FWHM at the sample position. Scattered intensities were recorded using a DECTRIS PILATUS3 X 2M CdTe detector placed 149.9 mm downstream of the sample. The sample-to-detector distance was determined based on measurements on crystalline Si (NIST SRM 640f). Each measurement, for both sample and blank, was collected for 5 min in a fused silica capillary with an 0.7 mm inner diameter and an 0.85 mm outer diameter. Here, "blank" measurements—performed without the metal precursor—were used for blank/background subtraction, while "sample" measurements—collected with the metal precursor—provided the actual NP scattering signal.

After each measurement, the two-dimensional detector images were azimuthally integrated using the MATFRAIA algorithm.[72] The resulting one-dimensional TS data were subsequently processed with PDFgetX3[73] yielding the reduced total scattering function, F(Q), and the reduced atomic pair distribution function, G(r). For this study, we used $Q_{min} = 0.5\ \text{Å}^{-1}$, $Q_{maxinst} = 18.5\ \text{Å}^{-1}$, and $Q_{max} = 15\ \text{Å}^{-1}$, with $r_{poly} = 0.9$. The PDF data were refined using a cluster-mining procedure,[74] detailed elsewhere.[75]

The structure library were built using the Atomic Simulation Environment (ASE),[76] adopting a lattice constant of 4.07 Å and scattering patterns of these finite structures were computed using the Debye scattering equation,[77,78] using DebyeCalculator.[79] Data fitting was done on F(Q) and G(r) data separately and combined on ranges of 1.2–15 Å^{-1} and 2–60 Å. We use a Q_{damp} value of 0.0274 Å^{-1} to represent instrumental broadening, determined from a crystalline Si standard (NIST SRM 640f) measured under identical conditions. Fits also accounted for scaling factors and atomic displacement parameters.

5.3.5. Centrifugation

In order to evaluate the relative stability of the colloidal Au NPs, the relative loss of intensity at 400 nm evaluated by UV-vis was used. The UV-spectra of as-prepared colloidal dispersions (2 mL) were measured. Then the dispersion was subjected to centrifugation with a VWR Galaxy Ministar microcentrifuge for 45 min at 6000 rotations per minutes (rpm), equivalent to 2000 x g. Afterwards, the UV-vis spectra of the supernatant (at least 1.5 mL) was measured. The relative stability of the solution is expressed as the ratio of A_{400} after and before centrifugation.

Controls ensure that no Au NPs were lost during the procedure: as the centrifuged supernatant (1.5 mL) is added to the least stable Au NPs (0.5 mL) and the sample homogenized by shaking, a A_{400} value similar to what was obtained before the centrifugation was recovered. We also made sure that a volume of 1.5 mL or 2 mL was above the minimal volume needed to reproducibly characterize the Au NPs (the same signal of a given Au NP dispersion is obtained when 1.5 or 2 mL of solution is used).

The same experiments were performed in **section 11** but with 1 hour centrifugation time.

6. UV-induced Borowskaja-Turkevich-Frens synthesis of Au NPs

In this section, the syntheses were performed as described in **section 5.2.3**. The syntheses were induced using a 365 nm lamp. The chemicals were mQ, XCt (X = Li, Na, K) and $HAuCl_4$, as indicated. The results show the effects of the cations on the UV-induced Borowskaja-Turkevich-Frens synthesis of Au NPs.

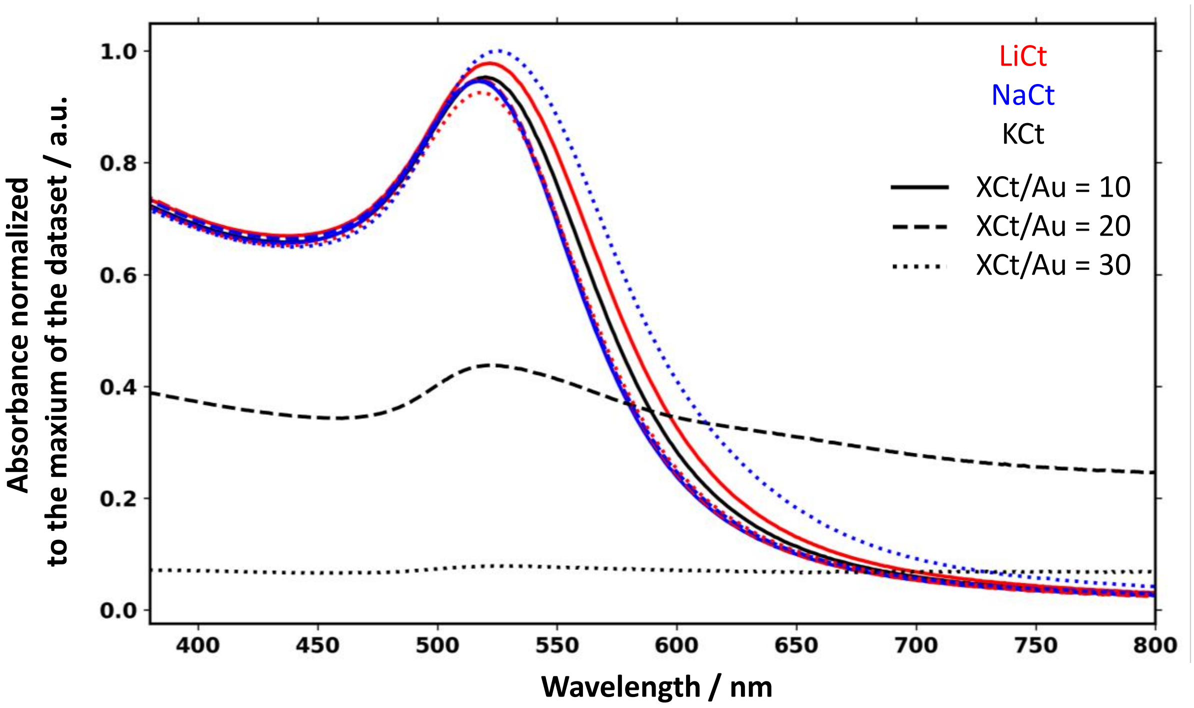

Figure S4. UV-vis spectra of Au NPs obtained using different XCt/Au molar ratios for different cations, as indicated. The spectra of LiCt/Au and NaCt/Au molar ratios of 20, together with the spectrum for LiCt/Au molar ratio of 30 are almost identical and overlap. The corresponding STEM data are given in Figure 1 of the manuscript.

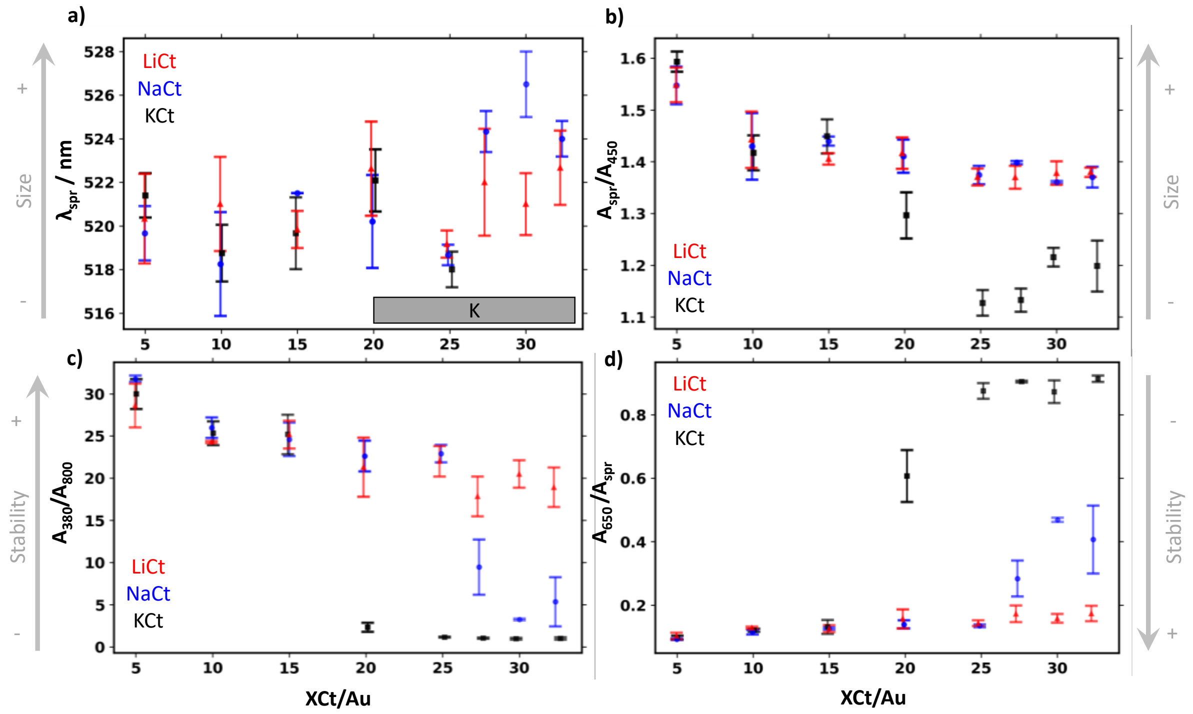

Figure S5. Various metrics retrieved from UV-vis: (a) λ_{spr}, (b) A_{spr}/A_{450}, (c) A_{380}/A_{800}, (d) A_{650}/A_{spr}. The expected evolution of the size or stability with the metrics values is added to help the reader (see also **section 5.3.2**). The NPs were prepared with 0.5 mM $HAuCl_4$ and different XCt/Au molar ratios, as indicated, and different cations, as indicated. The grey square in (a) is to stress that for samples obtained using KCt for KCt/Au molar ratios above 20, the colloids typically show poorly defined spr and values that could be retrieved are typically above 530 nm (meaning that the low A_{spr}/A_{450} values indicated in (b) do not relate to smaller size NPs). Error bars were obtained from at least 3 replicates.

There is no clear trend on the influence of the cations at lower XCt/Au molar ratios below ca. 20, considering the metrics related to size (λ_{spr} and A_{spr}/A_{450}) or stability (A_{380}/A_{800} and A_{650}/A_{spr}), see **Figure S5**. However, both size and stability metrics, see **Figure S3**, indicate that larger and less stable nanomaterials are obtained when the XCt/Au molar ratio increases and this happens at relatively lower XCt/Au molar ratios with KCt, then NaCt and ultimately LiCt.

The use of different cations influences the stability of the Au NPs and the relative stability decreases with $Li^+ > Na^+ > K^+$,

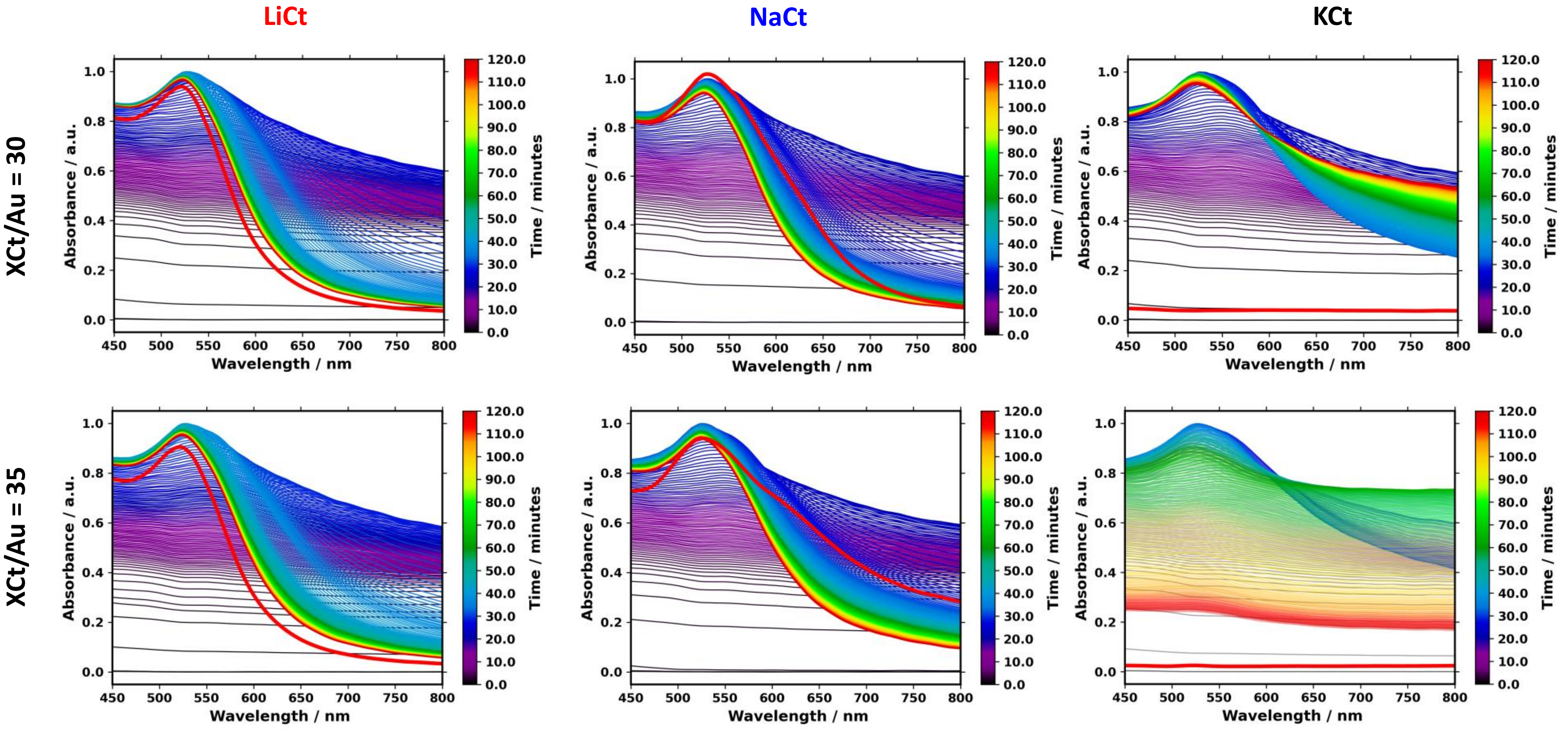

Figure S6. Time resolved UV-vis data for syntheses performed with XCt/Au mola ratios of 30 or 35 as indicated with X = Li, Na, K, as indicated. The spectrum recorded after 24 hours are indicated in bold-red (thicker spectrum). The normalization of the absorbance is made to the maximum of each dataset in each panel.

Minimal change in the spectra is observed using LiCt after 24 hours. For NaCt, the spectra recorded after 24 hours indicate larger and/or less stable NPs. For KCt the colloids are not stable (very low absorbance measured).

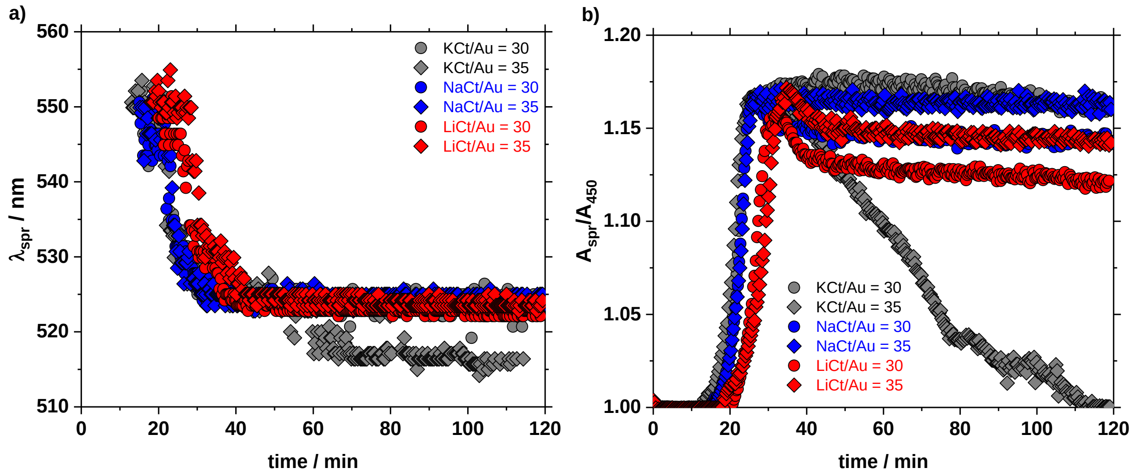

Figure S7. Time resolved metrics retrieved from UV-vis: (a) λ_{spr} and (b) A_{spr}/A_{450} as a function of time.

The decrease of the λ_{spr} and A_{spr}/A_{450} values for KCt/Au = 35 molar ratio is to be related to the poor stability of the colloids as indicated in **Figure S6**.

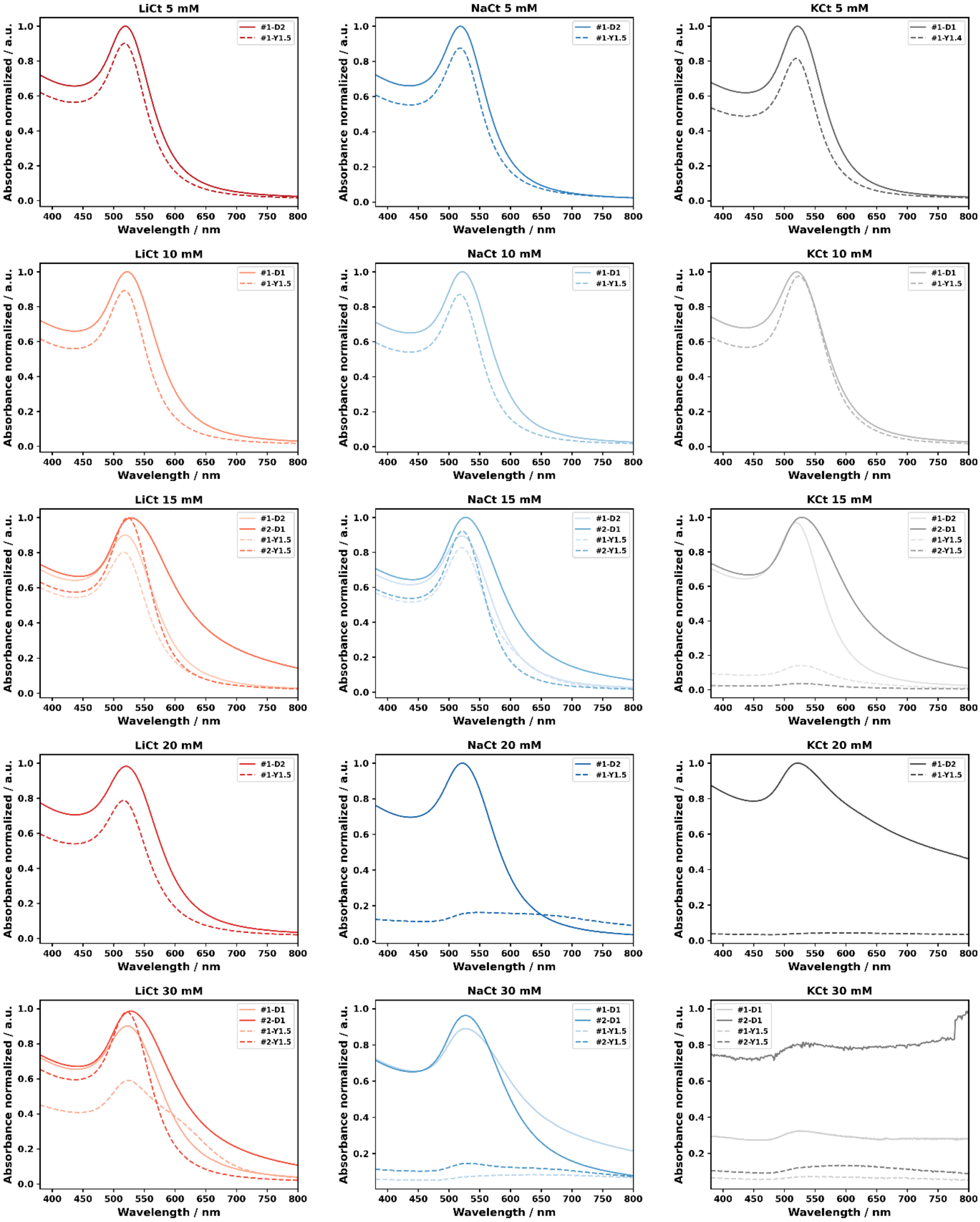

Figure S8. UV-vis spectra of colloidal Au NP dispersions obtained using different concentrations of LiCt, NaCt, or KCt, as indicated, for a UV-induced synthesis, measured a day after synthesis (D1) and a year and a half after synthesis (Y1.5). The NPs were stored at room temperature and in a drawer. Evaporation naturally happens, the volumes of the dispersions were re-adjusted to 2 mL prior to the UV-vis measurement. In all cases 0.5 mM $HAuCl_4$ was used. Duplicates are reported.

The decrease in the absorbance at 400 nm is attributed to the sedimentation of the largest Au NPs. It is rather clear that as the concentration of KCt increases, the NPs are not stable over time. This effect is also observed when NaCt is used, although slightly more stable NPs are obtained (e.g. for 15 mM of NaCt compared to 15 mM of KCt). The most stable colloids were obtained using LiCt, although there is also a trend that the NPs are less stable as the LiCt concentration increases.

The overall position of the λ_{spr} tends to be the same over time or to decrease, which is attributed to the fact that the largest NPs sediment, leaving a sample with overall smaller size NPs. The overall shape of the UV-vis spectra when the NPs are stable (minimal decrease in the absorbance at 400 nm, i.e. samples with LiCt or lower concentrations of NaCt or KCt) is overall the same.

The effect of LiCt vs NaCt vs KCt is in particular pronounced at higher XCt concentration: Only LiCt leads to stable NPs over time at higher concentration of 30 mM, and the trends that the stability over time decreases with LiCt > NaCt > KCt is particularly clear when 20 mM of XCt was used.

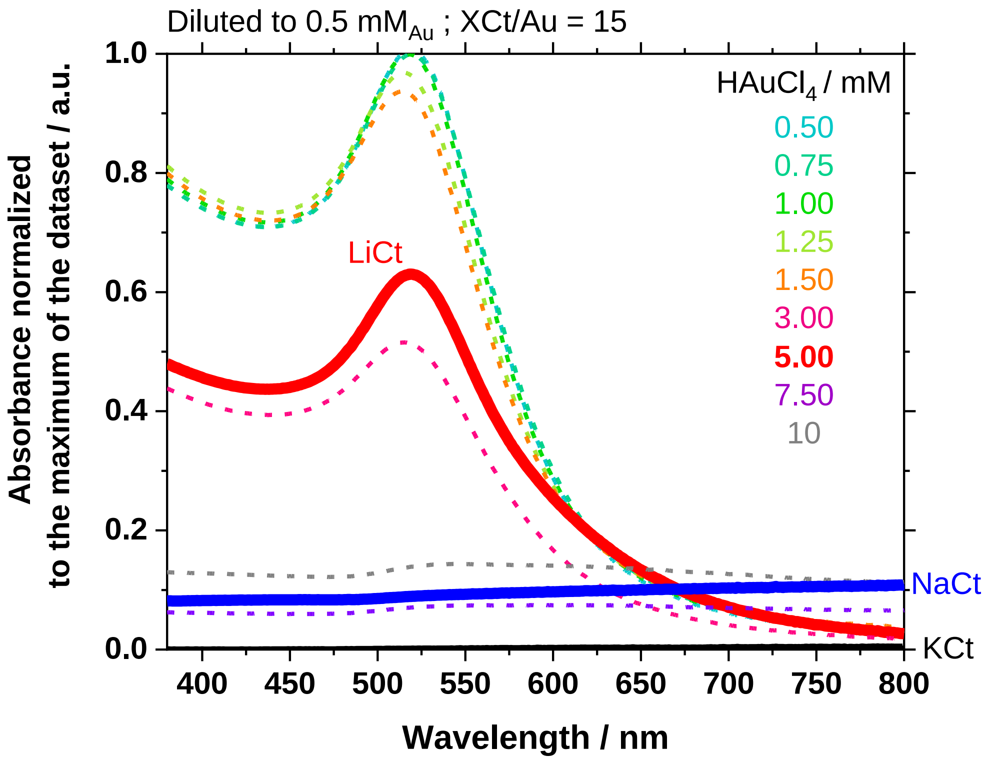

Figure S9. UV-vis spectra of Au NP colloidal dispersions obtained using different concentrations of $HAuCl_4$ for a XCt/Au molar ratio of 15. For comparison, the results obtained using 5 mM $HAuCl_4$ and LiCt, NaCt or KCt, are also reproduced here in thicker lines, as indicated. The measurements were performed on dispersions diluted to 0.5 mM$_{Au}$.

A_{400} is reflective of the yield and stability of the Au NPs. The fact that the A_{400} value decreases for higher concentrations of precursor in **Figure S9** suggests that the yield of the synthesis is however not optimal after 24 hours (compared to using lower concentrations of $HAuCl_4$, e.g. 1.5 mM). Probably the yield could be improved by, for instance, increase the amount of reducing agent (XCt) but in our experience too high concentrations of precursor *and* XCt do not lead to stable colloids. There was no incentive to optimize this yield at this stage.

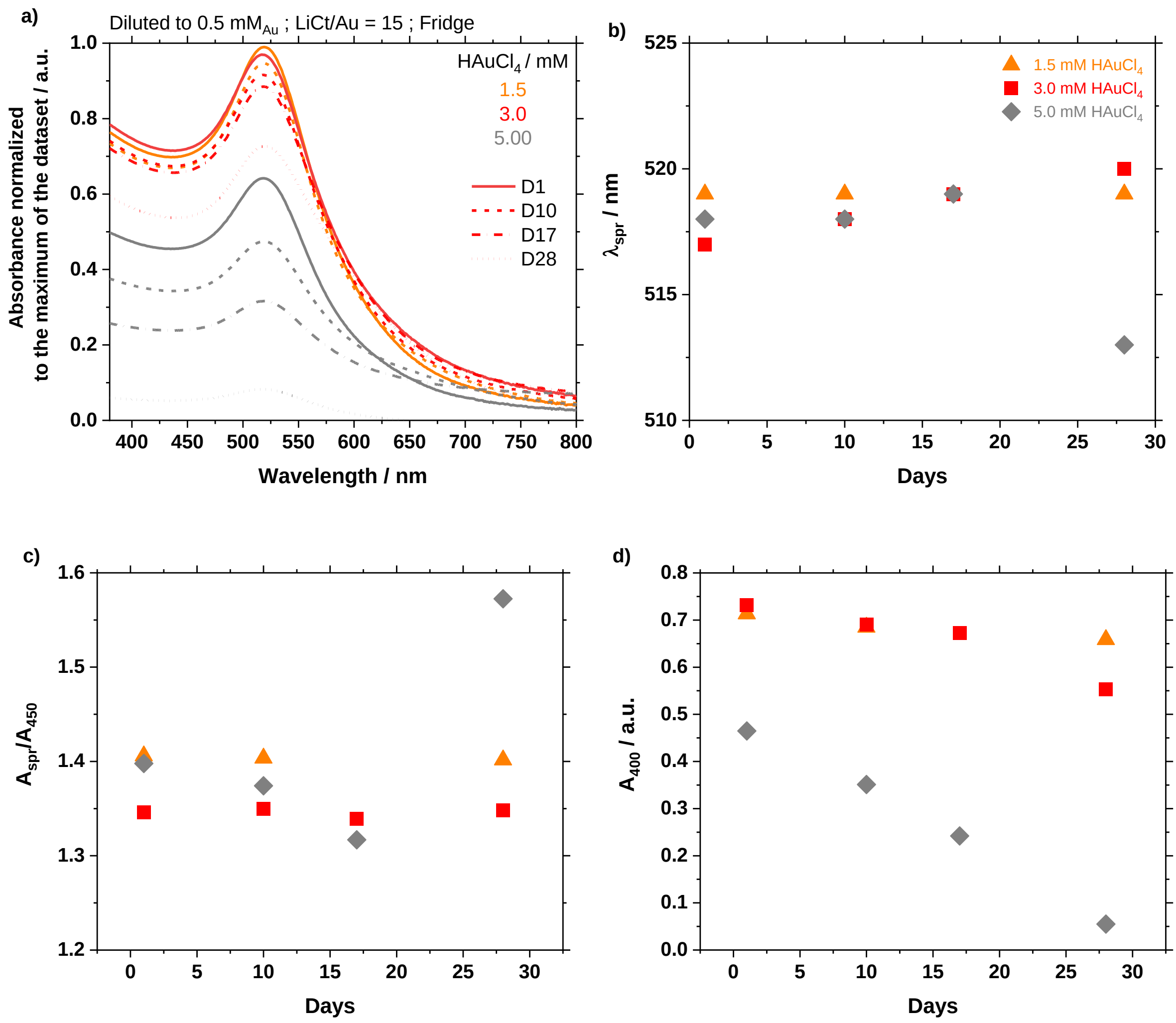

Figure S10. Au NPs obtained using LiCt at 5.0 mM of $HAuCl_4$ and with LiCt/Au=15 diluted to 1.5 or 3.0 on the day of synthesis and stored in a fridge for 1, 10, 17, and 28 days (D1, D10, D17 and D28, respectively), as indicated. (a) UV-vis spectra over time of Au NPs (diluted to 0.5 mM_{Au} on the day of measurement). (b) λ_{spr}, (c) A_{spr}/A_{450} and (d) A_{400} over time of storage.

While the synthesis is possible at relatively high concentrations of $HAuCl_4$, the stability of the Au NPs over time is limited at higher precursor concentrations, see **Figure S10**. This is in particular clear with the decrease of the A_{400} values for samples prepared at 5 mM of $HAuCl_4$ and kept at 5 mM. This effect is less pronounced for samples obtained at 5 mM but then diluted at 3 mM. Note that it is common to dilute colloidal dispersions obtained at relatively high concentrations of precursors to ensure longer term stability and this strategy can be applied here to still lead to stable colloids at relatively high concentrations (e.g. 3 mM).

7. Temperature-induced Borowskaja-Turkevich-Frens synthesis of Au NPs

In this section, the syntheses were performed as described in **section 5.2.3**. The syntheses were induced thermally. The chemicals were mQ, XCt (X = Li, Na, K) and $HAuCl_4$, as indicated. The results show the effects of the cations on the temperature-induced Borowskaja-Turkevich-Frens synthesis of Au NPs.

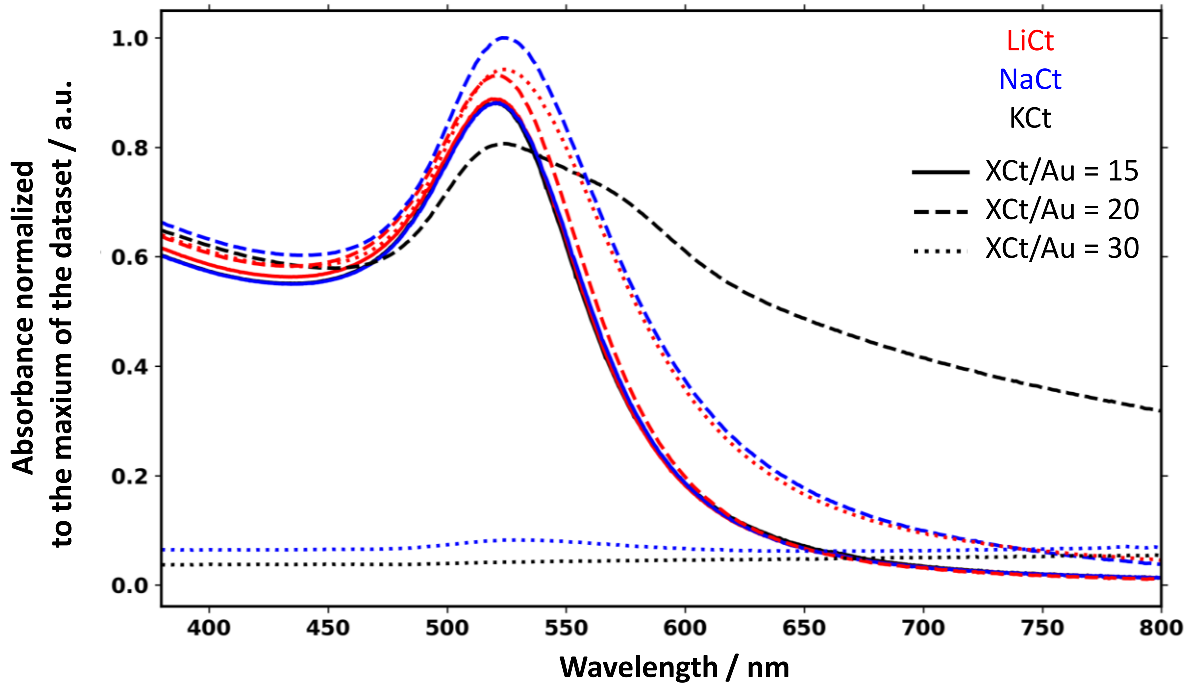

Figure S11. UV-vis spectra of Au NPs obtained using different XCt/Au molar ratios for different cations, as indicated.

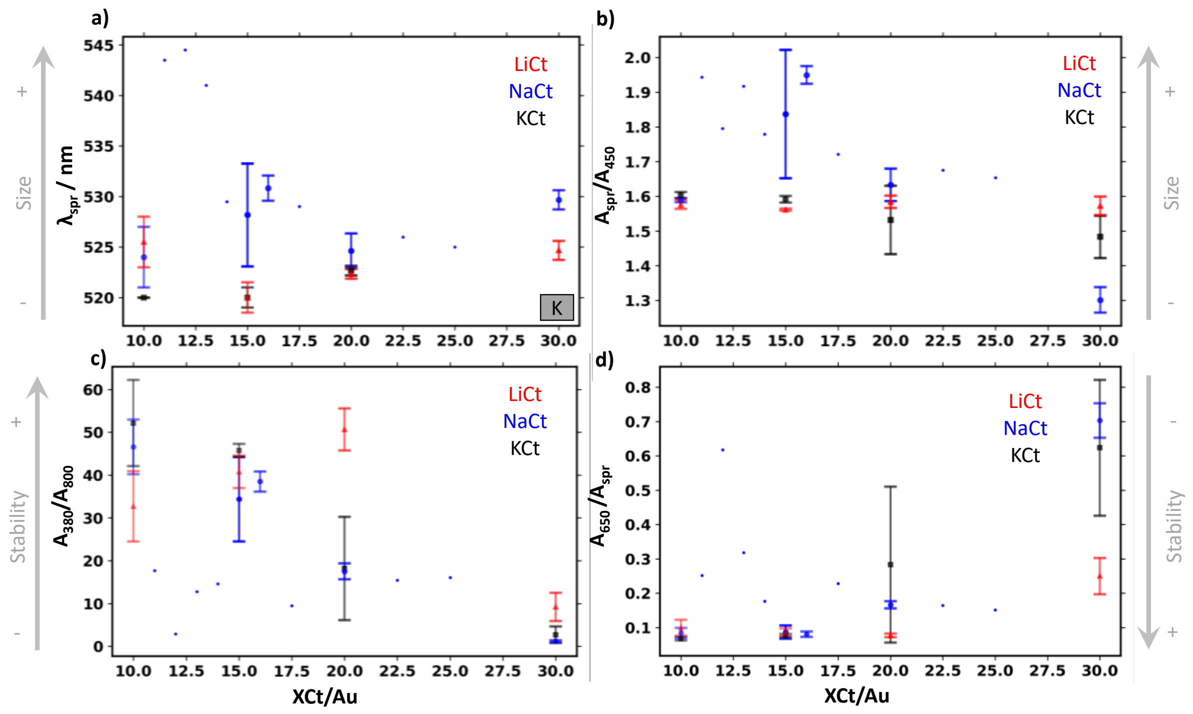

Figure S12. Various metrics retrieved from UV-vis (a) λ_{spr}, (b) A_{spr}/A_{450}, (c) A_{380}/A_{800}, (d) A_{650}/A_{spr}. The expected evolution of the size or stability with the metrics values is added to help the reader (see also **section 5.3.2**). The NPs were prepared with 0.5 mM $HAuCl_4$ and different XCt/Au molar ratios as indicated and different cations, as indicated. The grey square in (a) is to stress that for samples obtained using KCt for KCt/Au molar ratios above 20, the colloids typically show poorly defined spr and values are typically above 545 nm (meaning that the A_{spr}/A_{450} values indicated in (b) do not relate to smaller size NPs). Error bars were obtained from at least 3 replicates. The series of data points without errors bars are the values retrieved from an initial screening conducted to evaluate the feasibility of the synthesis in small volumes at high temperature, from which it was concluded that the use of UV-induced synthesis was more reliable, nevertheless the data are reported for the interest of the reader.

There is no clear trend on the effect of the cations at lower XCt/Au molar ratios below ca. 20, considering the metrics related to size (λ_{spr} and A_{spr}/A_{450}) or stability (A_{380}/A_{800} and A_{650}/A_{spr}), see **Figure S12**. However, both size and stability metrics, see **Figure S3**, indicate that larger and less stable nanomaterials are obtained when the XCt/Au molar ratio increases and this happens at relatively lower XCt/Au molar ratios with KCt, then NaCt and ultimately LiCt.

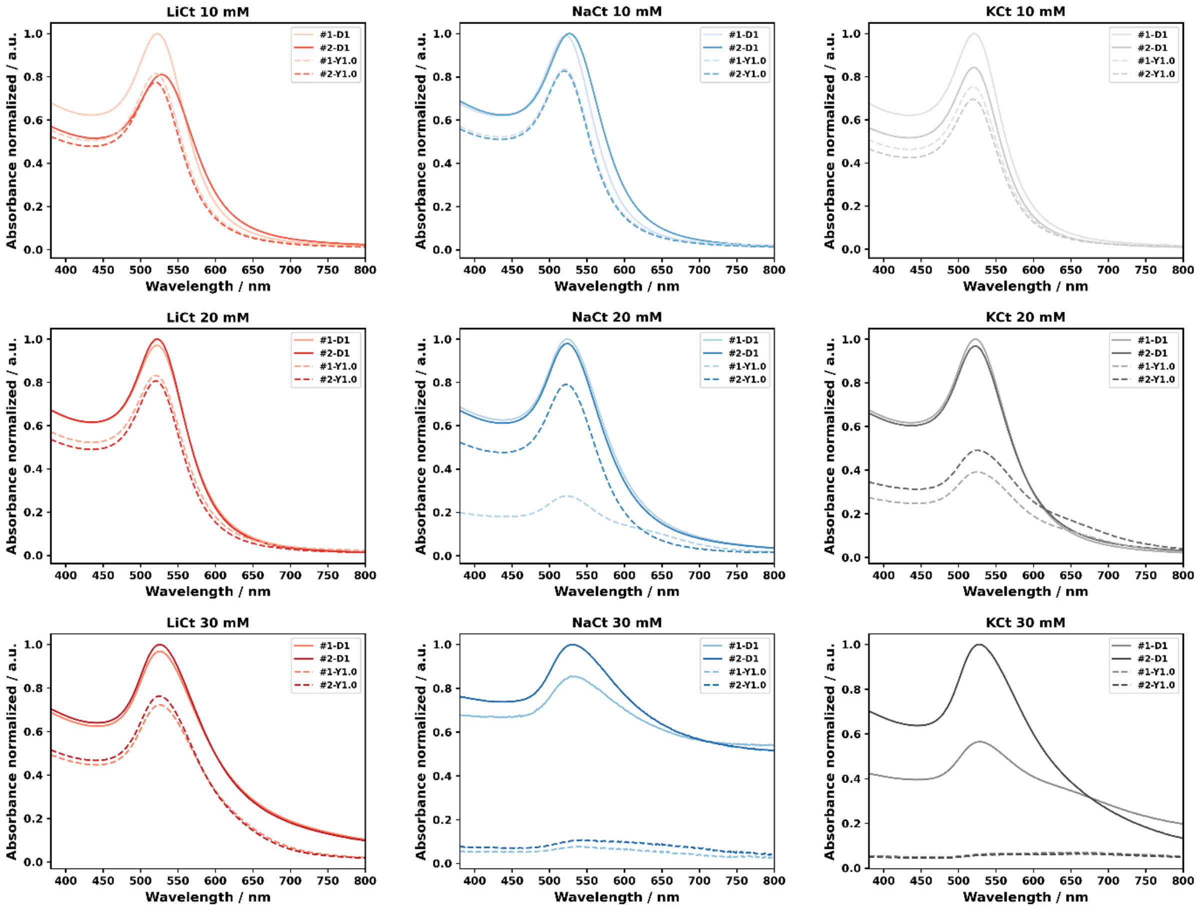

Figure S13. UV-vis spectra of colloidal Au NP dispersions obtained using different amounts of LiCt, NaCt, or KCt, as indicated, for a temperature-induced synthesis, measured a day after synthesis (D1) and a year after synthesis (Y1.0). The NPs were stored at room temperature and in a draw. Evaporation naturally happens, the volumes of the dispersions were re-adjusted to 2 mL prior to the UV-vis measurement. In all cases 0.5 mM $HAuCl_4$ was used. Duplicates are reported.

The decrease in the absorbance at 400 nm is attributed to the sedimentation of the largest Au NPs. It is rather clear that as the concentration of KCt increases, the NPs are not stable over time. This effect is also observed when NaCt is used, although slightly more stable NPs are obtained (e.g. with 20 mM NaCt compared to 20 mM KCt). The most stable colloids were obtained using LiCt, although there is also a trend that the NPs are less stable as the LiCt concentration increases.

The overall position of the λ_{spr} tends to be the same over time or to decrease, which is attributed to the fact that the largest NPs sediment, leaving a sample with overall smaller size NPs. The overall shape of the UV-vis spectra when the NPs are stable (minimal decrease in the absorbance at 400 nm, i.e. samples with LiCt or lower concentrations of NaCt or KCt) is overall the same.

The effect of LiCt vs NaCt vs KCt is in particular pronounced at higher XCt concentration: Only LiCt leads to stable NPs over time at higher concentration of 30 mM, and the trends that the stability over time decreases with LiCt > NaCt > KCt is particularly clear when 20 mM of XCt was used.

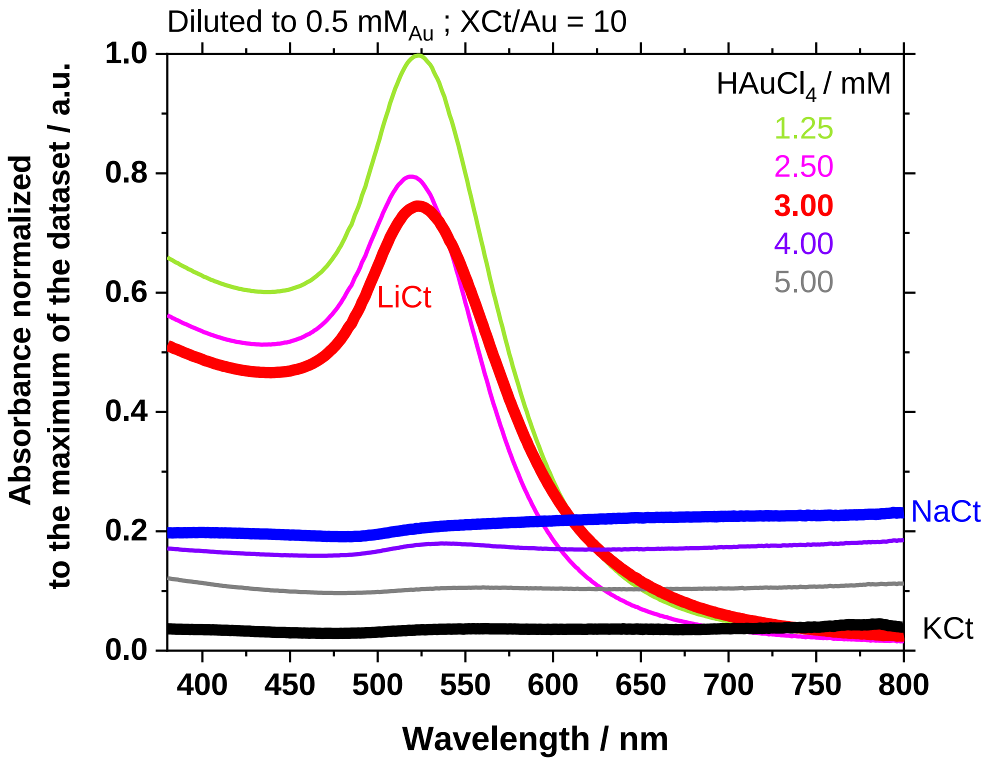

Figure S14. UV-vis spectra of Au NP colloidal dispersions obtained using different concentrations of $HAuCl_4$ for XCt/Au molar ratios of 10. For comparison, the results reported using 3 mM $HAuCl_4$ and NaCt or KCt, are indicated. The measurements were performed on dispersions diluted to 0.5 mM$_{Au}$.

As for the case of UV-induced synthesis detailed in **Figure S9**, the synthesis performed at higher $HAuCl_4$ concentration is successful when LiCt is used whereas for NaCt and KCt no stable colloids are obtained. A_{400} is reflective of the yield and stability of the Au NPs. The fact that the A_{400} value decreases for higher concentrations of precursor suggests that the yield of the synthesis is however not optimal after 24 hours (compared to using lower concentrations of $HAuCl_4$, e.g. 1.5 mM). Probably the yield could be improved by, for instance, increasing the amount of reducing agent (XCt) but in our experience too high concentrations of precursor *and* XCt do not lead to stable colloids. There was no incentive to optimize this yield at this stage to keep the focus on the effect of the cation.

The overall lower concentration at which the synthesis is successful compared to UV-induced syntheses, see **Figure S9** and **Figure S14**, can be attributed to different kinetics of formation and different stabilization expected from the different temperatures reached during the syntheses.

8. Adapted UV-induced Borowskaja-Turkevich-Frens synthesis of Au NPs with ethanol

In this section, the syntheses were performed as described in **section 5.2.4**. The syntheses were induced using a 365 nm lamp. The chemicals were mQ, ethanol, XCt (X = Li, Na, K) and $HAuCl_4$, as indicated. Ethanol was used to promote conditions where there is too much reducing agent, which leads to the formation of larger nanomaterials. The results show the effects of the cations on the adapted UV-induced Borowskaja-Turkevich-Frens synthesis of Au NPs.

The results are reported in **Figure S15** and **Figure S16**. As the ethanol content increases, larger NPs are obtained. However, smaller and more stable Au NPs are obtained, in the order from smaller to larger, with Li < Na < K, with XCt/Au molar ratio of 5 or 10. This is especially clear at higher XCt/Au molar ratio (e.g. 10) for 50-60 v.% ethanol, where stable Au NPs were only obtained using LiCt, and for 30 v.% ethanol where using NaCt leads to more stable NPs than KCt. The stability over time also decreases as LiCt > NaCt > KCt. Note that here the value of A_{400} is reflective of the yield *and* stability of the Au NPs since the Au NP colloids were measured without homogenization prior to the measurement.

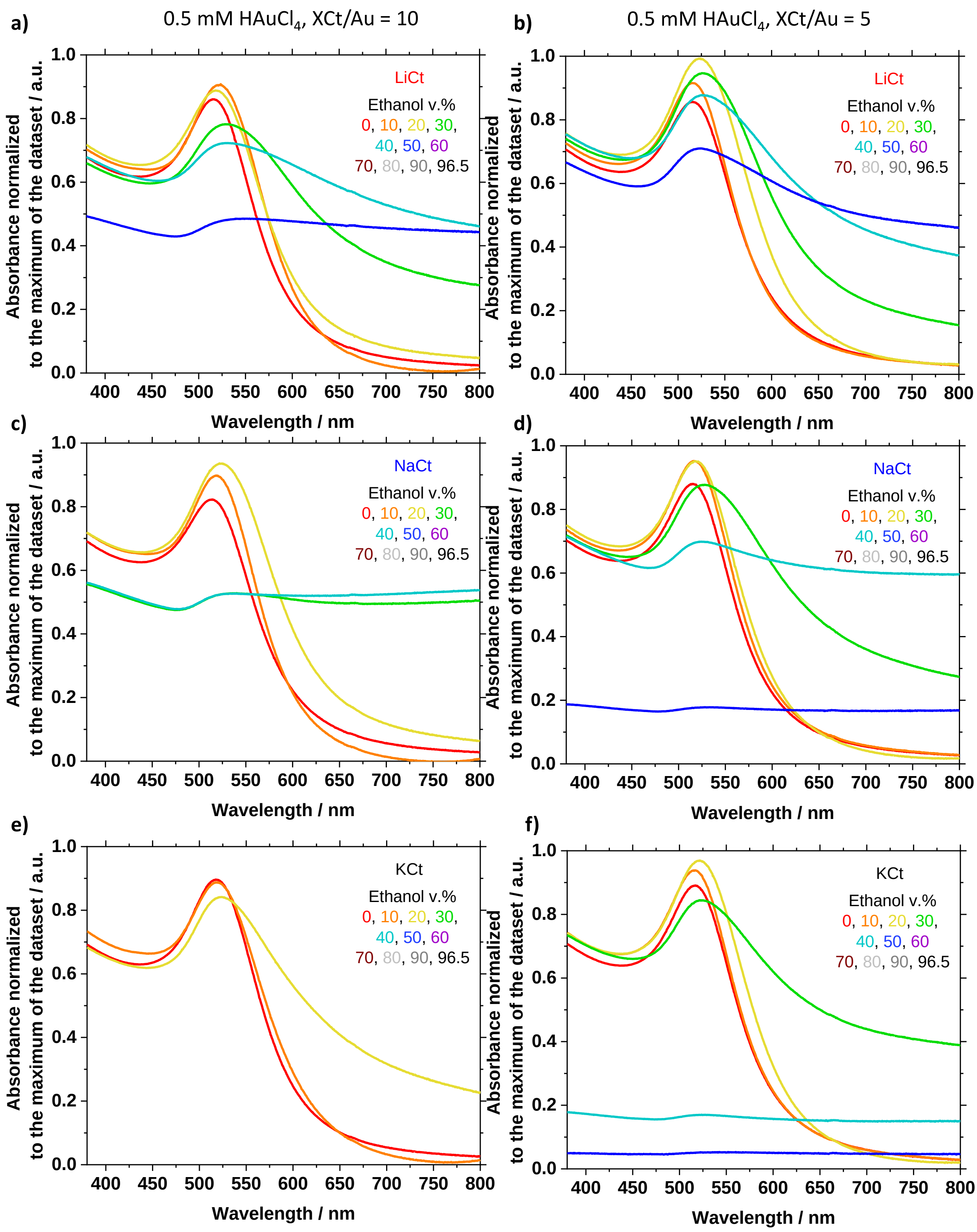

Figure S15. UV-vis spectra of Au NPs obtained using (a, b) LiCt, (c, d) NaCt, (e, f) KCt and (a, c, e) XCt/Au molar ratio of 10 and (b, d, f) XCt/Au molar ratio of 5. All experiments were performed with 0.5 mM $HAuCl_4$. All syntheses were induced by a 365 nm lamp and exposed to the controlled light of the 365 nm lamp for 2 hours. The ethanol contents investigated were 0, 10, 20, 30, 40, 50, 60, 70, 80 and 96.5 v.% ethanol in all cases. If no spectrum is reported (see color code) this means the NPs were not stable as colloids for the given amount of ethanol.

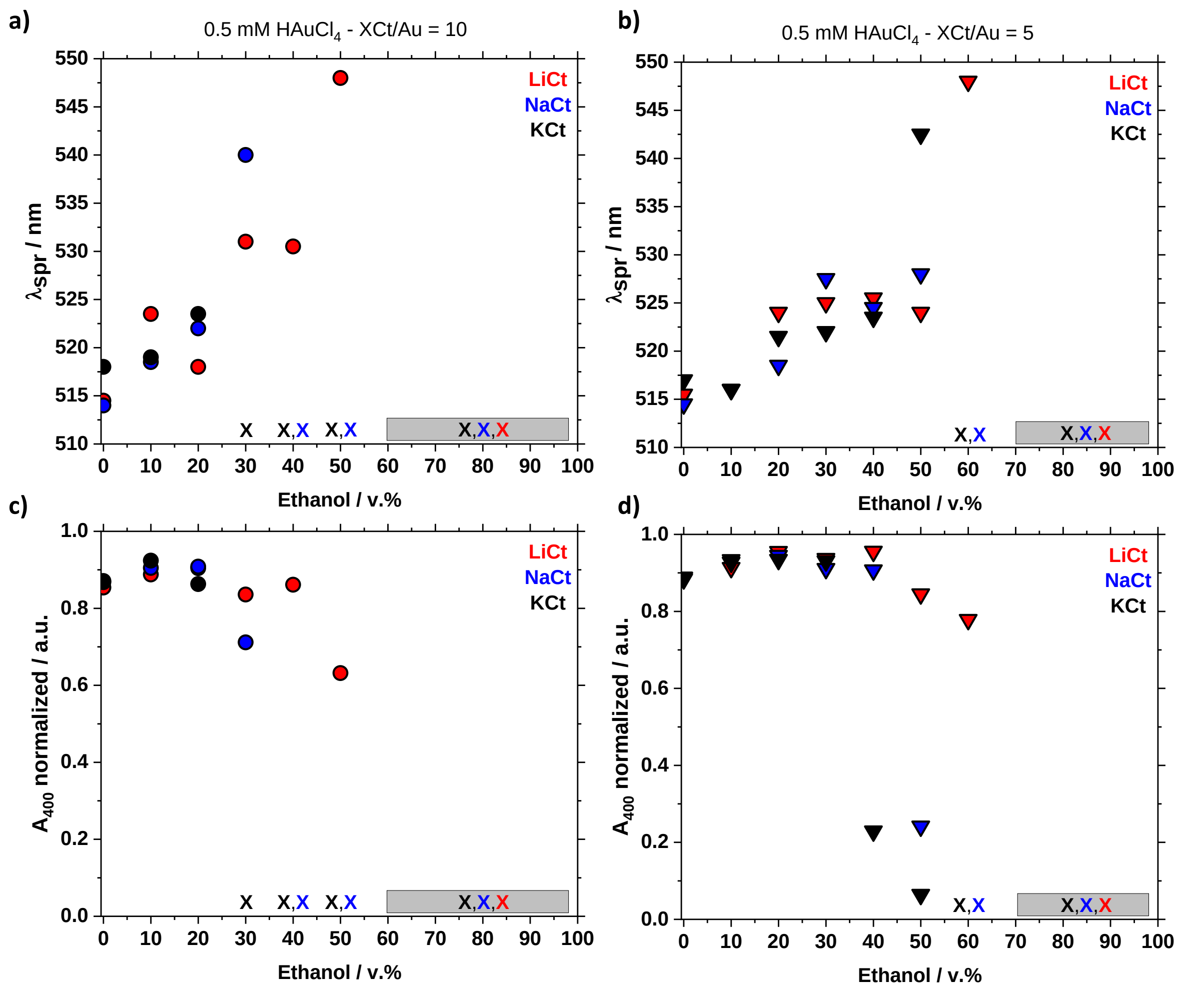

Figure S16. Metrics retrieved from UV-vis spectra of Au NPs obtained using XCt (X = Li, Na, K), as indicated: (a, b) λ_{spr} and (c, d) A_{400} (normalized). A XCt/Au molar ratio of (a, c) 10 and (b, d) 5 were used. All experiments were performed with 0.5 mM $HAuCl_4$. All syntheses were induced by a 365 nm lamp and exposed to the controlled light of the 365 nm lamp for 2 hours. The ethanol contents investigated were 0, 10, 20, 30, 40, 50, 60, 70, 80 and 96.5 v.% ethanol in all cases. A X indicates that no stable colloidal dispersions were obtained.

9. Surfactant-free BH_4-mediated synthesis of Au NPs

In this section, the syntheses were performed as described in **section 5.2.5**, following the general approach proposed by Astruc and co-workers.[28] The syntheses were induced using XBH_4 (X = Li, Na, K) at room temperature. The chemicals were mQ, XBH_4 and $HAuCl_4$, as indicated. The results show the effects of the cations on the surfactant-free XBH_4-induced synthesis of Au NPs.

Note. As summarized in **Table S4**, in contrast to the seminal work from Astruc and co-workers,[28] or Agarwal et al.,[32] we used a higher concentration of precursor (x3.8 and x2 more concentrated, respectively: 0.13 mM vs. 0.5 mM; 0.26 mM vs. 0.5 mM) and yet we observed that the surfactant-free NPs are very stable over time, over weeks, stored in a fridge or at room temperature. In contrast to previous work, we used smaller and larger volumes and also investigated a broader and finer range of $NaBH_4$/Au ratio to achieve a much finer size control. Note also that $LiBH_4$ can be selected on the ground that Li-based chemicals can be stronger reducing agent (than $NaBH_4$).[32] Finally, we investigated alternative reducing agents, i.e. KBH_4. The size controlled reported by Astruc et al.[28] or Agarwal et al.[32] was relatively poor compared to the one achieved here.

To test if the stability we observe could come from different containers used, we also performed the synthesis in Pyrex® containers without noticeable issue. To test the effect of the volume of solution used we performed a scale up to 100 mL without any major challenge (using $NaBH_4$).The NPs are stable for months (stored in a fridge). Those results possibly stress the overlooked benefits of simply obtaining Au NPs using only mQ and $NaBH_4$.

Table S4. Comparison with the literature for surfactant-free XBH_4-mediated (X = Li, Na, K) syntheses of Au NPs. RT stands for room temperature.

**Ref.**	**$HAuCl_4$** mM	**XBH_4** (X=Li, Na,K)	**$NaBH_4$/Au**	**N_2**	**Volume** mL	**Containers**	**λ_{spr}** nm	**Diameter** nm	**Stability**
28	0.13	Na	2	YES	33	Not specified (likely glass)	515	5.5 ± 2	days
			10	YES/NO			514	2.8 ± 1	1 month
			50	YES			-	-	60 min
			100	YES			-	-	10 min
32	0.26	Li	1.3	NO	250	Not specified (likely glass)	573	5-35	Stable to various pH 3-11
			2.6				550-575	5-35	
			5.2				545-550	5-35	
			10.5				512	2.2 ± 0.4	
			21.0				510	3.4 ± 0.3	
			31.4				520	3.7 ± 1.3	
**This work**	0.5	Li<br>Na<br>K	0-100	NO	2-3	PS cuvettes Pyrex®	505-535	Fine size control in the range 5-20	Months at RT or fridge

The molar ratio of XBH_4/Au investigated were: 0-20 (in steps of 1), 22, 24, 26, 28, 30, 40, 50, 100 and 200. The results are reported in **Figure S17** and **Figure S18**. At too high molar ratio above ca. 30, no stable NPs were obtained. Without XBH_4 (molar ratio of 0) the reaction does not proceeds. Below a ratio of ca. 2, there is not enough reducing agent for the synthesis to proceed. For ratios ca. 2-5 there is not enough reducing agent to obtain the fastest reduction and the NP size decreases as the ratio increases (based on λ_{spr} values). From a ratio of ca. 5, the NP size increases as the ratio increases, this is especially clear when $NaBH_4$ and KBH_4 are used. The size increase is more moderate when $LiBH_4$ is used.

The fact that the NPs obtained using KBH_4 seem relatively more stable than when $NaBH_4$ is used is attributed to the need to prepare fresh stock solutions of XBH_4, which might have slightly different concentrations and/or aged differently, which means that for replicated experiments it is not the exact same λ_{spr} values that will be obtained for a nominal XBH_4/Au molar ratio. However, the trend that larger NPs are obtained when the XBH_4/Au molar ratio increases was constantly observed on several repeated experiments provided the same stock solution of XBH_4 is used. It was also consistently observed that using $LiBH_4$, the range of $LiBH_4$/Au molar ratios where small size Au NPs were obtained was larger than when $NaBH_4$ or KBH_4 were used. In other words, the use of $LiBH_4$ leads to more *robust* syntheses, less sensitive to $LiBH_4$ stock solution preparation, a larger range of $LiBH_4$/Au molar ratios can be used and still lead to small size Au NPs with therefore a relatively finer size control.

Note that here the value of A_{400} is reflective of the yield *and* stability of the Au NPs since the Au NP colloids were measured without homogenization prior to the measurement.

The relationship between λ_{spr} and NPs diameter is illustrated in **Figure S19** together with **Figure S20**. The results confirm the correlation *larger* λ_{spr}*, larger NPs*, expected and largely validated experimentally in our previous work.[38,44,71]

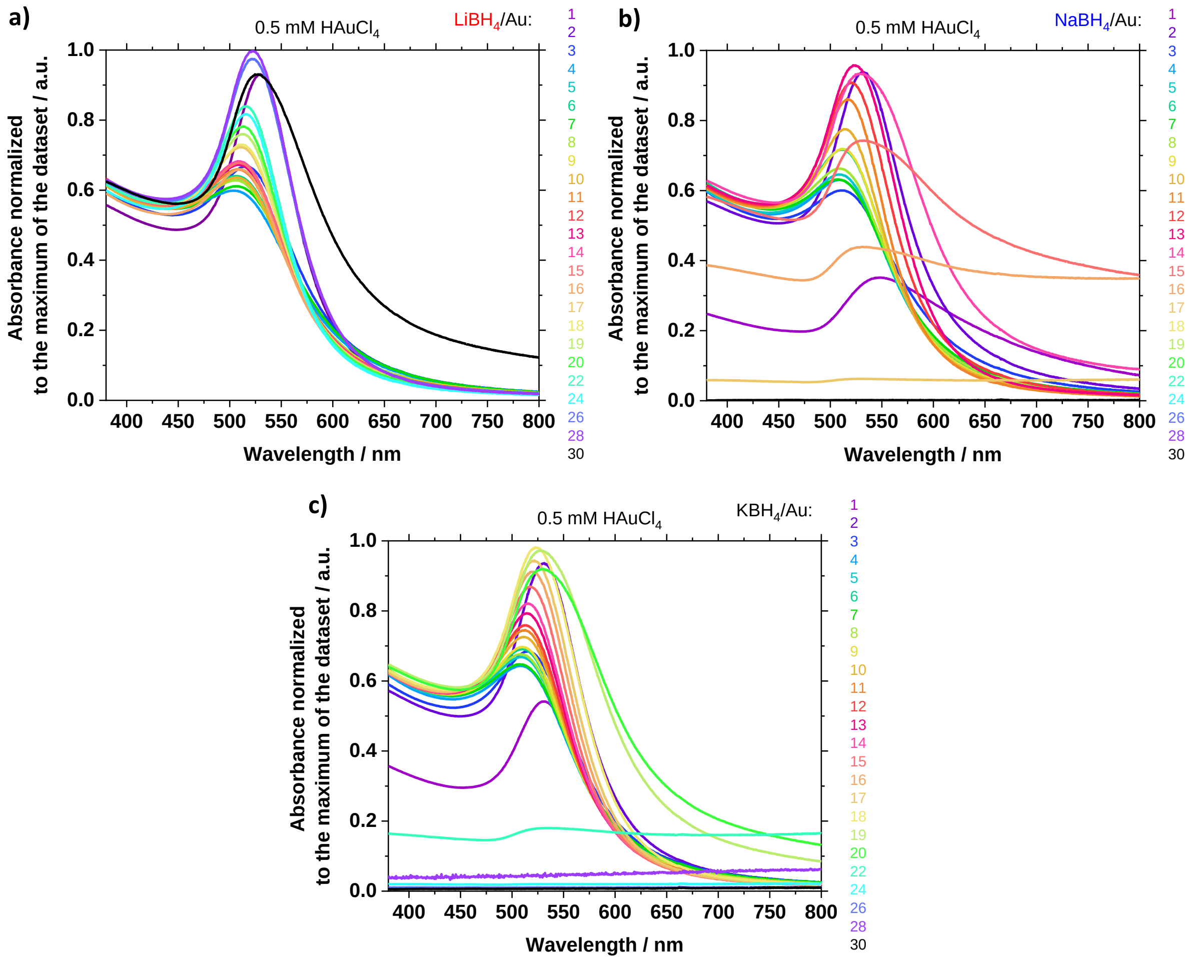

Figure S17. UV-vis spectra of Au NPs obtained with (a) $LiBH_4$, (b) $NaBH_4$ and (c) KBH_4 for different XBH_4/Au molar ratios, as indicated. All experiments were performed with 0.5 mM $HAuCl_4$. All syntheses were performed at room temperature in a photo-box. If no spectrum is reported (see indicated color code) this means the NPs were not stable as colloids for the given amount of ethanol.

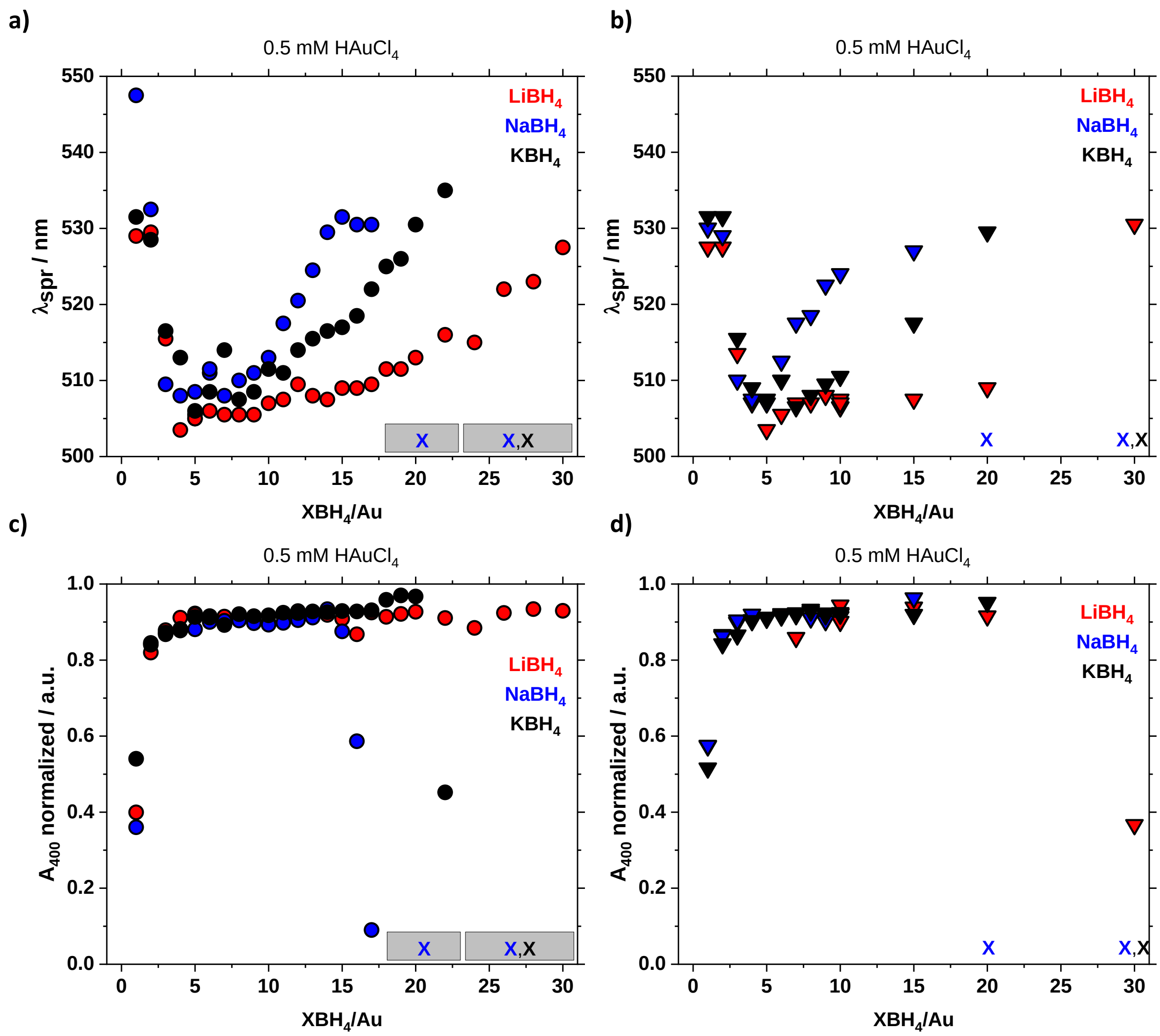

Figure S18. Data retrieved from UV-vis (a, b) λ_{spr} and (c, d) A_{400} (normalized) for Au NPs obtained with different XBH_4/Au molar ratios, and for different XBH_4 (X = Li, Na, K), as indicated. All experiments were performed with 0.5 mM $HAuCl_4$. All syntheses were performed at room temperature in a photo-box. A X indicates that the samples did not lead to a clear spr signal due to a too broad peak and/or a low absorbance (often related to sedimentation of the NPs).

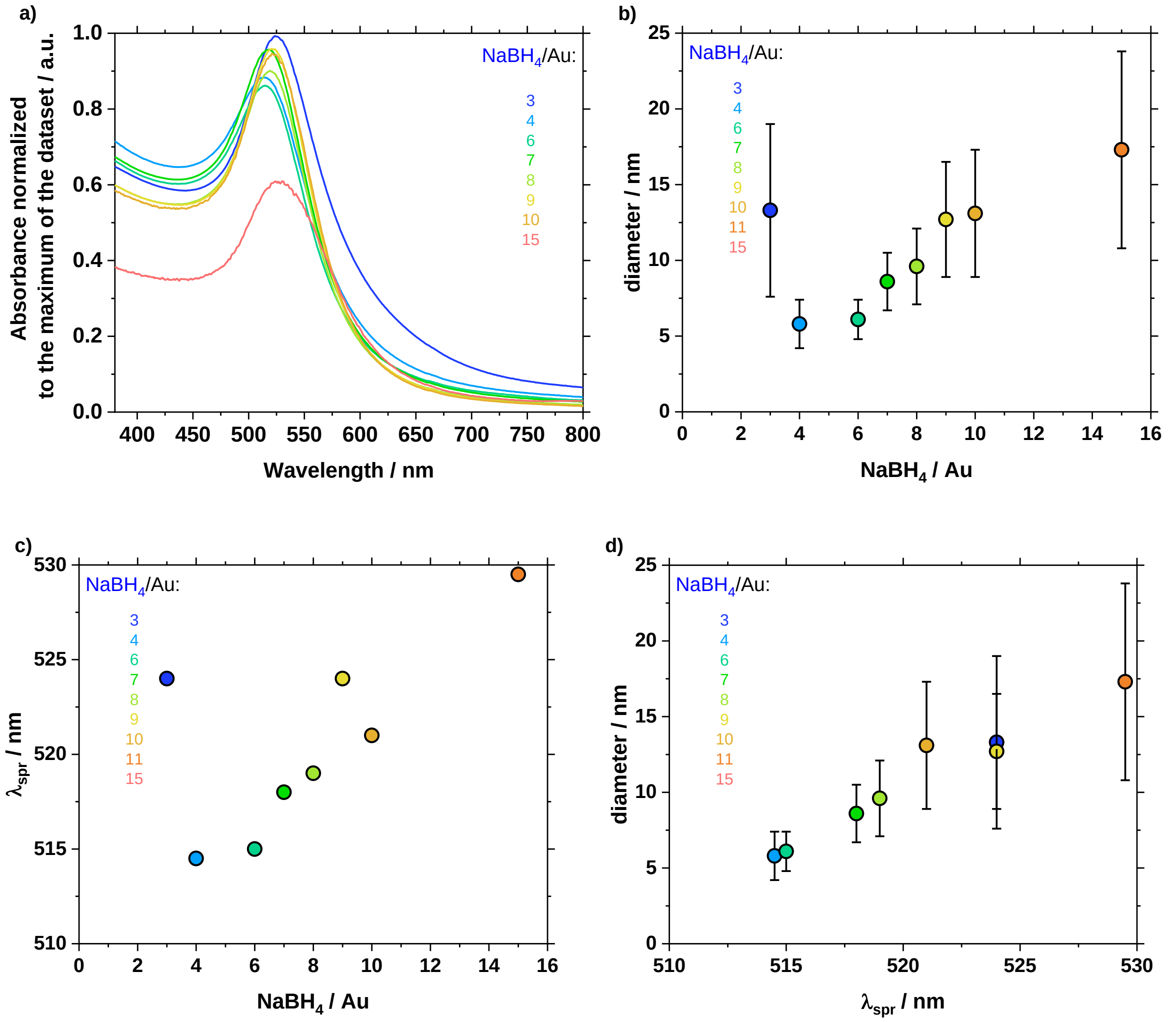

Figure S19. (a) UV-vis data of Au NPs obtained using different amounts of $NaBH_4$ for 0.5 mM $HAuCl_4$, i.e. different $NaBH_4$/Au molar ratios of 3, 4, 6, 7, 8, 9, 10 and 15 as indicated. (b) Diameter of the Au NPs as a function of the $HAuCl_4$/Au molar ratio. (c) λ_{spr} of the Au NPs as a function of the $HAuCl_4$/Au molar ratio. (d) Diameter of the Au NPs as a function of λ_{spr}. The corresponding STEM micrographs are provided in Figure S20A.

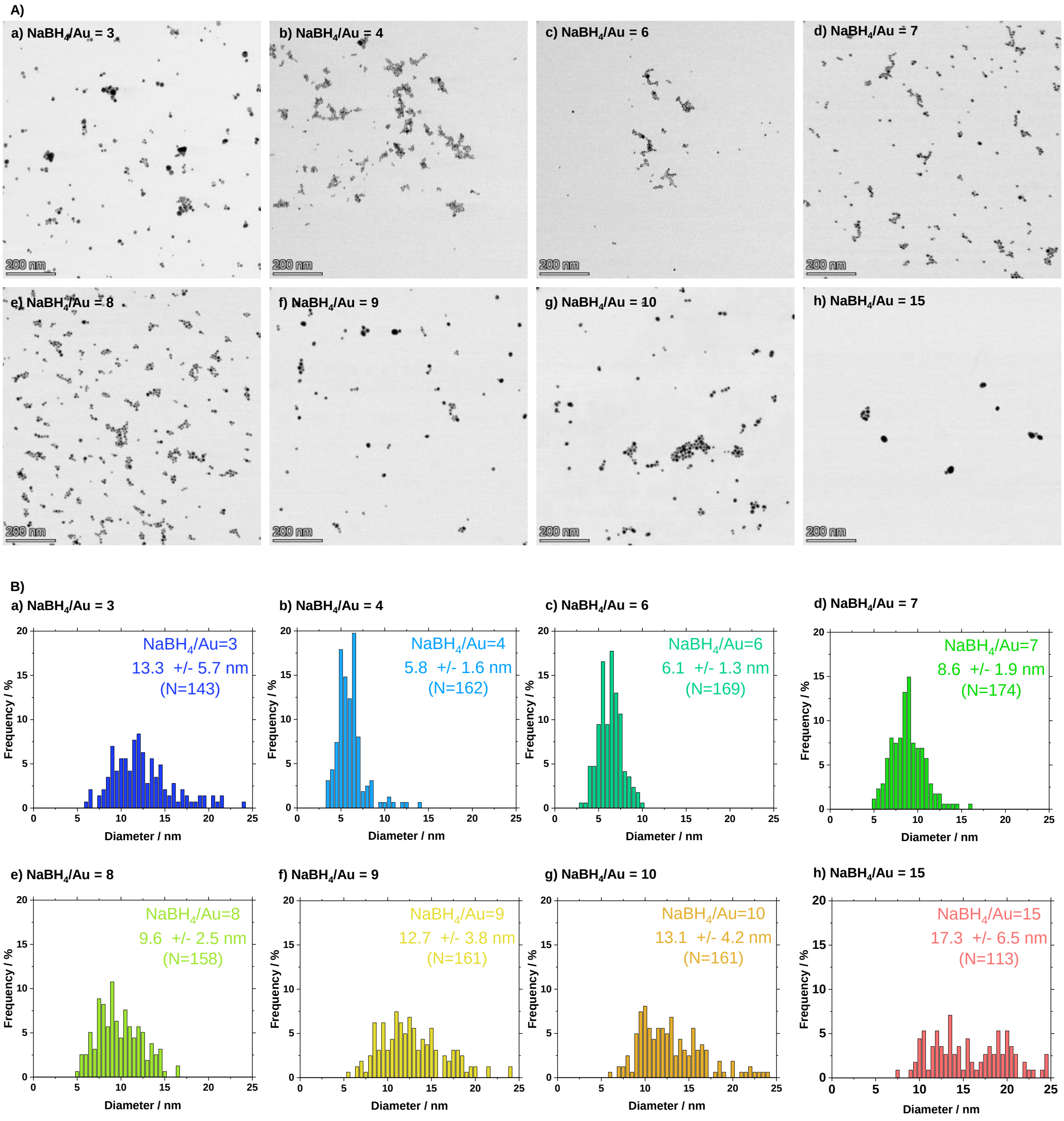

Figure S20. A) STEM micrographs (bright field detector) of Au NPs obtained using different amounts of $NaBH_4$ for 0.5 mM $HAuCl_4$, i.e. different $NaBH_4$/Au molar ratios of (a) 3, (b) 4, (c) 6, (d) 7, (e) 8, (f) 9, (g) 10, h (15). B) Corresponding size distributions, as indicated. The corresponding UV-vis spectra are displayed in Figure S19a.

We also observed that the synthesis was not sensitive to the addition of $HAuCl_4$ last, see **Figure S21** where the UV-vis spectra of Au NPs obtained adding $HAuCl_4$ last or $NaBH_4$ last did not lead to significant difference. In the present study, to use the stock solution of XBH_4 as fresh as possible, the XBH_4 was preferentially added last.

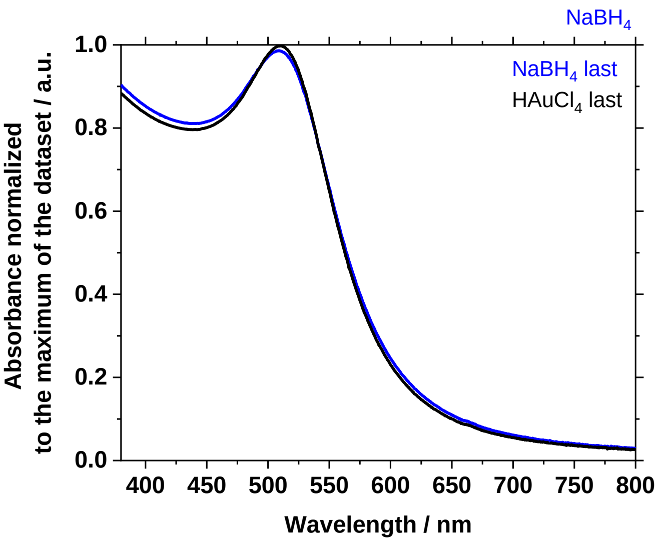

Figure S21 UV-vis spectra of Au NPs prepared using $NaBH_4$ at 2.5 mM and 0.5 mM $HAuCl_4$ ($NaBH_4$/Au molar ratio of 5) in water prepared at room temperature. The NPs were obtained by adding $NaBH_4$ last of $HAuCl_4$ last, as indicated.

We also made sure that the synthesis was scalable and that the NPs obtained were stable, since the preparation previously reported pointed towards a lack of stability of the $NaBH_4$-prepared NPs. We did not observed any challenge with stability even after extended period of time as illustrated in **Figure S22** where the spectra of as-prepared colloidal dispersion or after 2.5 months of storage in a fridge are very similar.

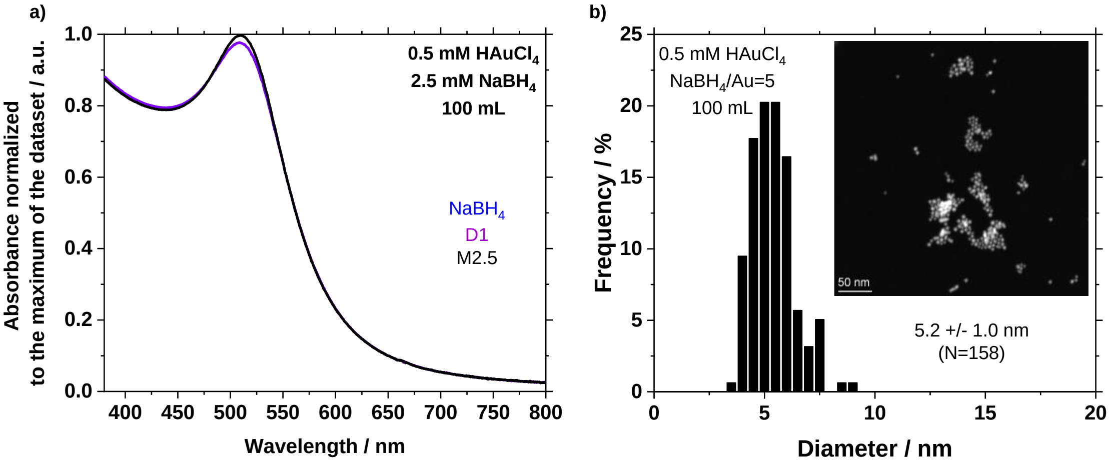

Figure S22. (a) UV-vis spectra of Au NPs prepared using $NaBH_4$ at 2.5 mM and 0.5 mM $HAuCl_4$ ($NaBH_4$/Au molar ratio of 5) in water prepared at room temperature for a total volume of 100 mL and for a solution kept in a fridge for 2.5 months. The spectra recorded the day after synthesis (D1) and after 2.5 months (M2.5) are reported with a λ_{spr} around 508 nm. (b) Corresponding size distribution and STEM data (inset).

10. Adapted surfactant-free BH_4-mediated synthesis of Au NPs with added ethanol

In this section, the syntheses were performed as described in **section 5.2.6**, following the general approach proposed by Astruc and co-workers.[28] The syntheses were induced using mQ, ethanol, XBH_4 (X = Li, Na, K) at room temperature. The chemicals were mQ, ethanol, XBH_4 and $HAuCl_4$, as indicated. Ethanol was used to promote conditions where there is too much reducing agents which leads to the formation of larger nanomaterials. The results show the effects of the cations on the adapted surfactant-free XBH_4-induced synthesis of Au NPs.

The results of the comparison of the use of $LiBH_4$, $NaBH_4$ or KBH_4 using 0, 10, 20, 30, 40, 50, 60, 70, 80, 96.5 v.% ethanol are reported in **Figure S23**. Overall $LiBH_4$ leads to smaller NPs for a given content of ethanol but also over a wider range of ethanol contents. The stability of the Au NPs obtained using $LiBH_4$ is higher.

Note that here the value of A_{400} is reflective of the yield *and* stability of the Au NPs since the Au NP colloids were measured without homogenization prior to the measurement.

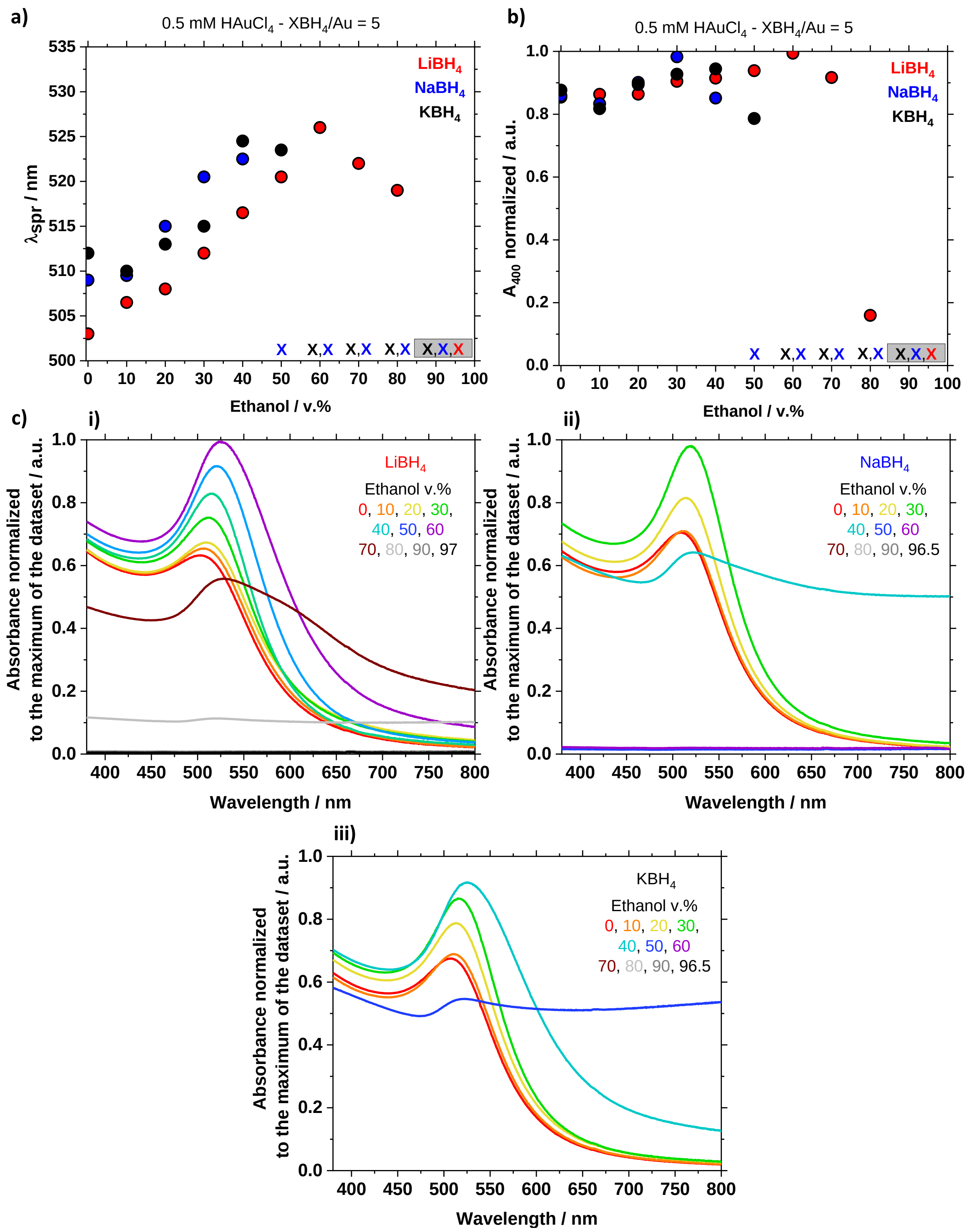

Figure S23. Data retrieved from UV-vis (a, b) λ_{spr} and (c, d) A_{400} (normalized) for Au NPs obtained with different XBH_4 (X = Li, Na, K), as indicated, with XBH_4/Au molar ratio of 5, for different amounts of ethanol added, as indicated. All experiments were performed with 0.5 mM $HAuCl_4$. All syntheses were performed at room temperature in a photo-box. A X indicates that the samples did not lead to a clear spr signal due to a too broad peak and/or a low absorbance (often related to sedimentation of the NPs). The ethanol contents was 0, 10, 20, 30, 40, 50, 60, 70, 80, 96.5 v.% ethanol. If no spectrum is reported (see color code) this means the NPs were not stable as colloids for the given amount of ethanol.

11. Surfactant-free synthesis of Au NPs in alkaline water-ethanol mixtures

In this section, the syntheses were performed as described in **section 5.2.7**, following the general surfactant-free approach proposed by Quinson and co-workers.[38] The syntheses were induced using mQ, ethanol, XOH (X = Li, Na, K) at room temperature. The chemicals were mQ, ethanol, XOH and $HAuCl_4$, as indicated. The results show the effects of the cations on the surfactant-free syntheses of Au NPs obtained using alkaline water-ethanol mixtures.

Based on the λ_{spr} values, see for instance **Figure S24a**, the smallest NPs are obtained using LiOH, larger NPs are obtained using NaOH and even larger NPs are obtained using KOH, at a given concentration of XOH and for 20 v.% ethanol, whether the $HAuCl_4$ concentration was 0.5 mM or 0.2 mM. For instance, for 0.5 mM $HAuCl_4$ the λ_{spr} values are 518, 523 and 538 nm when LiOH, NaOH and KOH are used, respectively, which correspond to sizes of 9.5 ± 2.6, 14.2 ± 5.1, 22.4 ± 17.2 nm. The sizes are retrieved from STEM analysis detailed in **Figure S30**, discussed later. The trends that NPs obtained using LiOH give smaller size NPs compared to when NaOH is used, and that the largest NPs are obtained with KOH, are confirmed for various XOH/Au molar ratios, see **Figure S24b-c.**

The synthesis is reported to depend on the XOH/Au molar ratio as illustrated in **Figure S24d-f**. It is also observed that for a higher concentration of $HAuCl_4$ (0.5 mM) the NP size is less sensitive to the concentration of base when LiOH is used. Those results are in agreement with our general observation that LiOH leads to smaller and more stable NPs compared to NaOH and that the use of KOH leads to less reproducible results and poorer size control.[38,44]

Note that here the value of A_{400} is reflective of the yield *and* stability of the Au NPs since the Au NP colloids were measured without homogenization prior to the measurement.

Overall, the use of LiOH leads to smaller NPs (lower λ_{spr} values) than NaOH or KOH and at high concentration of $HAuCl_4$ (0.5 mM) the use of different amounts of base has less of an effect that when NaOH or KOH is used.

After centrifugation, as described in **section 5.3.5**, the NPs prepared using LiOH are relatively more stable than those obtained using NaOH or KOH (less pronounced decrease in the absorbance at 400 nm after centrifugation), see **Figure S25a**. This can be attributed to the relatively smaller size of NPs obtained with LiOH based on the corresponding λ_{spr} values, however for Au NPs obtained with 0.2 mM $HAuCl_4$ and with similar UV-vis spectra for syntheses using NaOH and KOH, and/or showing features suggesting less stable Au NPs for LiOH (higher absorbance at lower wavelengths), the trend that the Au NPs obtained using LiOH are relatively more stable than when NaOH or KOH is used (with NPs obtained using NaOH being relatively more stable than those obtained using KOH) is still observed as reported in **Figure S25b,c.**

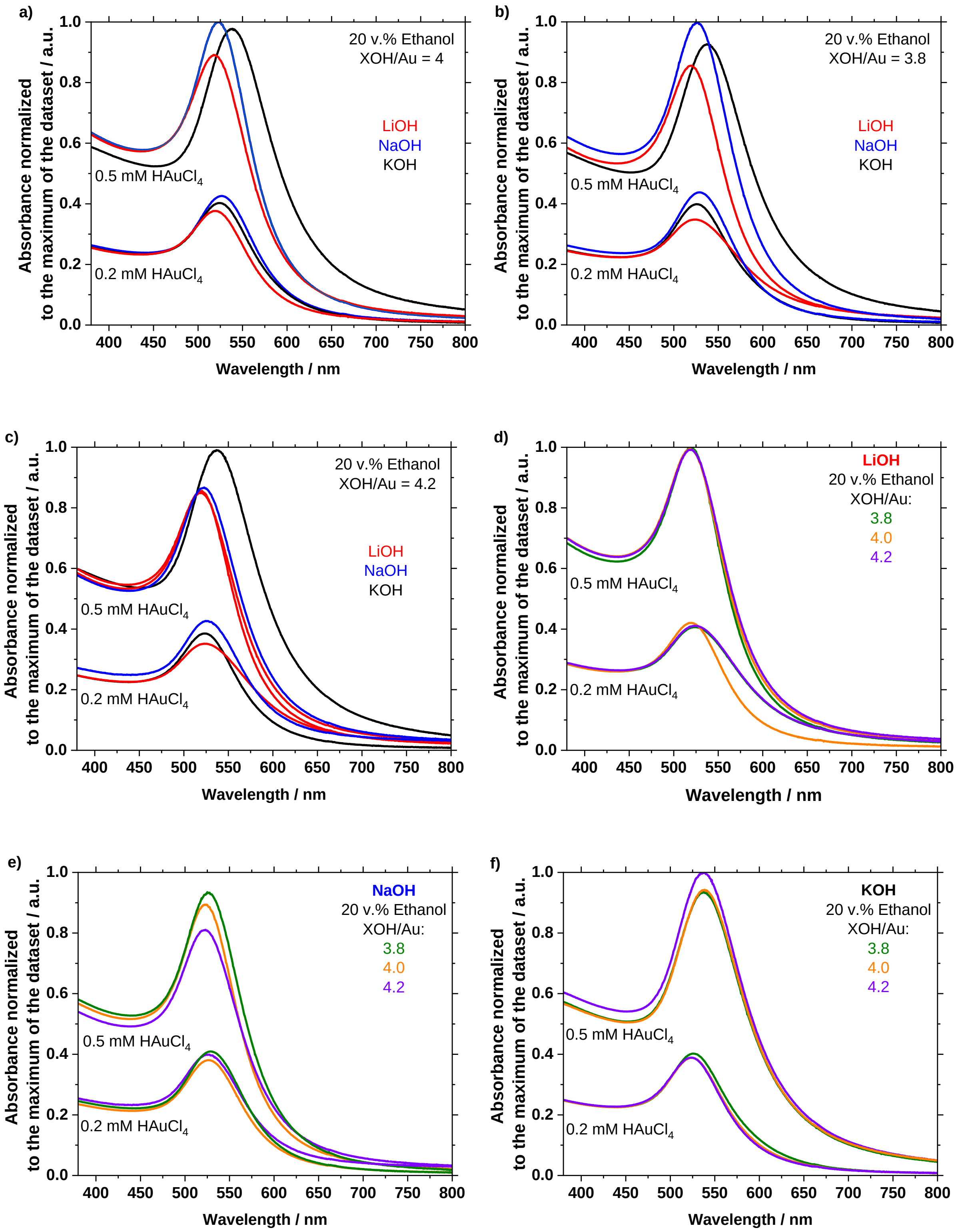

Figure S24. UV-vis spectra of surfactant-free Au NPs obtained using 20 v.% ethanol and 0.2 or 0.5 mM $HAuCl_4$, as indicated, and a XOH/Au molar ratio of 3.8, 4.0 or 4.2, as indicated, for different bases LiOH, NaOH, or KOH, as indicated.

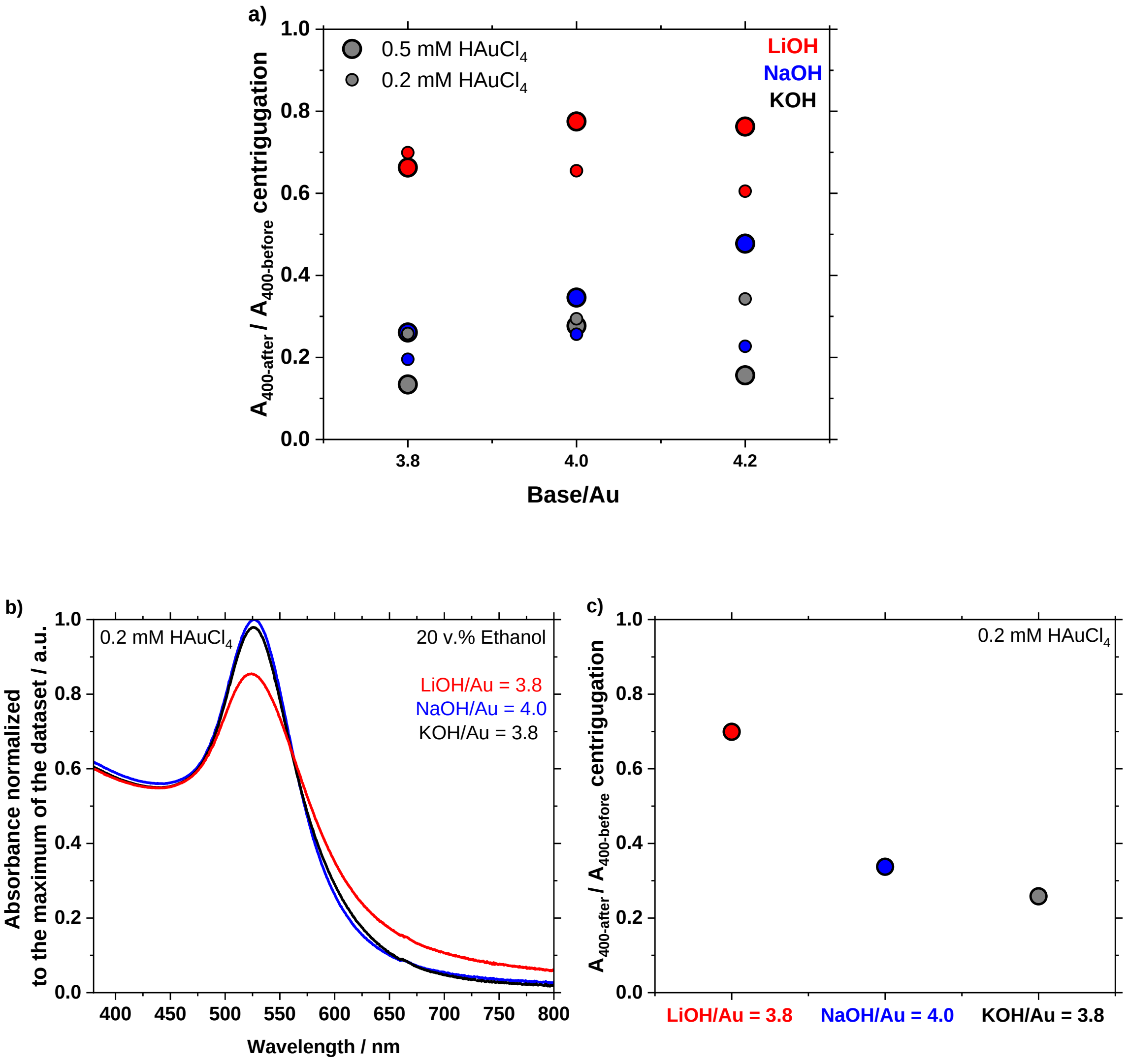

Figure S25. (a) Stability test by centrifugation for surfactant-free Au NPs obtained using 20 v.% ethanol, 0.2 or 0.5 mM $HAuCl_4$, as indicated, and using different XOH/Au molar ratios of 3.8, 4.0 or 4.2, and for which XOH is LiOH, NaOH or KOH, as indicated. (b) UV-vis spectra of Au NPs obtained using LiOH, NaOH or KOH and different XOH/Au molar ratios, as indicated, leading to relatively similar UV-vis spectra and (c) the results of the corresponding centrifugation test.

12. Surfactant-free synthesis of Au NPs in alkaline water-ethanol mixtures with lower grade chemicals

In this section, the syntheses were performed as described in **section 5.2.7**, following the general surfactant-free approach proposed by Quinson and co-workers.[38] The syntheses were induced using mQ, ethanol, XOH (X = Li, Na, K) at room temperature. The chemicals were mQ, DI water, ethanol (lower grade), XOH and $HAuCl_4$, as indicated. The results show the effects of the cations on the surfactant-free syntheses of Au NPs obtained using alkaline water-ethanol mixtures in lower purity chemicals.

Figure S26 gathers the results obtained when amounts of DI water of 0, 2.5, 5,0, 7.5, 10.0, 12.5, 15.0, 17.5, 20.0, 25.0, 30.0, 35.0, 40.0, 45.0, 50.0, 60.0 and 65.0 v.% were used (for a total of 80 v.% water (mQ+DI) and 20 v.% ethanol). The use of KOH leads to higher λ_{spr} values indicative of larger NPs being formed. The use of LiOH or NaOH leads to similar λ_{spr} values although as the amount of DI water increases the λ_{spr} values are smaller when LiOH is used. Those results indicate that in case where the NPs are less stable and/or tend to grow larger (high DI water contents) the use of LiOH leads to smaller and more stable NPs. In other words, the synthesis can be considered more *robust* when LiOH is used. This is especially clear in **Figure S26c** where the A_{400} value (normalized) is reported.

Note that here the value of A_{400} is reflective of the yield *and* stability of the Au NPs since the Au NP colloids were measured without homogenization prior to the measurement.

Another way to assess the benefits of using LiOH is to consider cases where the synthesis using NaOH does not lead to stable Au NPs, which correspond to amounts of DI water around 30-50 v.%, as illustrated in **Figure S27**. While large agglomerated NPs are obtained using NaOH for DI contents of 35-50 v.%, relatively small size Au NPs are still obtained using LiOH. The use of NaOH leads to rather large NPs with a broad size distribution and around 40 nm in size, whereas the use of LiOH leads to NPs ca. 15-20 nm.

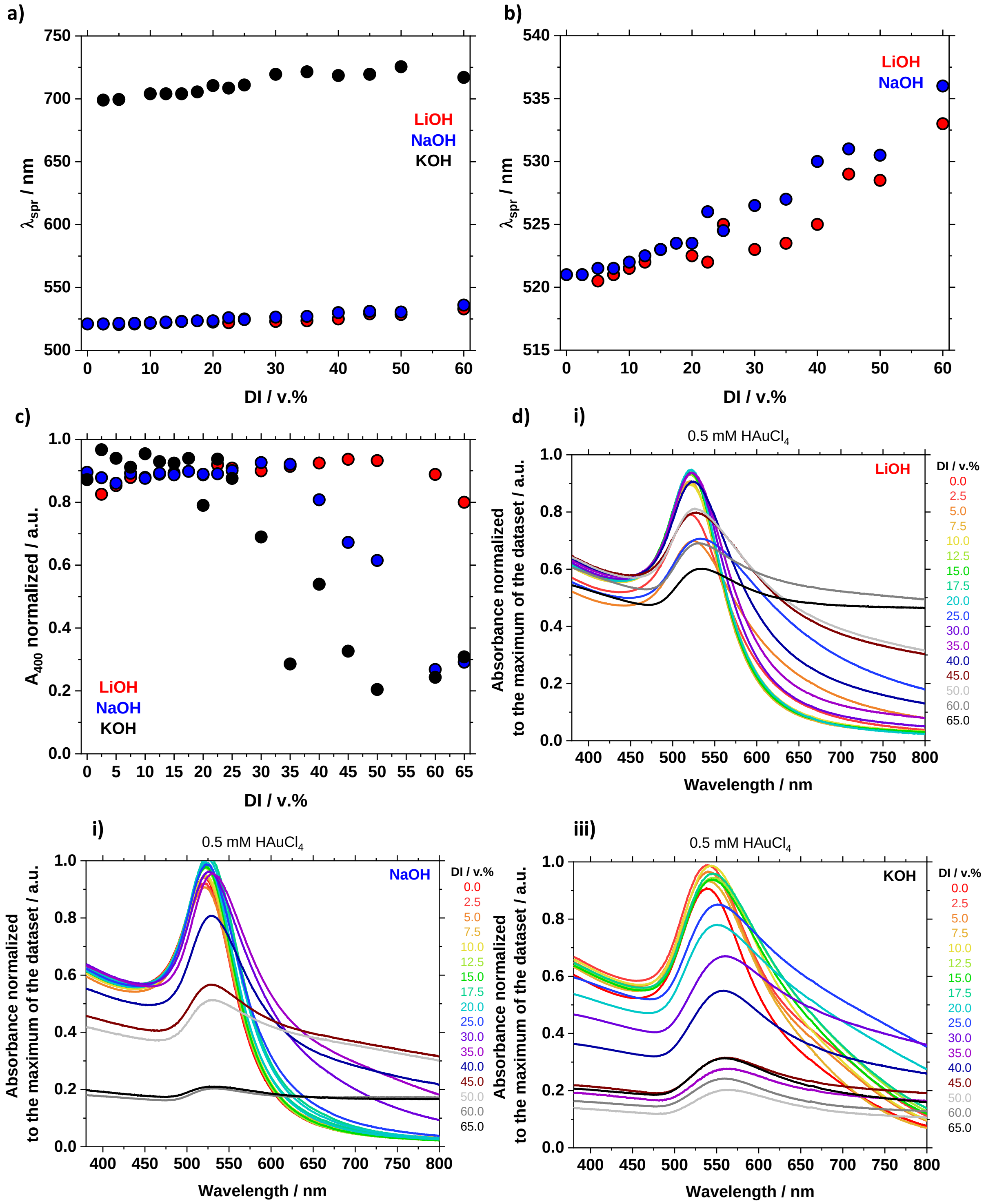

Figure S26. Data retrieved from UV-vis (a, b) λ_{spr} and (c, d) A_{400} (normalized) for surfactant-free Au NPs obtained with different amounts of DI water and for different bases XOH (X = Li, Na, K), as indicated. (a) and (b) correspond to the same data with a different Y-axis scale. All experiments were performed with 0.5 mM $HAuCl_4$, 2 mM of XOH (XOH/Au molar ratio of 4) using 20 v.% of ethanol (70% grade) and 80 v.% water. The water was a mixture of mQ and DI water where the DI water content is reported on the X-axis. (d) Corresponding UV-vis spectra for (i) LiOH, (ii) NaOH and (iii) KOH. Data related to NaOH are reported in [62] and reproduced here for a better comparison.

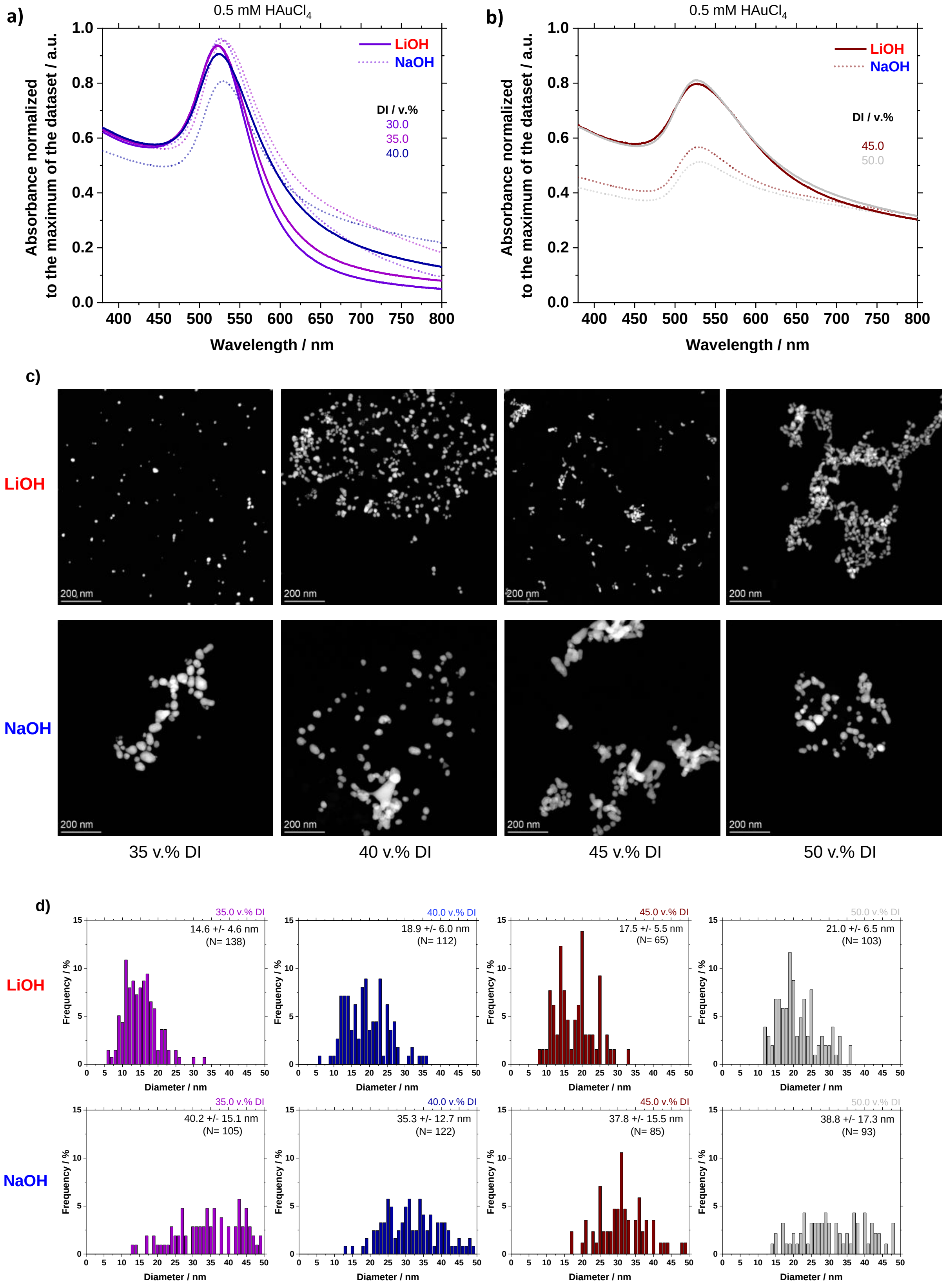

Figure S27. (a, b) UV-vis spectra of Au NP colloidal dispersions for surfactant-free Au NPs obtained with different amounts of DI water and for different bases XOH (X = Li, Na), as indicated. In all cases, 20 v.% ethanol (70% grade) and 0.5 mM $HAuCl_4$, with LiOH and NaOH, as indicated, for a XOH/Au molar ratio of 4, were used. The total amount of water was 80 v.% but this total amount of water was a mix of mQ and DI water, for different amounts of DI water,

as indicated. The same spectra are reported in **Figure S26** but individualized here for clarity. (c) Corresponding STEM micrographs and (d) related size distributions of the surfactant-free Au NPs for DI water contents as indicated and corresponding to DI contents of 35, 40, 45 and 50 v.%. Data related to NaOH are reported in [62] and reproduced here to facilitate the comparison.

13. Surfactant-free synthesis of Au NPs in alkaline water-ethanol mixtures with citrate additives

In this section, the syntheses were performed as described in **section 5.2.8**, following the general surfactant-free approach proposed by Quinson and co-workers.[38] The syntheses were induced using mQ, ethanol, XOH (X = Li, Na, K) and XCt (X = Li, Na, K) at room temperature. The chemicals were mQ, ethanol, XOH, XCt and $HAuCl_4$, as indicated. The results show the effects of the cations on the surfactant-free syntheses of Au NPs obtained using alkaline water-ethanol mixtures with both a base and citrate.

For a given XOH, the corresponding XCt with the same X (Li, Na, K) were used. We previously showed that there are no benefits of using XCt in the otherwise optimized surfactant-free syntheses using a NaOH/Au molar ratio around 4, 0.5 mM $HAuCl_4$ and 20 v.% ethanol.[36] We here investigate the effect of the cation on promoting such conditions where larger NPs are expected to form as the amount of XCt increases and include LiCt and KCt as source of the citrates.

UV-vis spectra of Au NPs obtained using 20 v.% ethanol and 0.2 or 0.5 mM $HAuCl_4$, for a XOH/Au molar ratio of 4 and different XCt/Au molar ratios (0, 5, 10, 15), as indicated, are reported in **Figure S28**. The UV-vis spectra obtained for different ethanol contents and XCt/Au molar ratio are reported in **Figure S29**. The corresponding STEM micrographs for Au NPs obtained using 20 v.% ethanol, a XOH/Au molar ratio of 4, are reported in **Figure S30** for a XCt/Au molar ratio of 0, in **Figure S31** for a XCt/Au molar ratio of 5, in **Figure S32** for a XCt/Au molar ratio of 10 and in **Figure S33** for a XCt/Au molar ratio of 15. In all cases the same cation used for the XOH/XCt couple considered (where X = Li, Na, K). Note that the data related to NaCt/Au = 0, 5, 10, 15 are already reported in [36] and reproduced here to facilitate the comparison with LiCt and KCt.

Upon adding XCt to the surfactant-free synthesis of Au NPs (see **section 11**), larger and less stable NPs are obtained for both NaCt and KCt, where NPs obtained using KCt tend to be larger than when NaCt is used. In all cases the smallest and most stable Au NPs are obtained using LiCt (e.g. up to XCt/Au molar ratio of 15, value for which using NaCt or KCt does not lead to stable Au NPs). The trend that increasing NP sizes are observed with the cation is well observed for a XCt/Au molar ratio of 10 and a relatively high $HAuCl_4$ concentration of 0.5 mM, see **Figure S32**, for which the size of the Au NPs increases with $Li^+ < Na^+ < K^+$.

Furthermore, for similar ethanol content, XOH/Au molar ratio of 4, and XCt/Au molar ratios of 0, 5, 10, 15, it is worth pointing that the λ_{spr} values are smaller and the spr peak better defined when X = Li, **Figure S29**, than when X = Na, Figure S3 of [36]. These results stress the benefits of Li^+ cation for controlling the synthesis towards more stable NPs even when the ethanol content decreases and the citrate concentration increases.

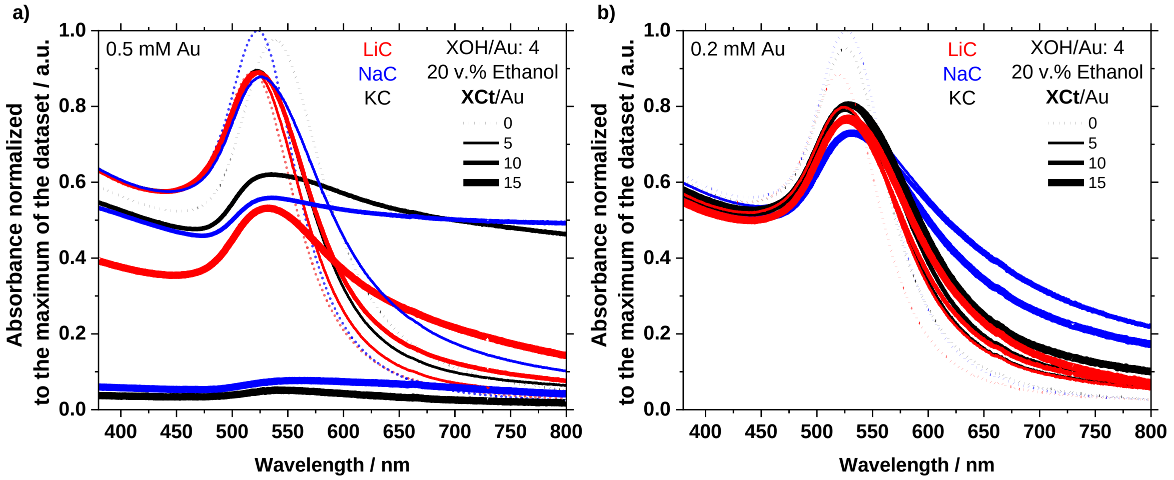

Figure S28. UV-vis spectra of Au NPs obtained using 20 v.% ethanol and (a) 0.5 mM or (b) 0.2 mM $HAuCl_4$, for different bases XOH and citrate XCt (X = Li, Na, K), as indicated for different XCt/Au molar ratios. Regardless of the base used, LiOH, NaOH, or KOH, the XOH/Au molar ratio was always 4. The XCt/Au molar ratio was 0, 5, 10 or 15, as indicated. In all cases the same cations was used for the XOH/XCt couple considered (where X = Li, Na, K). The corresponding STEM data are given in Figure S30-31.

Note that the UV-vis data related to NaCt/Au = 0, 5, 10, 15 are already reported in [36] and reproduced here to facilitate the comparison with LiCt and KCt. The results are also gathered in a 2D plots proposed in **Figure S35** detailed later.

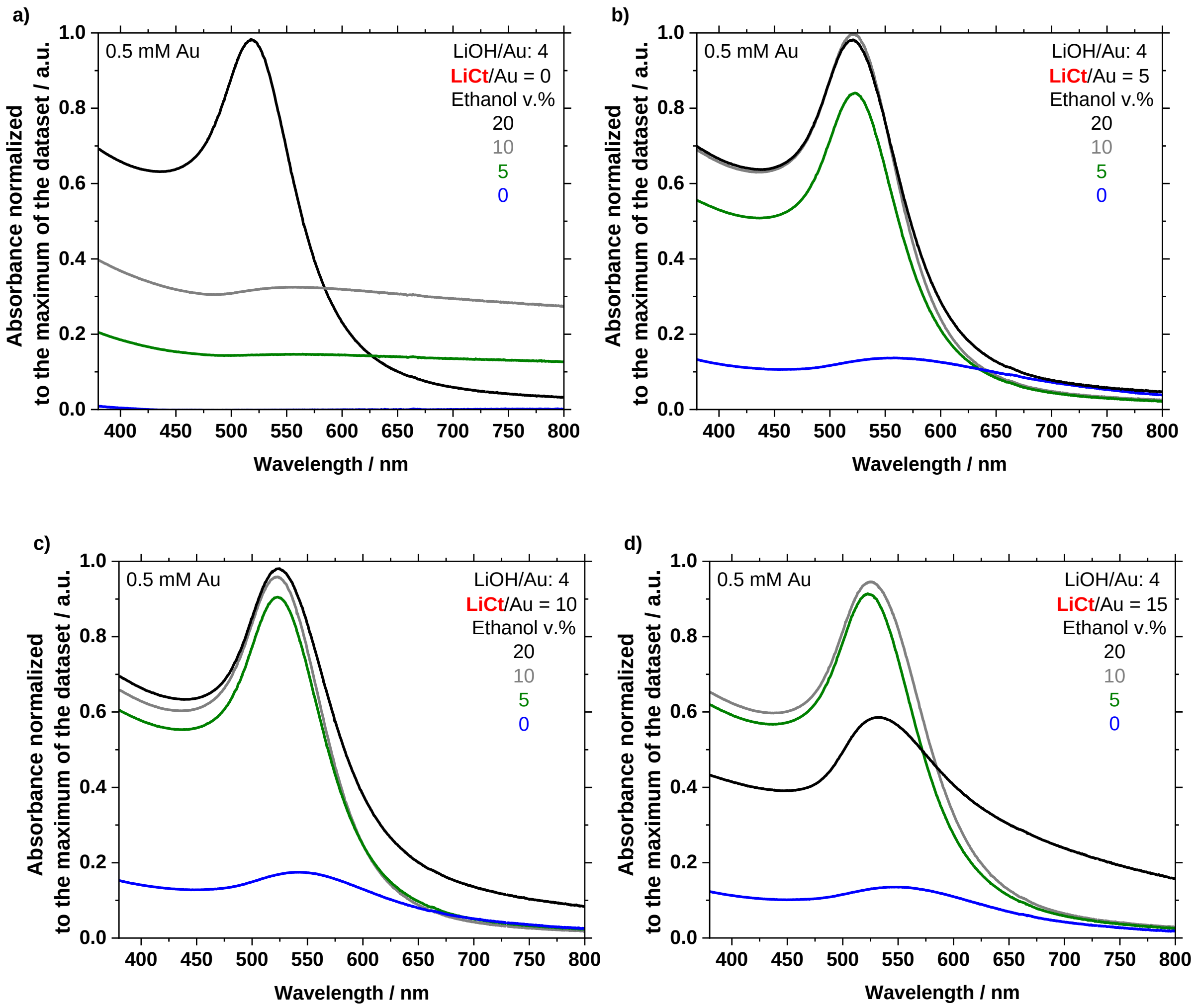

Figure S29. UV-vis spectra of Au NPs obtained using 20 v.% ethanol and 0.5 mM $HAuCl_4$, as indicated and a LiOH/Au molar ratio of 4, as indicated, using LiCt. The LiCt/Au molar ratio was 0, 5, 10 or 15, as indicated.

Note. Since citrate and alkaline solutions of ethanol can both play the role of reducing agents, it is intuitive that increasing the amount of citrate as the ethanol content decreases might lead to an optimum. Reducing the amount of ethanol as the amount of citrate increases leads in most cases to samples with relatively well defined spr. In the case where no ethanol was used, and in presence of LiCt the reaction still proceeds but the Au NPs are typically larger (higher λ_{spr}) than when a 365 nm light or temperature is used (see **sections 6 and 7**) to induce the synthesis.

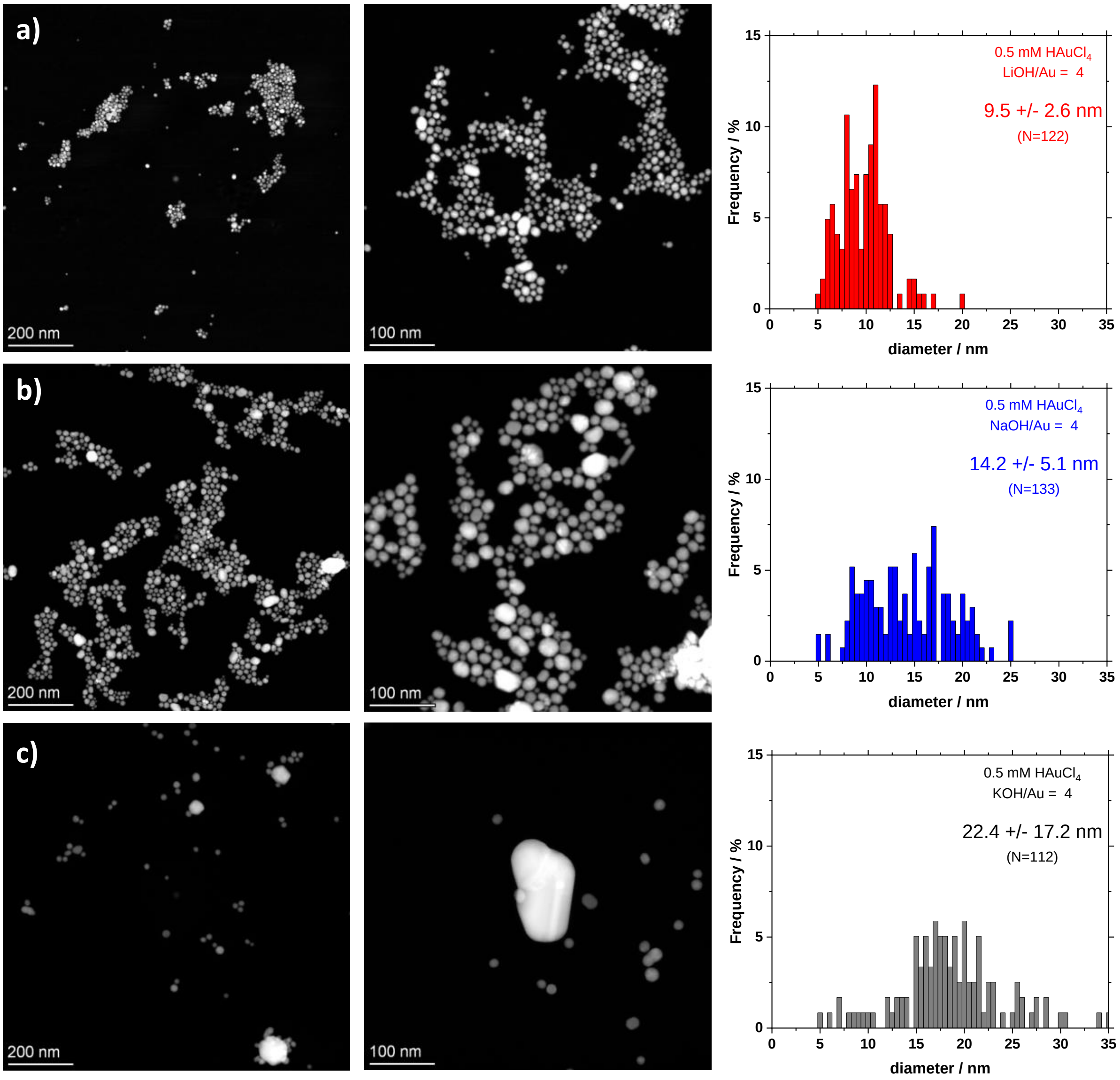

Figure S30. STEM micrographs at different magnifications (left-hand side and middle columns) and related size distributions (right-hand side column) for surfactant-free Au NPs obtained using 20 v.% ethanol, 0.5 mM $HAuCl_4$ and a XOH/Au molar ratio of 4 for different bases (a) LiOH, (b) NaOH, or (c) KOH. The corresponding UV-vis data are given in Figure S28a.

The size distribution data related to NaOH/Au molar ratio of 4 and NaCt/Au = 0 are already reported in [36] and are reproduced here to facilitate the comparison with LiCt and KCt.

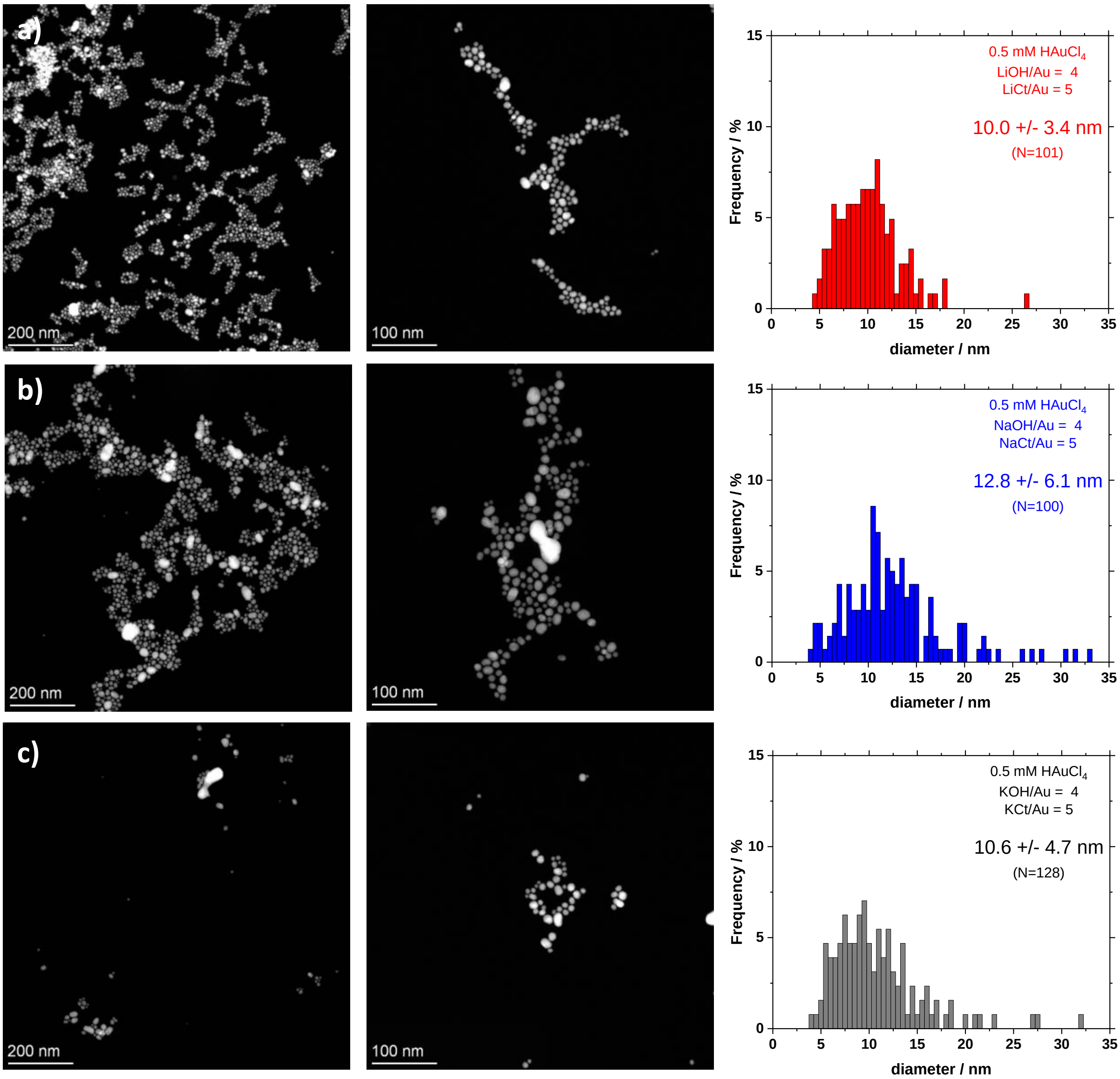

Figure S31. STEM micrographs at different magnifications (left-hand side and middle columns) and related size distributions (right-hand side column) for Au NPs obtained using 20 v.% ethanol, 0.5 mM $HAuCl_4$, a XOH/Au molar ratio of 4 and a XCt/Au molar ratio of 5 for (a) X = Li, (b) X = Na, or (c) X = K. The corresponding UV-vis data are given in Figure S28a.

The size distribution data related to NaOH/Au molar ratio of 4 and NaCt/Au = 5 are already reported in [36] and are reproduced here to facilitate the comparison with LiCt and KCt.

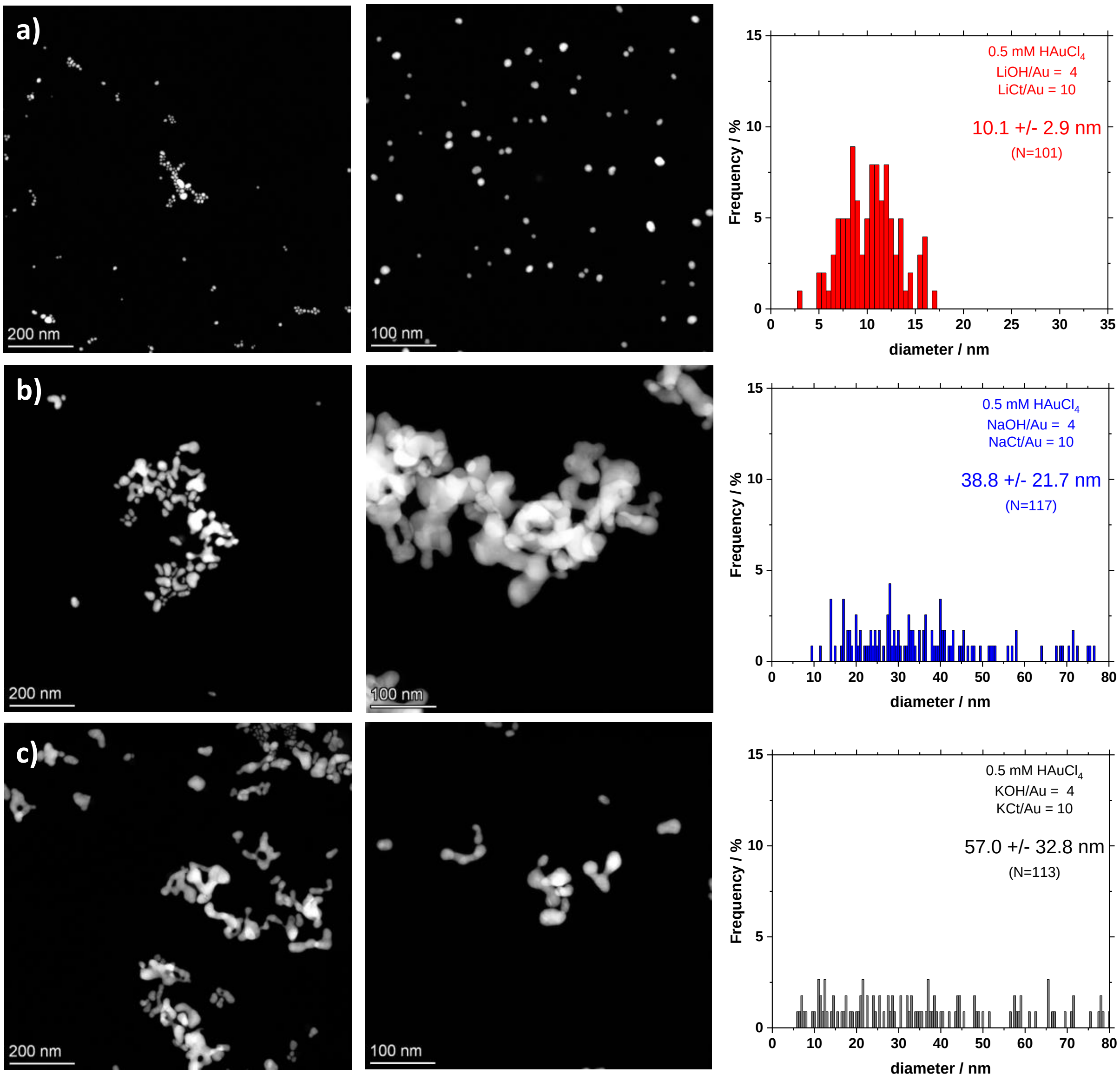

Figure S32. STEM micrographs at different magnifications (left-hand side and middle columns) and related size distributions (right-hand side column) for Au NPs obtained using 20 v.% ethanol, 0.5 mM $HAuCl_4$, a XOH/Au molar ratio of 4 and a XCt/Au molar ratio of 10 for (a) X = Li, (b) X = Na, or (c) X = K. The corresponding UV-vis data are given in Figure S28a.

The size distribution data related to NaOH/Au molar ratio of 4 and NaCt/Au = 10 are already reported in [36] and are reproduced here to facilitate the comparison with LiCt and KCt.

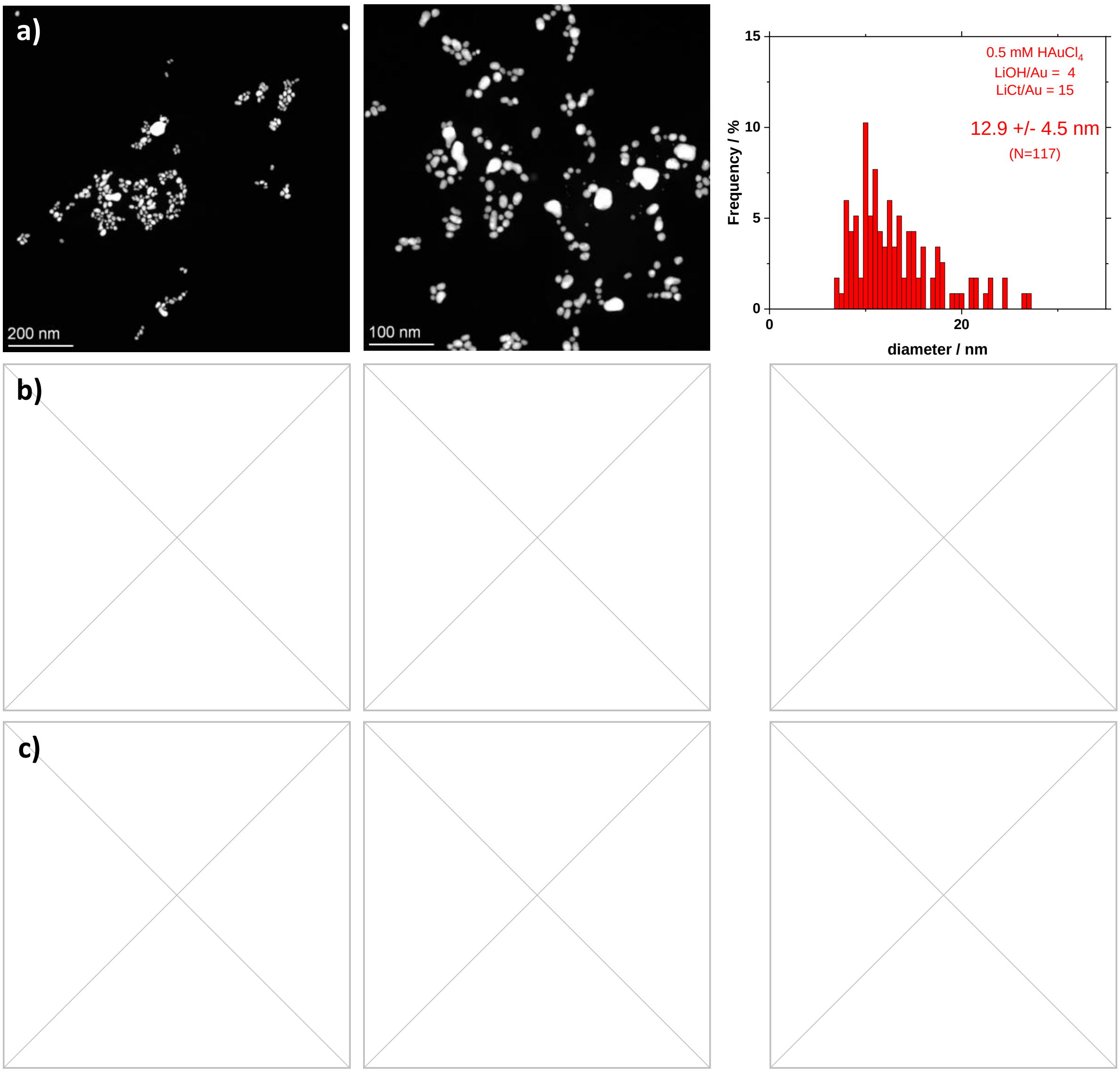

Figure S33. STEM micrographs at different magnifications (left-hand side and middle columns) and related size distributions (right-hand column) for Au NPs obtained using 20 v.% ethanol, 0.5 mM $HAuCl_4$, a XOH/Au molar ratio of 4 and a XCt/Au molar ratio of 15 for (a) X = Li, (b) X = Na (no data could be retrieved), or (c) X = K (no data could be retrieved). Only the samples with X = Li led to dispersions stable enough relevant for further STEM characterization. The corresponding UV-vis data are given in Figure S28a.

After centrifugation as described in **section 5.3.5**, the NPs prepared using LiOH are relatively more stable (less pronounced decrease in the absorbance at 400 nm after centrifugation) than those obtained using NaOH or KOH, see **Figure S34**. This trend is especially clear at higher XCt concentrations and higher $HAuCl_4$ concentrations. This can be attributed to the relatively smaller sizes of NPs obtained with Li-based chemicals.

At a given XCt/Au molar ratio, the relative stability tend to decrease with Li > Na > K and this trend is more pronounced at higher XCt/Au molar ratio. The difference between Na and K is not always pronounced but the use of LiCt shows a consistent improvement in stability compared to NaOH/NaCt and KOH/KCt.

For a given XCt, as the XCt/Au molar ratio increases, the NPs tend to be less stable. This can be attributed in the case of LiCt to stronger Ct-Ct interaction that might favor the agglomeration of the NPs, more likely to be centrifuged away (given that the size obtained is always around 10-15 nm regardless of the XCt/Au molar ratio when 0.5 mM $HAuCl_4$ is used). This effect is less pronounced for NaCt and the low $HAuCl_4$ concentration, but rather clear for the higher $HAuCl_4$ concentration. Interestingly, the surfactant-free NPs (XCt/Au = 0) show stability comparable or even higher to those obtained with XCt - with the exception of KCt/Au molar ratio of 5, discussed below.

The use of KCt/Au molar ratio of 5 and 0.5 mM $HAuCl_4$ lead to rather stable colloids (compared to the use of NaCt). This can be attributed to the actual benefits of using a small amount of KCt to control the NPs size towards smaller size. For instance, for 0.5 mM $HAuCl_4$, KCt/Au molar ratio of 0 leads to NPs ca. 20 nm in size, see **Figure S30**, whereas a KCt/Au molar ratio of 5 leads to ca. 10 nm NPs (and NaCt/Au leads to ca. 13 nm NPs), see **Figure S31**. Compared to the surfactant-free synthesis using KOH, there is therefore a small advantage to use a small amounts of KCt. Given that using only KOH does not lead to stable colloids the presence of citrate might favor more stable colloids in that case. Equally, the series of data points using XCt/Au molar ratio of 5 using 0.2 mM $HAuCl_4$ present slightly different trends where the samples obtained using KCt seem the most stable.

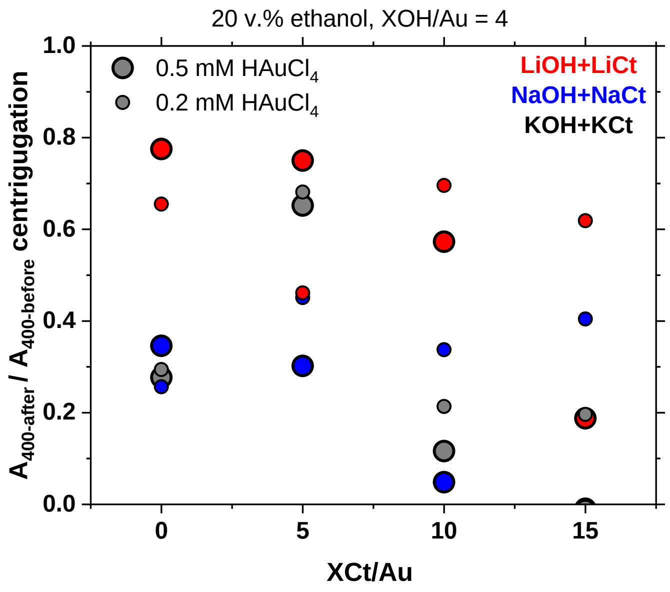

Figure S34. Stability test by centrifugation for Au NPs obtained using 20 v.% ethanol, XOH/Au molar ratio of 4, 0.2 or 0.5 mM $HAuCl_4$, as indicated, and using different XCt/Au molar ratios of 0, 5, 10 or 15, and for which the couple XOH/XCt have the same X, where X = Li, Na or K, as indicated. The data point corresponding to XCt=0 (and XOH/Au molar ratio of 4) are the same as those reported in **Figure S25** and are reported for comparison.

14. Surfactant-free synthesis of Au NPs in alkaline water-ethanol mixtures with various Li-, Na-, K- based chemicals

In this section, the syntheses were performed as described in **section 5.2.8**, following the general surfactant-free approach proposed by Quinson and co-workers.[38] The syntheses were induced using mQ, ethanol, XOH (X = Li, Na, K) and XCt (X = Li, Na, K) or XDS (X = Li, Na) at room temperature. The chemicals were mQ, ethanol, XOH, XCt or XDS and $HAuCl_4$, as indicated. The results show the effects of the cations on the syntheses of Au NPs obtained using alkaline water-ethanol mixtures with both a base and/or citrates or dodecylsulfate at different concentrations of gold precursor and additives.

The results in **Figure S35** combine results from **sections 11** and **13** presented under the form of a 2D plot together with data related to the use of XDS. It is observed that the samples combining smaller NP size (based on lower λ_{spr} values) and higher stability (based on higher A_{380}/A_{800} values) tend to be obtained using Li-based chemicals whereas K-based chemicals lead to overall a poorer control over the colloidal NP properties. Na-based chemicals lead to NP falling in between the two.

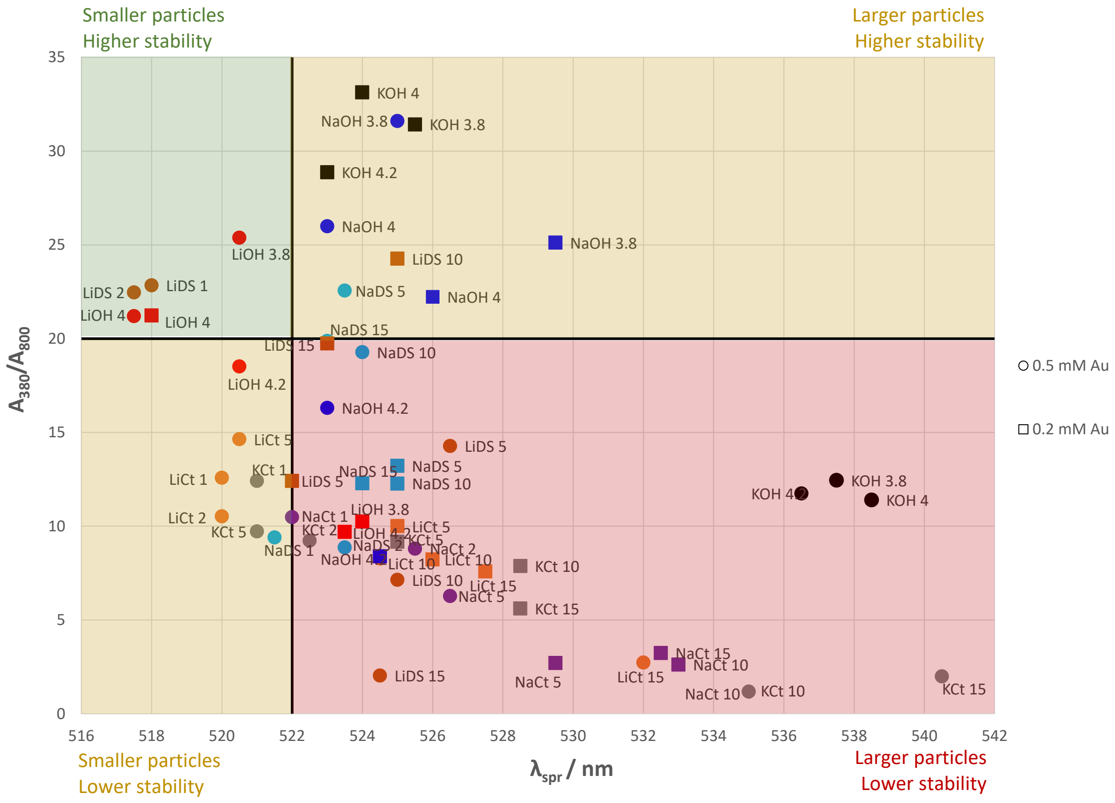

Figure S35. Overview of the size-stability (λ_{spr} – A_{380}/A_{800}) parameter space retrieved from UV–vis characterization for Au NPs obtained using different additives in different amounts, as indicated. The samples were prepared in 20 v.% ethanol with 0.5 mM $HAuCl_4$ or 0.2 mM $HAuCl_4$, as indicated. The base use was LiOH, NaOH, KOH, the abbreviation 'XOH YY' corresponds to the XOH used and YY corresponds to the molar ratio between XOH and $HAuCl_4$. For the experiments where an additive was used, i.e. XCt (for X = Li, Na, K) or XDS (for X = Li, Na), the reaction conditions were 20 v.% ethanol and a XOH/Au molar ratio of 4.

The same ‘X’ was used for the additive and the base. The values YY in the name ‘XCt YY’ or ‘XDS YY’ correspond to the moral ratio between the additive and $HAuCl_4$. Note that samples prepared with too high KCt/Au molar ratios or NaCt/Au molar ratios were not stable enough for UV-vis measurements. The nature of the additives is reported with abbreviations and color codes as indicated where orange-red data points correspond to Li-based chemicals used, blue-shade data points correspond to Na-based chemicals and dark-grey data points correspond to K-based chemicals. Data related to Na-based chemicals are reported in [36].

Overall, the use of Li-based chemicals lead to smaller and more stable Au NPs, whereas the use of K-based chemicals lead to larger and less stable Au NPs. The use of Na-based chemicals falls in-between.

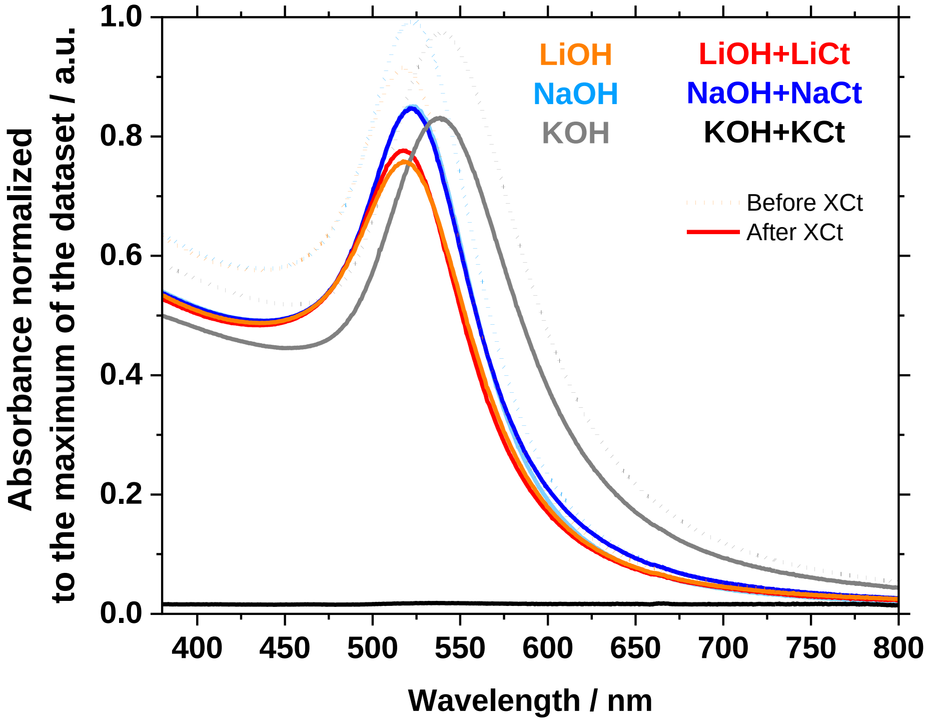

Figure S36. UV-vis spectra of Au NPs prepared using a surfactant-free synthesis of Au NPs using 20 v.% water XOH only and after addition to those same samples XCt with the same X = Li, Na, K, as indicated. There are three datasets to consider. A first dataset was obtained using 0.5 mM $HAuCl_4$, a XOH/Au molar ratio of 4 and ca. 20 v.% ethanol at room temperature and UV-vis measured after 24 hours (orange, light blue and grey, plain lines). A second data set in dotted lines (orange, light blue and grey, dotted lines) was obtained using 0.6 mM $HAuCl_4$, a XOH/Au molar ratio of 4 and ca. 23 v.% ethanol at room temperature and UV-vis measured after 24 hours. A third data set was obtained adding XCt to the second data set so that the XCt/Au molar ratio is 15, which by dilution leads to final concentrations of 0.5 mM $HAuCl_4$, a XOH/Au molar ratio of 4 and ca. 20 v.% ethanol obtained at room temperature and UV-vis measured 24 hours after addition of the XCt (red, blue, dark, plain lines). In other words the second dataset corresponds to the samples before addition of XCt which are more concentrated to be more easily compared with dataset 1 after dilution by addition of XCt.

From **Figure S36**, it is clear that the addition of LiCt or NaCt does not change the stability of preformed surfactant-free Au NPs. However, adding KCt strongly destabilizes the colloids (very low absorbance values close to 0). These results confirm the stabilization role of cations and in this case the (de)stabilization induce with K^+.

See **section 17** for a comment on why mixtures of different cations were not used.

15. Surfactant-free synthesis of Au NPs towards higher $HAuCl_4$ concentrations

See **section 5.2.9** for experimental procedures for those experiments performed at the MAX IV, Lund, Sweden, synchrotron facilities (beamtime ID 20240084). An overview of the samples considered is proposed in **Table S5** and **Table S6**. In all cases, Li-mediated syntheses lead to more stable NPs.

Table S5. Overview of the various samples prepared towards high concentrations of $HAuCl_4$ using LiCt and NaCt. The synthesis was induced at room temperature. The total volume was 3 mL. The stability was assessed by the naked eye if the solutions was looking as stable colloids.

**$HAuCl_4$** mM	**XCt** (X=Li, Na)	**XCt** mM	**XCt/Au**	**Relative stability**
2	Li	40	20	Stable more than a day
3		60		Stable more than a day
3.5		70		Stable for few hours
4		80		Collapsed after 2 h
5		100		Collapsed after 2 h
2	Na	40	20	Collapsed after 2 h
3		60		Collapsed after 2 h
4		80		Collapsed after 2 h
5		100		Collapsed after 2 h

For the citrate-based syntheses, **Table S5**, more stable Au NP colloidal dispersion were obtained when LiCt was used compared to NaCt for a same concentration of $HAuCl_4$ and a same concentration of XCt. A week after the synthesis was performed, the solutions were not stable enough over time to allow for further characterization.

Table S6. Overview of the various samples prepared towards high concentrations of $HAuCl_4$ using LiOH and NaOH. The total volume was 3 mL or 5 mL, as indicated, the solution consisted of 20 v.% ethanol in 80 v.% mQ (before taking into account volume contraction). The stability was assessed by the naked eye if the solutions was looking as stable colloids.

**$HAuCl_4$** mM	**XOH** (X=Li, Na)	**XOH** mM	**XCt/Au**	**Volume*** mL	**Relative stability**
2	Li	8	4	3	Still stable after a week
2.5		10		3	Still stable after a week
3		12		3	Still stable after a week
4		16		3	Still stable after a few days
5		20		3	Collapsed after few hours
6		24		3	Collapsed after few hours
0.5	Na	2	4	5	Still stable after a week
1		4		5	Still stable after a week
2		8		5	Still stable after a week
2.5		10		3	Collapsed after 2 days
3		12		5	Collapsed after 2 days
4		16		5	Collapsed after 2 days
5		20		5	Collapsed after 2 days
6		24		5	Collapsed after 2 days

***** in our experience, the volume does not have a strong effect on the outcome of the synthesis.[38,44]

For the XOH-based syntheses mediated by ethanol, **Table S6**, more stable Au NP colloidal dispersions are obtained when LiOH is used compared to NaOH for a same concentration of $HAuCl_4$ and a same concentration of XOH. Stable colloidal dispersions are obtained for $HAuCl_4$ concentrations of 2 mM or less when NaOH is used, whereas when LiOH is used stable colloidal dispersions are obtained for up to 3-4 mM (although the dispersions obtained with 4 mM $HAuCl_4$ are not as stable as those obtained with 3 mM and this is why no further characterization could be performed on this specific sample). Interestingly, the surfactant-free syntheses lead to more stable Au NPs at high $HAuCl_4$ concentrations than the citrate-mediated syntheses, which allowed further characterization detailed in **Figure S37** and **Figure S38**. Based on UV-vis and STEM data, the Li-mediated syntheses lead to smaller and more stable Au NPs than the Na-mediated counterpart. As the concentration of $HAuCl_4$ increases, the size of the Au NPs tend to increase for the Li-mediated synthesis.

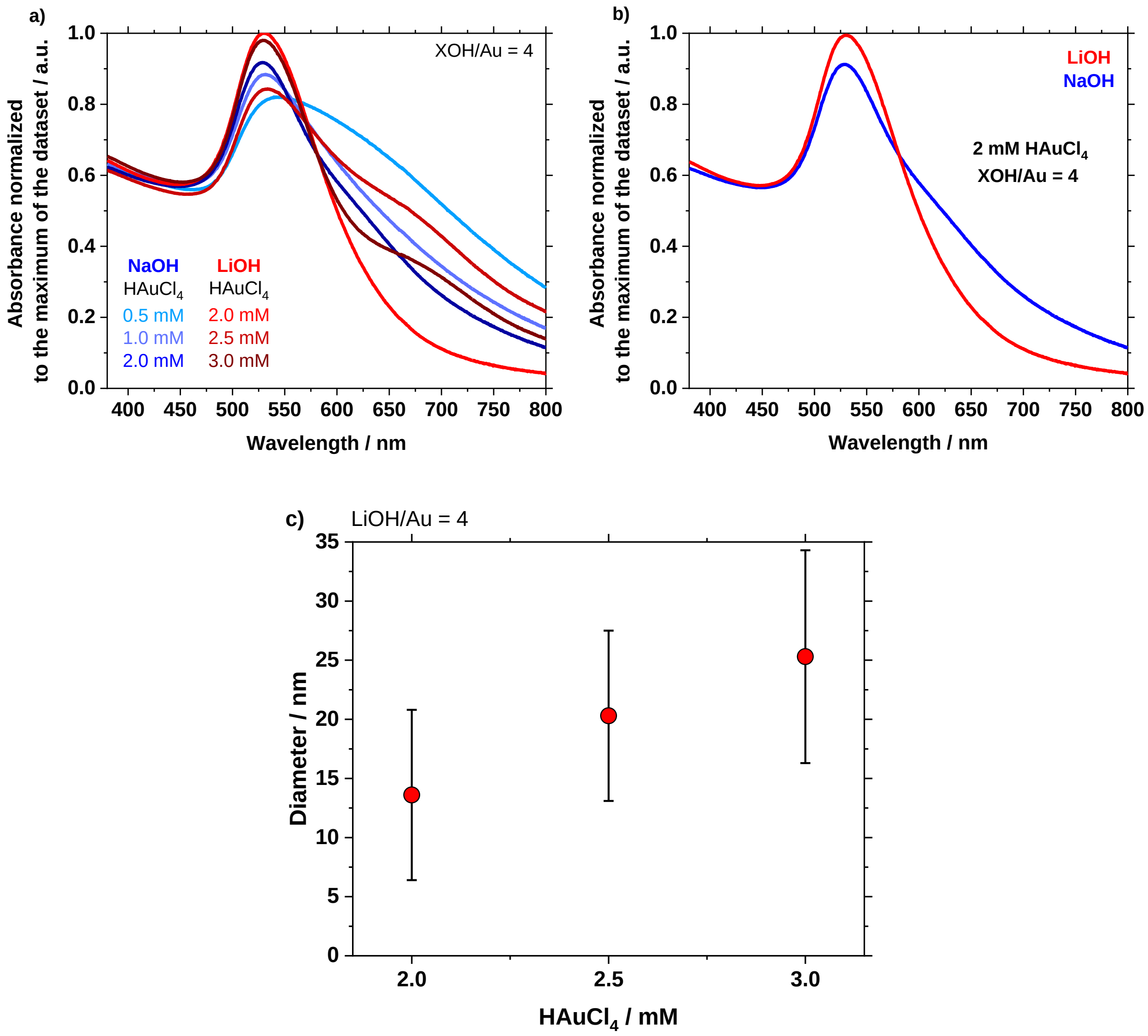

Figure S37. UV-vis spectra of Au NP dispersions prepared with higher concentrations of $HAuCl_4$, as indicated, using LiOH or NaOH, as indicated, a week after synthesis. (b) Selected UV-vis spectra for Au NPs obtained using 2 mM $HAuCl_4$, for dispersions obtained using 2 mM $HAuCl_4$ and 8 mM LiOH or NaOH, as indicated. (c) Diameter of Au NPs as a function of the concentration of $HAuCl_4$ for NPs obtained using a surfactant-free approach consisting of adding $HAuCl_4$ to an alkaline mixture of water and ethanol at room temperature without the need for any other additives. The LiOH/Au molar ratio was 4 in all cases and the samples characterized a week after synthesis. The related STEM data are given in Figure S38.

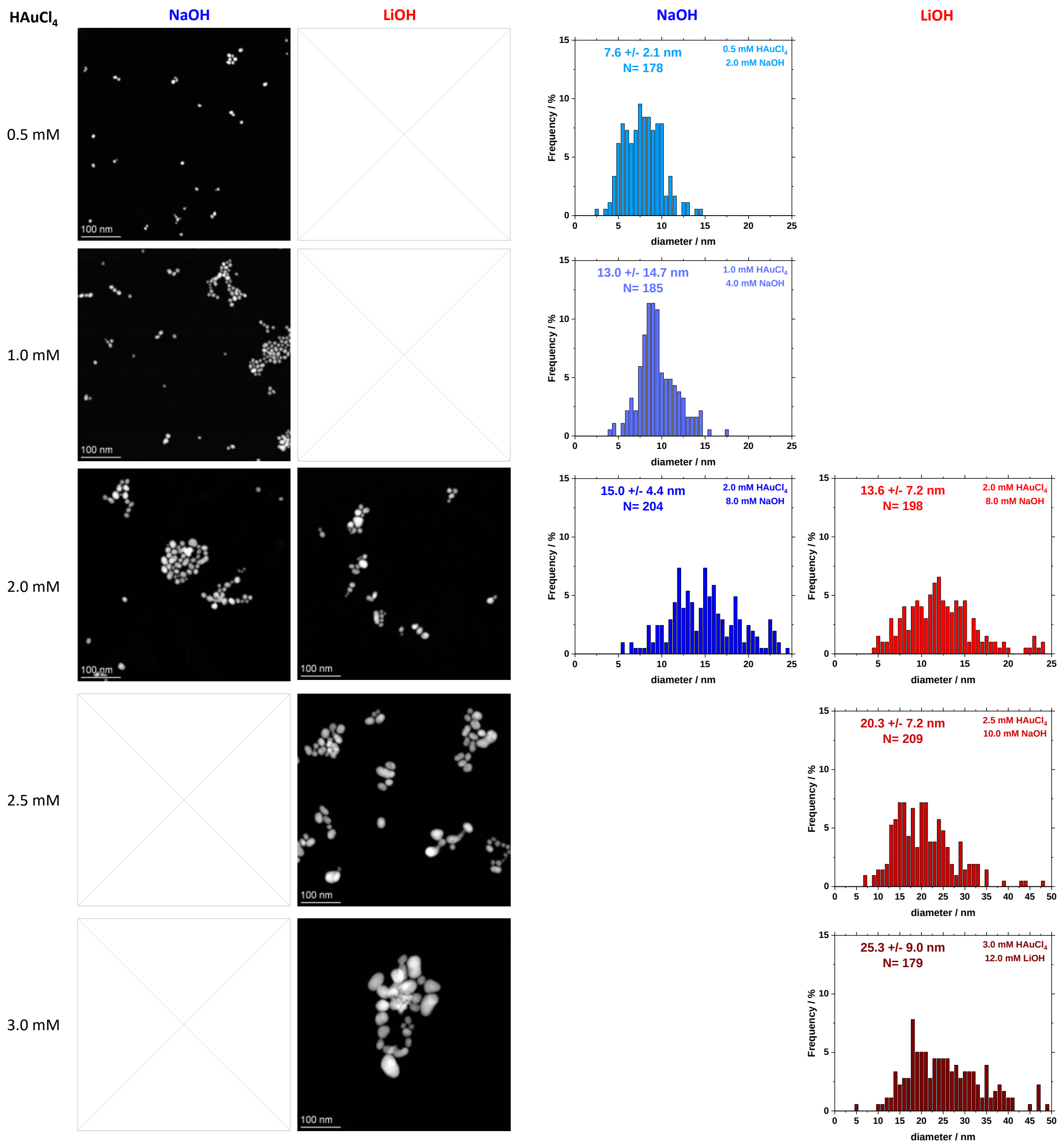

Figure S38. Selected STEM micrographs (first two columns on the left) of Au NPs prepared with higher concentrations of $HAuCl_4$, as indicated, using NaOH or LiOH, as indicated, a week after synthesis for dispersions obtained using a XOH/Au molar ratio of 4 in all cases. The corresponding size distributions are also reported (last two columns on the right). The related UV-vis data are given in Figure S37a.

16. Electron microscopy

The samples in this section were obtained for the same experimental conditions as the sample detailed in **Figure 4** in the main manuscript. The difference is that the sample in **Figure 4** was prepared at the MAX IV synchrotron facilities, Lund, Sweden, whereas the samples discussed below were obtained in our laboratory. In all cases the samples were obtained using 3 mM $HAuCl_4$ and LiCt/Au molar ratio of 20 and left to react at room temperature for a day.

Figure S39 shows 4D-STEM scan reconstructed images of a decahedral NP near a high-symmetry zone axis orientation. Five unique crystallographic orientations are identifiable in the scan, with virtual dark-field (VDF) images displayed in the top row, and the approximate domain extent marked by colored lines. The bottom row features the grain-averaged diffraction pattern, with virtual aperture placement marked to reconstruct the corresponding VDF image. The five different domains and their relative orientations within the NP support the claim of decahedral morphology due to five-fold twinning.[80]

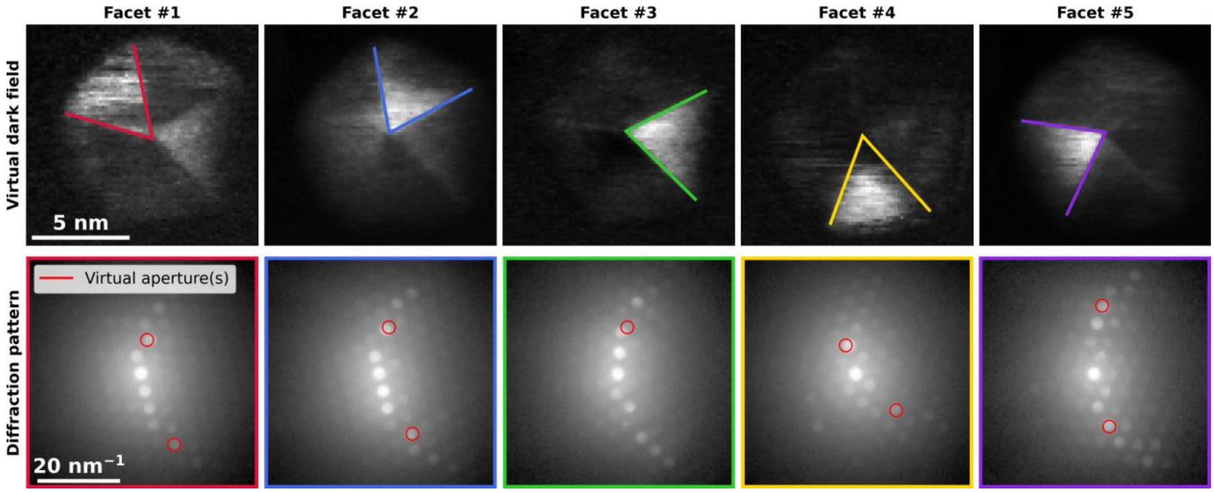

Figure S39. 4D-STEM virtual dark field images and diffraction data from the five domains of a decahedral NP.

For statistical analysis of the NPs, several thousand NPs were imaged with conventional STEM, utilizing the BF, ADF, and HAADF detectors. From these images, a few cropped NPs have been selected that exhibit the five-region decahedral morphology, as shown in **Figure S40**. The STEM-BF micrographs are displayed in the top row, and the corresponding STEM-ADF micrographs are shown in the bottom row. Lines have been drawn to more clearly distinguish the domains of the NPs. Some of the particles appear elongated and asymmetrical, likely due to the presence of BCO/FCC hybrid regions making up the decahedral structure.[81] Alternatively, an apparent distortion may be present due to the NPs being on tilt relative to the electron beam direction.

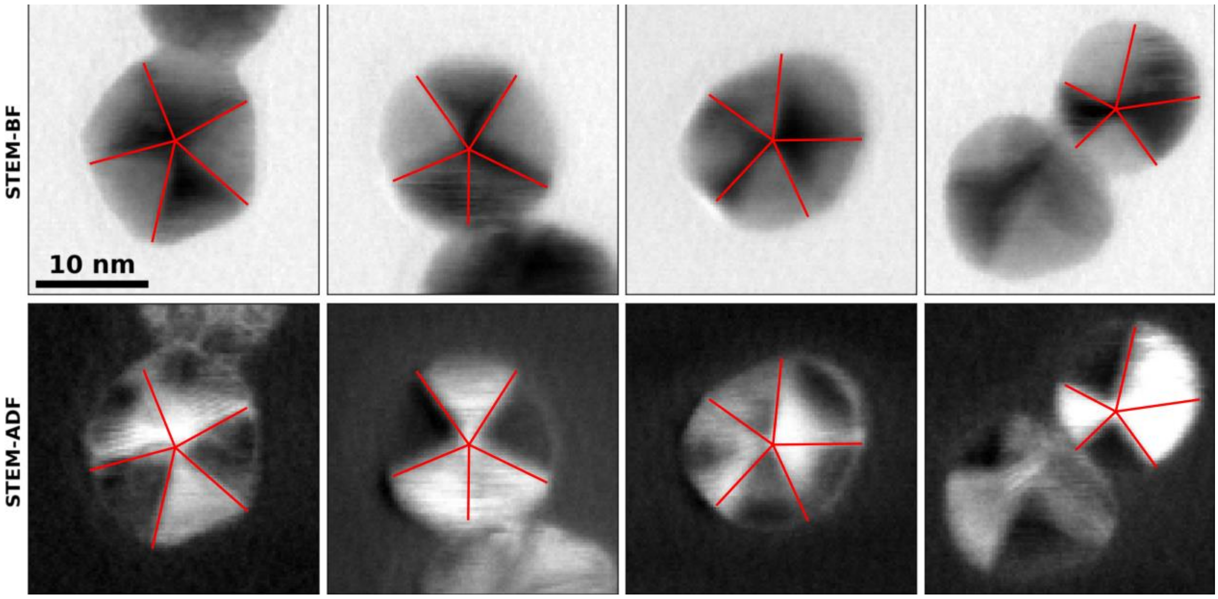

Figure S40. STEM-bright field and annular dark-field micrographs of selected NPs showing five-regioned contrast corresponding to decahedral morphologies. All micrographs were recorded at the same magnification and therefore the same scale bar applies to all micrographs.

From the STEM-HAADF micrographs of the NPs, automatic instance segmentation was performed using the NP-SAM software tool.[82] Over 3000 NPs were segmented, and the diameter distribution is plotted in **Figure S41**. The mean diameter is found to be 15.9 nm, with a standard deviation of 2.2 nm.

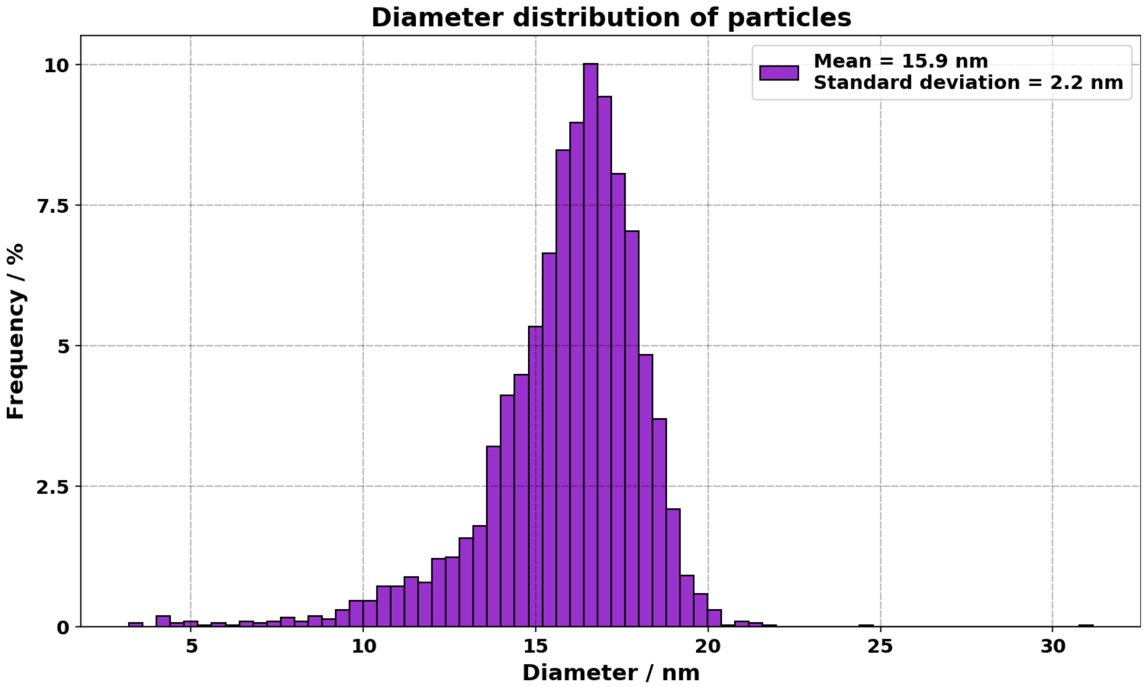

Figure S41. Diameter distribution plot of NPs, as found by instance segmentation of STEM-HAADF images via NP-SAM. 3057 particles were analyzed.

The electron microscopy results confirm the decahedral structure of the NPs and the presence of smaller size NPs as anticipated from X-ray total scattering measurements in **Figure 4**. The slightly larger average size retrieve can come from the lack of size or twinning distributions in the PDF modelling or the fact that the PDF is sensitive to the crystal structure whereas STEM is sensitive to the actual *physical* size / overall diameter of the NPs. Moreover the two batches considered in **Figure 4** and above were obtained in different laboratories so potentially under slightly different conditions (although the experimental conditions used in terms of chemical concentrations and general procedure were identical).

17. Other possible cation effect(s)

In this section, possible sources of cation effects not investigated for this first report but relevant to be explored in future studies based on the presented results are flagged.

- To control Au NP syntheses, pH has been intensively studied as a key factor although some authors prefer to tune the XCt/Au molar ratio.[1,7] We here chose to focus on different Additive/Au molar ratios across different type of syntheses, to prefer a synthetic protocol using as-received chemicals and as-prepared solutions without the need for pH adjustment. It cannot be excluded that the actual concentration of citrate in the stock solutions differ slightly and/or the *active* concentration of citrate differs between LiCt, NaCt, KCt.[22,61]

Nevertheless, by establishing the effect of monovalent cations in three different syntheses and their hybrid, we expect those potential effects to be minor and therefore that the cation effect(s) detailed account for the results presented. The effect of the cation is also assessed by using different sources of cations (XCt, XBH_4, XOH).
It is worth pointing that the general trends align with the results presented in a BSc thesis,[22] where the latter had a specific focus on a given concentration of Au at 0.007 mM and a XCt of 0.21 mM (XCt/Au molar ratio of 30, for a very low $HAuCl_4$ final concentration) and the reaction performed for three hours in 100 mL at 96 °C to obtain seeds. The focus of the study was on the subsequent growth of the NPs from the seeds at fixed pH of 6.7 or 7.3 (with ca. 0.008 mM of Au). The NPs were characterized by TEM and analytical disc centrifugation.
Therefore, it is encouraging that very different approaches (the ones detailed here and the one in a previous BSc thesis[22]) somehow confirm that *stabilization* (this work) or *agglomeration* (BSc thesis[22]) decreases with Li^+ > Na^+ / K^+.

- In a previous BSc thesis the lower ion mobility of Li^+, compared to Na^+, itself showing a lower mobility than K^+, was suggested to lead to less negative reduction potential.[22,83] Refining the effect of cation on the redox-properties of XCt could be a longer term goal.

- Experiments with mixtures of cations are not reported here due to their more complex nature (e.g. using NaOH as base and KCt), because the relative ratios of the species make the interpretation of results more challenging. For instance, the actual availability of the cations might not relate one-to-one to the concentrations of the X-based species due for instance to different interactions between X^+ and BH_4^-, Ct^- or OH^-. Approaches mixing cations could lead to new knobs to tune even further the syntheses.

- In addition, the possible effects of cations from the precursors: $LiAuCl_4$, $NaAuCl_4$ or $KAuCl_4$,[22] is here not considered to prefer $HAuCl_4$ that is more widely reported in the literature and that can be used as a unique precursor source to study the effect(s) of the cations only. However, it can be expected that any source of cations may play a role in the stability of the colloids, including from the precursor.

References

1 Wuithschick, M. *et al.* Turkevich in New Robes: Key Questions Answered for the Most Common Gold Nanoparticle Synthesis. *ACS Nano* **9**, 7052-7071 (2015). https://doi.org/10.1021/acsnano.5b01579
2 Borowskaja, D. Zur Methodik der Goldsolbereitung. *Ztschr. f. Immunitatsforsch. u. exper. Therap.* **82**, 178-182 (1934).
3 Dykman, L. A. & Khlebtsov, N. G. Methods for chemical synthesis of colloidal gold. *Russian Chemical Reviews* **88**, 229-247 (2019). https://doi.org/10.1070/rcr4843
4 De Souza, C. D., Nogueira, B. R. & Rostelato, M. Review of the methodologies used in the synthesis gold nanoparticles by chemical reduction. *Journal of Alloys and Compounds* **798**, 714-740 (2019). https://doi.org/10.1016/j.jallcom.2019.05.153
5 Ribeiro, K. C., Fernandes, L., Bechlin, M. A., Turchiello, R. F. & Gomez, S. L. Effect of the Excitation Wavelength on the Photochemical Synthesis of Citrate-Capped Au Nanoparticles. *Brazilian Journal of Physics* **53**, 41 (2023). https://doi.org/10.1007/s13538-022-01251-z
6 Kim, J. H., Twaddle, K. M., Hu, J. Y. & Byun, H. Sunlight-Induced Synthesis of Various Gold Nanoparticles and Their Heterogeneous Catalytic Properties on a Paper-Based Substrate. *ACS Applied Materials & Interfaces* **6**, 11514-11522 (2014). https://doi.org/10.1021/am503745w
7 Shi, L., Buhler, E., Boue, F. & Carn, F. How does the size of gold nanoparticles depend on citrate to gold ratio in Turkevich synthesis? Final answer to a debated question. *Journal of Colloid and Interface Science* **492**, 191-198 (2017). https://doi.org/10.1016/j.jcis.2016.10.065
8 Tyagi, H., Kushwaha, A., Kumar, A. & Aslam, M. A Facile pH Controlled Citrate-Based Reduction Method for Gold Nanoparticle Synthesis at Room Temperature. *Nanoscale Research Letters* **11**, 362 (2016). https://doi.org/10.1186/s11671-016-1576-5
9 Uppal, M., Kafizas, A., Lim, T. & Parkin, I. The extended time evolution size decrease of gold nanoparticles formed by the Turkevich method. *New Journal of Chemistry* **34**, 1401-1407 (2010). https://doi.org/10.1039/b9nj00745h
10 Turkevich, J., Stevenson, P. C. & Hillier, J. A study of the nucleation and growth processes in the synthesis of colloidal gold. *Discussions of the Faraday Society*, 55-75 (1951). https://doi.org/10.1039/df9511100055
11 Kimling, J. *et al.* Turkevich method for gold nanoparticle synthesis revisited. *Journal of Physical Chemistry B* **110**, 15700-15707 (2006). https://doi.org/10.1021/jp061667w
12 Ojea-Jimenez, I., Bastus, N. G. & Puntes, V. Influence of the Sequence of the Reagents Addition in the Citrate-Mediated Synthesis of Gold Nanoparticles. *Journal of Physical Chemistry C* **115**, 15752-15757 (2011). https://doi.org/10.1021/jp2017242
13 Schulz, F. *et al.* Little Adjustments Significantly Improve the Turkevich Synthesis of Gold Nanoparticles. *Langmuir* **30**, 10779-10784 (2014). https://doi.org/10.1021/la503209b
14 Huang, H. *et al.* Continuous flow synthesis of ultrasmall gold nanoparticles in a microreactor using trisodium citrate and their SERS performance. *Chemical Engineering Science* **189**, 422-430 (2018). https://doi.org/10.1016/j.ces.2018.06.050
15 Quinson, J. Room Temperature Surfactant-Free Syntheses of Gold Nanoparticles in Alkaline Mixtures of Water and Alcohols: A Model System to Introduce Nanotechnology and Green Chemistry to Future Chemists and Engineers. *Journal of Chemical Education* **100**, 3612-3619 (2023). https://doi.org/10.1021/acs.jchemed.3c00492
16 Sivaraman, S. K., Kumar, S. & Santhanam, V. Monodisperse sub-10 nm gold nanoparticles by reversing the order of addition in Turkevich method - The role of chloroauric acid. *Journal of Colloid and Interface Science* **361**, 543-547 (2011). https://doi.org/10.1016/j.jcis.2011.06.015
17 Panariello, L. *et al.* Highly reproducible, high-yield flow synthesis of gold nanoparticles based on a rational reactor design exploiting the reduction of passivated Au(III). *Reaction Chemistry & Engineering* **5**, 663-676 (2020). https://doi.org/10.1039/c9re00469f

18 Li, C. F., Li, D. X., Wan, G. Q., Xu, J. & Hou, W. G. Facile synthesis of concentrated gold nanoparticles with low size-distribution in water: temperature and pH controls. *Nanoscale Research Letters* **6**, 440 (2011). https://doi.org/10.1186/1556-276x-6-440
19 Piella, J., Bastus, N. G. & Puntes, V. Size-Controlled Synthesis of Sub-10-nanometer Citrate-Stabilized Gold Nanoparticles and Related Optical Properties. *Chemistry of Materials* **28**, 1066-1075 (2016). https://doi.org/10.1021/acs.chemmater.5b04406
20 Salley, D. *et al.* A nanomaterials discovery robot for the Darwinian evolution of shape programmable gold nanoparticles. *Nature Communications* **11**, 2771 (2020). https://doi.org/10.1038/s41467-020-16501-4
21 Zabetakis, K., Ghann, W., Kumar, S. & Daniel, M. Effect of high gold salt concentrations on the size and polydispersity of gold nanoparticles prepared by an extended Turkevich-Frens method. *Gold Bulletin* **45**, 203-211 (2012). https://doi.org/10.1007/s13404-012-0069-2
22 Closson, A. B. Citrate's Counter Ions and pH Effect on Gold Nanoparticle Growth Kinetics, Honors College, University of Maine, 374 (2016). https://digitalcommons.library.umaine.edu/honors/374/
23 Fathi, F. & Kraatz, H. Effects of surfactants on electrochemically prepared Ag nanostructures. *Analyst* **138**, 5920-5925 (2013). https://doi.org/10.1039/c3an00933e
24 Fantechi, E. *et al.* Assessing the hyperthermic properties of magnetic heterostructures: the case of gold-iron oxide composites. *Interface Focus* **6**, 20160058 (2016). https://doi.org/10.1098/rsfs.2016.0058
25 Ghosh, G., Panicker, L., Kumar, N. N. & Mallick, V. Surface Plasmon Resonance of Counterions coated Charged Silver Nanoparticles and Application in Bio-interaction. *Materials Research Express* **5**, 055005 (2018). https://doi.org/10.1088/2053-1591/aabe67
26 Liz-Marzan, L. M. Gold nanoparticle research before and after the Brust-Schiffrin method. *Chemical Communications* **49**, 16-18 (2013). https://doi.org/10.1039/c2cc35720h
27 Perala, S. & Kumar, S. On the Mechanism of Metal Nanoparticle Synthesis in the Brust-Schiffrin Method. *Langmuir* **29**, 9863-9873 (2013). https://doi.org/10.1021/la401604q
28 Deraedt, C. *et al.* Sodium borohydride stabilizes very active gold nanoparticle catalysts. *Chemical Communications* **50**, 14194-14196 (2014). https://doi.org/10.1039/c4cc05946h
29 Brust, M., Walker, M., Bethell, D., Schiffrin, D. J. & Whyman, R. Synthesis of thiol-derivatized gold nanoparticles in a 2-phase liquid-liquid system. *Journal of the Chemical Society-Chemical Communications*, 801-802 (1994). https://doi.org/10.1039/c39940000801
30 Corbierre, M. & Lennox, R. Preparation of thiol-capped gold nanoparticles by chemical reduction of soluble Au(I)-thiolates. *Chemistry of Materials* **17**, 5691-5696 (2005). https://doi.org/10.1021/cm051115a
31 Wu, J. *et al.* Synthesis and Characterization of Gold Nanoparticles Based on Low Generational Triethylene Glycol-Polyamidoamine Dendrimers. *Journal of Nanoscience and Nanotechnology* **10**, 2181-2184 (2010). https://doi.org/10.1166/jnn.2010.2127
32 Agarwal, S., Reddy, S. & Dhayal, M. Ultra-small gold nanoparticles synthesized in aqueous solution and their application in fluorometric collagen estimation using bi-ligand functionalization. *RSC Advances* **4**, 18250-18256 (2014). https://doi.org/10.1039/c3ra48047j
33 Gomes, J. F. *et al.* New insights into the formation mechanism of Ag, Au and AgAu nanoparticles in aqueous alkaline media: alkoxides from alcohols, aldehydes and ketones as universal reducing agents. *Physical Chemistry Chemical Physics* **17**, 21683-21693 (2015). https://doi.org/10.1039/c5cp02155c
34 Rasmussen, D. R., Nielsen, M. F. & Quinson, J. Room Temperature Surfactant-Free Synthesis of Gold Nanoparticles in Alkaline Ethylene Glycol. *Chemistry* **5**, 900-911 (2023). https://doi.org/10.3390/chemistry5020061
35 Parveen, R., Ullah, S., Sgarbi, R. & Tremiliosi, G. One-pot ligand-free synthesis of gold nanoparticles: The role of glycerol as reducing-cum-stabilizing agent. *Colloids and Surfaces a-Physicochemical and Engineering Aspects* **565**, 162-171 (2019). https://doi.org/10.1016/j.colsurfa.2019.01.005

36 Varga, M. & Quinson, J. Fewer, but better: on the benefits for surfactant-free colloidal syntheses of nanomaterials. *ChemistrySelect* **10**, e202404819 (2025). https://doi.org/10.1002/slct.202404819
37 Quinson, J., Kunz, S. & Arenz, M. Surfactant-free colloidal syntheses of precious metal nanoparticles for improved catalysts. *ACS Catalysis* **13**, 4903–4937 (2023). https://doi.org/10.1021/acscatal.2c05998
38 Quinson, J. *et al.* Surfactant-free colloidal syntheses of gold-based nanomaterials in alkaline water and mono-alcohol mixtures. *Chemistry of Materials* **35**, 2173–2190 (2023). https://doi.org/10.1021/acs.chemmater.3c00090
39 Quinson, J., Nielsen, T. M., Escudero-Escribano, M. & Jensen, K. M. Ø. Room temperature syntheses of surfactant-free colloidal gold nanoparticles: the benefits of mono-alcohols over polyols as reducing agents for electrocatalysis. *Colloids and Surfaces A: Physicochemical and Engineering Aspects* **675**, 131853 (2023). https://doi.org/10.1016/j.colsurfa.2023.131853
40 Reichenberger, S., Marzun, G., Muhler, M. & Barcikowski, S. Perspective of Surfactant-free Colloidal Nanoparticles in Heterogeneous Catalysis. *ChemCatChem* **11**, 4489-4518 (2019). https://doi.org/10.1002/cctc.201900666
41 Tang, J. Q. & Man, S. Q. Green Synthesis of Colloidal Gold by Ethyl Alcohol and NaOH at Normal Temperature. *Rare Metal Materials and Engineering* **42**, 2232-2236 (2013).
42 Quinson, J. On the Importance of Fresh Stock Solutions for Surfactant-Free Colloidal Syntheses of Gold Nanoparticles in Alkaline Alcohol and Water Mixtures. *Inorganics* **11**, 140 (2023). https://doi.org/10.3390/inorganics11040140
43 Quinson, J. Influence of the alcohol and water grades on surfactant-free colloidal syntheses of gold nanoparticles in alkaline water-alcohol mixtures. *Gold Bulletin* **57**, 27-31 (2024). https://doi.org/10.1007/s13404-024-00345-7
44 Rasmussen, D. R., Lock, N. & Quinson, J. Lights on the synthesis of surfactant-free colloidal gold nanoparticles in alkaline mixtures of alcohols and water. *ChemSusChem* **18**, e202400763 (2025). https://doi.org/10.1002/cssc.202400763
45 Panagopoulos, D., Asghari Alamdari, A. & Quinson, J. Surfactant-free colloidal gold nanoparticles: room temperature synthesis, size control and opportunities for catalysis. *Materials Today Nano* **29**, e202400763 (2025). https://doi.org/10.1016/j.mtnano.2025.100600
46 Oncsik, T., Trefalt, G., Borkovec, M. & Szilagyi, I. Specific Ion Effects on Particle Aggregation Induced by Monovalent Salts within the Hofmeister Series. *Langmuir* **31**, 3799-3807 (2015). https://doi.org/10.1021/acs.langmuir.5b00225
47 Quinson, J. *et al.* Monovalent Alkali Cations: Simple and Eco-Friendly Stabilizers for Surfactant-Free Precious Metal Nanoparticle Colloids. *ACS Sustainable Chemistry & Engineering* **7**, 13680-13686 (2019). https://doi.org/10.1021/acssuschemeng.9b00681
48 Mathiesen, J. K. *et al.* Chemical insights on the formation of colloidal iridium nanoparticles from in situ X-ray total scattering: Influence of precursors and cations on the reaction pathway. *Journal of the American Chemical Society* **145**, 1769–1782 (2023). https://doi.org/10.1021/jacs.2c10814
49 Pfeiffer, C. *et al.* Interaction of colloidal nanoparticles with their local environment: the (ionic) nanoenvironment around nanoparticles is different from bulk and determines the physico-chemical properties of the nanoparticles. *Journal of the Royal Society Interface* **11**, 20130931 (2014). https://doi.org/10.1098/rsif.2013.0931
50 Strmcnik, D. *et al.* The role of non-covalent interactions in electrocatalytic fuel-cell reactions on platinum. *Nature Chemistry* **1**, 466-472 (2009). https://doi.org/10.1038/nchem.330
51 Lu, Z. *et al.* Predicting Counterion Effects Using a Gold Affinity Index and a Hydrogen Bonding Basicity Index. *Organic Letters* **19**, 5848-5851 (2017). https://doi.org/10.1021/acs.orglett.7b02829
52 Ziefuss, A., Barcikowski, S. & Rehbock, C. Synergism between Specific Halide Anions and pH Effects during Nanosecond Laser Fragmentation of Ligand-Free Gold Nanoparticles. *Langmuir* **35**, 6630-6639 (2019). https://doi.org/10.1021/acs.langmuir.9b00418

53 Lohse, S., Burrows, N., Scarabelli, L., Liz-Marzán, L. & Murphy, C. Anisotropic Noble Metal Nanocrystal Growth: The Role of Halides. *Chemistry of Materials* **26**, 34-43 (2014). https://doi.org/10.1021/cm402384j
54 Hu, S., Huang, P., Wang, J. & Liu, J. Dissecting the Effect of Salt for More Sensitive Label-Free Colorimetric Detection of DNA Using Gold Nanoparticles. *Analytical Chemistry* **92**, 13354-13360 (2020). https://doi.org/10.1021/acs.analchem.0c02688
55 Liu, B. W., Kelly, E. Y. & Liu, J. W. Cation-Size-Dependent DNA Adsorption Kinetics and Packing Density on Gold Nanoparticles: An Opposite Trend. *Langmuir* **30**, 13228-13234 (2014). https://doi.org/10.1021/la503188h
56 Perera, G. *et al.* Counterion Effects on Electrolyte Interactions with Gold Nanoparticles. *Journal of Physical Chemistry C* **120**, 23604-23612 (2016). https://doi.org/10.1021/acs.jpcc.6b07885
57 Hersbach, T. J. P. *et al.* Alkali Metal Cation Effects in Structuring Pt, Rh, and Au Surfaces through Cathodic Corrosion. *ACS Applied Materials & Interfaces* **10**, 39363-39379 (2018). https://doi.org/10.1021/acsami.8b13883
58 Freese, T., Elzinga, N., Heinemann, M., Lerch, M. M. & Feringa, B. L. The relevance of sustainable laboratory practices. *RSC Sustainability* **2**, 1300-1336 (2024). https://doi.org/10.1039/D4SU00056K
59 Li, N., Zhao, P. X. & Astruc, D. Anisotropic Gold Nanoparticles: Synthesis, Properties, Applications, and Toxicity. *Angewandte Chemie-International Edition* **53**, 1756-1789 (2014). https://doi.org/10.1002/anie.201300441
60 Scarabelli, L., Sanchez-Iglesias, A., Perez-Juste, J. & Liz-Marzan, L. M. A "Tips and Tricks" Practical Guide to the Synthesis of Gold Nanorods. *Journal of Physical Chemistry Letters* **6**, 4270-4279 (2015). https://doi.org/10.1021/acs.jpclett.5b02123
61 Liz-Marzan, L. M., Kagan, C. R. & Millstone, J. E. Reproducibility in Nanocrystal Synthesis? Watch Out for Impurities! *ACS Nano* **14**, 6359-6361 (2020). https://doi.org/10.1021/acsnano.0c04709
62 Jæger, F., Pedersen, A. A., Wacherhausen, P. S., Smolska, A. & Quinson, J. Surfactant-free gold nanoparticles synthesized in alkaline water–ethanol mixtures: leveraging lower grade chemicals for size control of active nanocatalysts. *RSC Sustainability* **3**, 2870-2875 (2025). https://doi.org/10.1039/D5SU00213C
63 Roy, A. *et al.* The Huge Role of Tiny Impurities in Nanoscale Synthesis. *ACS Nanoscience Au* **4**, 176-193 (2024). https://doi.org/10.1021/acsnanoscienceau.3c00056
64 Mourdikoudis, S., Pallares, R. M. & Thanh, N. T. K. Characterization techniques for nanoparticles: comparison and complementarity upon studying nanoparticle properties. *Nanoscale* **10**, 12871-12934 (2018). https://doi.org/10.1039/c8nr02278j
65 Grasseschi, D., Ando, R. A., Toma, H. E. & Zamarion, V. M. Unraveling the nature of Turkevich gold nanoparticles: the unexpected role of the dicarboxyketone species. *RSC Advances* **5**, 5716-5724 (2015). https://doi.org/10.1039/c4ra12161a
66 Haiss, W., Thanh, N. T. K., Aveyard, J. & Fernig, D. G. Determination of size and concentration of gold nanoparticles from UV-Vis spectra. *Analytical Chemistry* **79**, 4215-4221 (2007). https://doi.org/10.1021/ac0702084
67 Ye, Y. J., Lv, M. X., Zhang, X. Y. & Zhang, Y. X. Colorimetric determination of copper(II) ions using gold nanoparticles as a probe. *RSC Advances* **5**, 102311-102317 (2015). https://doi.org/10.1039/c5ra20381c
68 Merk, V. *et al.* In Situ Non-DLVO Stabilization of Surfactant-Free, Plasmonic Gold Nanoparticles: Effect of Hofmeister's Anions. *Langmuir* **30**, 4213-4222 (2014). https://doi.org/10.1021/la404556a
69 Hendel, T. *et al.* In Situ Determination of Colloidal Gold Concentrations with UV-Vis Spectroscopy: Limitations and Perspectives. *Analytical Chemistry* **86**, 11115-11124 (2014). https://doi.org/10.1021/ac502053s
70 Panariello, L., Radhakrishnan, A. N. P., Papakonstantinou, I., Parkin, I. P. & Gavriilidis, A. Particle Size Evolution during the Synthesis of Gold Nanoparticles Using In Situ Time-Resolved UV-Vis Spectroscopy: An Experimental and Theoretical Study Unravelling the

Effect of Adsorbed Gold Precursor Species. *Journal of Physical Chemistry C* **124**, 27662-27672 (2020). https://doi.org/10.1021/acs.jpcc.0c07405
71 Larsen, J. C. H., Porsgaard, A. N., Vinding, L., Smolska, A. & Quinson, J. Less chemicals for more controlled syntheses: on the benefits of mixtures of alcohols for room temperature surfactant-free colloidal syntheses of gold nanoparticles. *RSC Sustainability* **3**, 2870-2875 (2025). https://doi.org/10.26434/chemrxiv-2025-ct21t
72 Kieffer, J., Valls, V., Blanc, N. & Hennig, C. New tools for calibrating diffraction setups. *Journal of Synchrotron Radiation* **27**, 558-566 (2020). https://doi.org/10.1107/S1600577520000776
73 Juhas, P., Davis, T., Farrow, C. L. & Billinge, S. J. L. PDFgetX3: a rapid and highly automatable program for processing powder diffraction data into total scattering pair distribution functions. *Journal of Applied Crystallography* **46**, 560-566 (2013). https://doi.org/10.1107/S0021889813005190
74 Banerjee, S. *et al.* Cluster-mining: an approach for determining core structures of metallic nanoparticles from atomic pair distribution function data. *Acta Crystallographica a-Foundation and Advances* **76**, 24-31 (2020). https://doi.org/10.1107/s2053273319013214
75 Anker, A. S. et al. Autonomous nanoparticle synthesis by design. *arXiv* arXiv:2505.13571 [cond-mat.mtrl-sci] Preprint (2025). https://doi.org/10.48550/arXiv.2505.13571
76 Hjorth Larsen, A. *et al.* The atomic simulation environment—a Python library for working with atoms. *Journal of Physics: Condensed Matter* **29**, 273002 (2017). https://doi.org/10.1088/1361-648x/aa680e
77 Scardi, P., Billinge, S. J. L., Neder, R. & Cervellino, A. Celebrating 100 years of the Debye scattering equation. *Acta Crystallographica Section A: Foundations and Advances* **72**, 589-590 (2016). https://doi.org/10.1107/S2053273316015680
78 Debye, P. Zerstreuung von Röntgenstrahlen. *Annalen der Physik* **351**, 809-823 (1915). https://doi.org/10.1002/andp.19153510606
79 Johansen, F. L., Anker, A. S., Friis-Jensen, U., Dam, E. B. & Selvan, R. A GPU-Accelerated Open-Source Python Package for Calculating Powder Diffraction, Small-Angle-, and Total Scattering with the Debye Scattering Equation. *Journal of Open Source Software* **9**, 6024 (2024). https://doi.org/10.21105/joss.06024
80 Johnson, C. L. *et al.* Effects of elastic anisotropy on strain distributions in decahedral gold nanoparticles. *Nature Materials* **7**, 120-124 (2008). https://doi.org/10.1038/nmat2083
81 Nakotte, H. *et al.* Pair distribution function analysis applied to decahedral gold nanoparticles. *Physica Scripta* **92**, 114002 (2017). https://doi.org/10.1088/1402-4896/aa8afd
82 Larsen, R., Villadsen, T., L., Mathiesen, J. K., Jensen, K. M. Ø. & Bøjesen, E. D. NP-SAM: Implementing the Segment Anything Model for Easy Nanoparticle Segmentation in Electron Microscopy Images. ChemRxiv. (2023). 10.26434/chemrxiv-2023-k73qz-v2.
83 Lee, S. & Rasaiah, J. Molecular-dynamics simulatrion of ionic mobility.1. Alkali-metal cations in water at 25 °C. *Journal of Chemical Physics* **101**, 6964-6974 (1994). https://doi.org/10.1063/1.468323
83 Lee, S. & Rasaiah, J. Molecular-dynamics simulatrion of ionic mobility.1. Alkali-metal cations in water at 25 °C. *Journal of Chemical Physics* **101**, 6964-6974 (1994). https://doi.org/10.1063/1.468323
